# Predicting Outcomes in a Sequence of Binary Events: Belief Updating and Gambler's Fallacy Reasoning

**DOI:** 10.1111/cogs.13211

**Published:** 2023-01-21

**Authors:** Kariyushi Rao, Reid Hastie

**Affiliations:** ^1^ Department of Behavioral Science The University of Chicago Booth School of Business Chicago United States; ^2^ Behavioural Science Group Warwick Business School, University of Warwick Coventry United Kingdom

**Keywords:** Binary sequences, Hot hand, Gambler's fallacy, Belief updating, Cognitive biases, Randomness, Rationality

## Abstract

Beliefs like the Gambler's Fallacy and the Hot Hand have interested cognitive scientists, economists, and philosophers for centuries. We propose that these judgment patterns arise from the observer's mental models of the sequence‐generating mechanism, moderated by the strength of belief in an a priori base rate. In six behavioral experiments, participants observed one of three mechanisms generating sequences of eight binary events: a random mechanical device, an intentional goal‐directed actor, and a financial market. We systematically manipulated participants’ beliefs about the base rate probabilities at which different outcomes were generated by each mechanism. Participants judged 18 sequences of outcomes produced by a mechanism with either an unknown base rate, a specified distribution of three equiprobable base rates, or a precise, fixed base rate. Six target sequences ended in streaks of between two and seven identical outcomes. The most common predictions for subsequent events were best described as pragmatic belief updating, expressed as an increasingly strong expectation that a streak of identical signals would repeat as the length of that streak increased. The exception to this pattern was for sequences generated by a random mechanical device with a fixed base rate of .50. Under this specific condition, participants exhibited a bias toward reversal of streaks, and this bias was larger when participants were asked to make a dichotomous choice versus a numerical probability rating. We review alternate accounts for the anomalous judgments of sequences and conclude with our favored interpretation that is based on Rabin's version of Tversky & Kahneman's Law of Small Numbers.

People spend a lot of time trying to predict the future. Perhaps the purest form of these inductive projections occurs when an observer forecasts the next outcome following a sequence of similar binary events: heads or tails on the next coin flip, success or failure of a basketball player's next shot, or whether a company's stock price will rise or fall tomorrow. In these situations, the simplest strategy people follow is to predict “more of the same” (Soetens, Boeur, & Hueting, 1985). A somewhat more sophisticated strategy involves forming an impression of statistical patterns in past outcomes in order to predict more complex past‐to‐future relationships (Restle, 1966). The highest levels of reasoning involve inducing a causal explanation for the pattern in past outcomes, and relying on this causal mental model of the outcome‐generating process to make forecasts for the future (Estes, 1964; Oskarsson, Boven, McClelland, & Hastie, 2009). The focus of the present research is on how these mental models influence forecasts of future outcomes in sequences of binary events.

For example, a gambler forms a mental model of roulette based on her past experience playing the game. This experience leads her to develop prior beliefs about the random pattern of outcomes produced by the wheel, and about the base rate probabilities at which the ball lands in a red or black pocket. A sports fan forms a mental model of basketball players based on his past experience watching games. He believes that athletes are intentional, goal‐directed actors, with some control over their performance outcomes. These abstract mental models are evoked when the gambler tries to predict what pocket the ball will land in next, or when the fan tries to predict whether his favorite player will make or miss his next shot.

We are interested in the effects of the mental models people create for three different classes of outcome‐generating processes (generators): random mechanical devices, intentional (human) actors, and social (financial) market processes. These three classes of generators have drawn the most attention from researchers investigating two different judgment patterns in people's predictions for the next outcome following a sequence of binary events. The first pattern occurs when people *increase* their expectation that a certain type of outcome will occur after observing that outcome repeat several times in a row. For example, the sports fan might show up to a game with a mental model of his favorite player, LeBron James, that includes prior beliefs about LeBron's base rate of success for field goal shots (about .51 at the time of writing). After watching LeBron successfully hit five field goals in a row, the fan *increases* his expectation that LeBron will succeed on his next field goal attempt *above* LeBron's base rate for hitting field goals. This judgment pattern is often called the *hot hand belief*, as many sports fans report that players sometimes enter a “hot” state where their rate of success increases above their career (or season) average. The hot hand judgment pattern is most often observed in people's predictions for intentional goal‐directed actors (Alter & Oppenheimer, 2006; Bar‐Eli, Avugos, & Raab, 2006).

The second pattern occurs when people *decrease* their expectation that a certain type of outcome will occur after observing that outcome repeat several times in a row (Feller, 1968, p. 86ff). For example, the gambler arrives at a roulette table with a mental model of the game that includes prior beliefs about the base rate at which the ball lands in a red‐colored pocket (about .47 in American casinos). After watching the ball land on red 5 times in a row, the gambler *decreases* her expectation that the ball will land in a red pocket on the next spin to a rate *below* the base rate for red hits. This judgment pattern is called the *gambler's fallacy*, because it is frequently observed in casino gambling situations (Croson & Sundali, 2005; Laplace, 1902/1814). The gambler's fallacy judgment pattern reliably occurs for a subset of observers making predictions for future events produced by random mechanical devices (Dohmen, Falk, Huffman, Marklein, & Sunde, 2009). Interestingly, *both* the hot hand *and* gambler's fallacy judgment patterns are observed in people's predictions for financial markets (Conrad & Kaul, 1998; Forbes, 1995; Johnson, Tellis, & Macinnis, 2005).

Researchers have offered a variety of theoretical accounts to explain the hot hand and gambler's fallacy judgment patterns. Few of these accounts offer a unified framework that explains *both* the hot hand *and* the gambler's fallacy patterns. And, whether unified or not, these accounts all leave us with important, unanswered questions. Why is it that the gambler's fallacy pattern occurs most often when people make predictions for random mechanical devices, but the hot hand pattern occurs most often when people make predictions for intentional actors? And, which of these patterns should we expect to emerge when people are making predictions for financial markets? We believe that the answer to these questions is related to beliefs observers have about the base rates of events produced by these different generating mechanisms.

Prior experimental studies comparing people's predictions for different types of generators have focused on qualitative descriptions of those generators (e.g. a random mechanical device, or an intentional, goal‐directed actor). But, researchers have often failed to explicitly specify information about the base rates at which these different types of generators produce outcomes. Generators described as having fixed, well‐known base rates (e.g. coins, dice, and roulette wheels), are often compared to generators with ambiguous base rates (e.g. car salesmen, magicians, and basketball players). In the present studies we systematically disentangle qualitative descriptions of the generator from prior beliefs about the generator's base rate. Our goal is to understand how these two sources of information influence people's predictions for future events. To anticipate our conclusions, we will discover that simple updating of estimated base rates is the primary cognitive process producing increasing expectations of repetition (hot hand) as streak length increases for *all types of generators* when the base rate is uncertain or ambiguous. But, when the base rate is explicit, stationary, and well‐known, many observers exhibit a bias toward reversal of streaks (gambler's fallacy), specifically for random mechanical devices.

This article is organized as follows. In Section 1, we review prior research on people's predictions for future outcomes in sequences of binary events. In Section 2, we provide an overview of the present studies. Sections 3, 4, and 5 present the methods and results from six behavioral experiments. Section 6 concludes by discussing the implications and limitations of the present research.

## 1 Theoretical and Empirical Context for the Present Research

The fact that a person can exhibit opposite patterns of judgments after observing identical patterns of outcomes is a fascinating characteristic of people's behavior when forecasting events in binary sequences. The primary challenge for any theoretical analysis of this phenomenon is to provide a valid explanation for the differences in predictions following identical patterns produced by different generators. A second challenge is to account for differences when people hold strong prior beliefs that the generator produces outcomes at a stationary, known rate, versus when people's prior beliefs about the generator's base rate are uncertain or ambiguous.

One class of explanations tries to meet both of these challenges by focusing on the observer's beliefs about the causal process that generates outcomes. Some accounts within this class characterize the beliefs as biased, reflecting some flawed reasoning process. Other accounts characterize the beliefs as the result of a reasonable belief‐updating process that reflects the true statistical properties of the observer's environment. Let us review each of these characterizations in the context of relevant empirical evidence.

### 1.1 Mental Models of the Generators: Random Devices, Intentional Actors, and Markets

#### 1.1.1 Random Mechanical Devices

People seem to hold incorrect beliefs about the causal operations of random mechanical devices. Nickerson (2002; also see Lecoutre, 1992) suggests that people assume random generators produce each outcome with equal probability, even when given little prior information about base rates (Blinder & Oppenheimer, 2008, provide experimental evidence). People also expect random generators to produce sequences with a high proportion of reversals, or alternation rates of about .60 (Ayton, Hunt, & Wright, 1989; Bar‐Hillel & Wagenaar, 1991; Falk, 1981; Rapoport & Budescu, 1997; Reimers, Donkin, & Le Pelley, 2018).^1^ When asked to judge sequences of outcomes that have unequal base rates, or that exhibit an alternation rate lower than .60, people view those sequences as too “streaky,” and judge the outcome‐generating process to be non‐random (Gronchi & Sloman, 2008; Lopes & Oden, 1987; Olivola & Oppenheimer, 2008; Scholl & Greifeneder, 2011). When asked to predict future outcomes for sequences produced by random generators, people also expect the proportion of outcomes to reflect the population base rate for each outcome type (i.e. to “balance out”) even in short sequences. This results in a gambler's fallacy pattern of increasing expectations that streaks of identical outcomes will reverse (cf. Boynton, 2003; Gronchi & Sloman, 2008; Studer, Limbrick‐Oldfield, & Clark, 2015).

#### 1.1.2 Intentional Actors

When asked to predict future outcomes for sequences produced by intentional actors, people exhibit a hot hand pattern of increasing expectations that streaks of identical outcomes will repeat (Alter & Oppenheimer, 2006; Bar‐Eli, Avugos, & Raab, 2006; cf. Boynton, 2003; Gilovich, Vallone, & Tversky, 1985; Fischer & Savranevski, 2015; Vergin, 2001). Some researchers suggest observers believe there is something special about *human* performance (Ayton & Fischer, 2004), or about the *intentional mind* of human actors (Caruso, Waytz, & Epley, 2010; Roney & Trick, 2009). But, people only exhibit hot hand beliefs for human actors whose performances they perceive as *non*‐random (Burns & Corpus, 2004; Tyszka, Zielonka, Dacey, & Sawicki, 2008).^2^

#### 1.1.3 Markets

People apparently hold a mixed bag of beliefs about markets. There is evidence that both novices (De Bondt, 1993) and experts (Baquero & Verbeek, 2015; Barberis, Shleifer, & Vishny, 1998; Shanthikumar, 2012) expect streaks of market outcomes (e.g. individual stock price movements) to repeat. There is also evidence that both novices (Anderson & Sunder, 1995) and experts (De Bondt, 1991; De Bondt & Thaler, 1990; Durham, Hertzel, & Martin, 2005; Loh & Warachka, 2012) expect streaks of market outcomes to reverse. People's predictions for streaks of market outcomes are also influenced by the alternation rate of prior outcomes (Bloomfield and Hales, 2002). And, not all market outcomes are perceived as equivalent. Novice and expert investors seem to have different mental models for small companies versus large, and for young companies versus old (Bulkley & Harris, 1997; Burns, 2003).

### 1.2 Theoretical Accounts of Prediction Behavior

#### 1.2.1 Heuristics and Biases

The heuristics and biases research program produced two related concepts that are often evoked to explain people's judgment behavior when forecasting outcomes in sequences of binary events. The *representativeness heuristic* proposes that people judge the likelihood of an outcome based on how well that outcome represents outputs from a mental model of the process the observer is trying to predict (Kahneman & Tversky, 1973; Tversky & Kahneman, 1983). The Law of Small Numbers principle describes an observer's expectation that small samples of outcomes will reflect the (statistical) parameters of the population from which they were drawn (Tversky & Kahneman, 1971). For example, people expect a small sample of coin flips to have an equal number of Heads and Tails outcomes, because they believe that the rate at which Heads and Tails are produced in the population of fair coin flips is *p*(Heads) = *p*(Tails) = .50. They expect the population rate (.50) to be expressed even in a very small sample of outcomes. Accordingly, an observer asked to predict the next outcome following a sequence containing a *dis*proportionate number of Heads (e.g. HTHHH) will assign higher probability to Tails, because that would make the resulting sequence (HTHHHT) more *representative* of the .50 rate in her mental model of random devices like coins.

The basic Law of Small Numbers principle only accounts for the gambler's fallacy pattern in people's predictions. Some extension is required to also account for the hot hand pattern in predictions for non‐random generators, like intentional (human) actors. Gilovich, Valone, and Tversky (1985) proposed that when observers see a sequence that does *not* exhibit a high rate of alternation, they decide the generator (e.g., a professional athlete) must be a non‐random mechanism, and shift to naïve expectations that outcomes will repeat at a high rate. We have several reservations about this interpretation, and with the data analysis the authors cite as support for this interpretation (recently noted by Miller & Sanjurjo, 2018b).

Rabin (2002; Rabin & Vayanos, 2010) offers an alternative Law of Small Numbers account that makes more sense to us. In Rabin's account, rational belief updating is the central cognitive process underlying predictions of events in binary sequences, but the observer has a non‐standard mental model of the generator's causal process. The observer begins with correct prior beliefs about the distribution of possible base rates and is rationally Bayesian. But, instead of assuming that outcomes are generated by an abstract *independent* and identically distributed (i.i.d.) random process, Rabin proposes that the observer assumes outcomes are sampled *without replacement* from a source with a finite number of potential outcomes (Estes, 1964; Fiorina, 1971; Morrison & Ordeshook, 1975; and Restle, 1961, also proposed *sampling without replacement* mental models).

In Rabin's model, the observer imagines outcomes are drawn *without replacement* from a small urn containing *N* signals, *s_i_* ∈ {*a*, *b*}, in proportion to the generator's base rate, *θ*. This means that the observer expects the urn to contain exactly *θN a* signals, and (1 – *θ*)*N b* signals. When the observer sees a short sequence of signals from a generator with a *known*, *stationary* rate, she reasons about them in terms of an urn whose contents are depleted as the sample of signals is drawn. Following repeated draws of one signal type, the observer believes there are *fewer* of that signal type left in the urn. As a result, she assigns increasingly lower probability to subsequent draws of that type, producing a gambler's fallacy judgment pattern.

In the case of a generator with a known, stationary rate, the observer expects the urn to contain each signal type in proportion to that rate, regardless of the outcomes she observes. However, when the observer is confronted with a generator having an *uncertain* or *ambiguous* rate, she adjusts her beliefs about the base rate (the proportion of each signal type in the urn) according to the outcomes she observes. In other words, the observer starts with the belief that the generator is like a random mechanical device with a base rate of .50. But, if she encounters a streak of identical signals, she overreacts to what she perceives as too few reversals. Conditional on the sequence of signals she observes, she updates her beliefs to reflect the most likely base rate to have produced that sequence, again in a Bayesian manner (Rabin & Vayanos, 2010, discussion starting on p. 746).^3^ If a given signal type continues to repeat, increasing the length of the streak, the observer continues to update her beliefs, assigning an even higher rate to the signal type repeated in that streak. Thus, as streak length increases, the observer's predictions will shift from a gambler's fallacy pattern to a hot hand pattern.

A Bayesian updating process that produces a shift from initially expecting reversal to eventually expecting repetition is unique to Rabin's Small Urn Model.^4^ A few empirical reports provide suggestive evidence that people's predictions for binary sequences do exhibit a U‐shaped pattern (especially for long streaks of more than 10 binary outcomes; Altmann & Burns, 2005; Asparouhova, Hertzel, & Lemmon, 2009; Edwards, 1961; Lindman & Edwards, 1961; Jarvik, 1951; Nicks 1959; Rao, 2009; Suetens, Galbo‐Jorgensen, & Tyran, 2016; Tyszka, Markiewicz, Kubińska, Gawryluk, & Zielonka, 2017; and see early laboratory studies summarized in Lee, 1971, pp. 163–167).

With reference to the apparent prevalence of hot hand beliefs among observers judging sequences produced by intentional actors, Rabin suggests that people have weak prior beliefs about the base rate at which an intentional actor produces different types of outcomes, and therefore people engage in belief updating about the base rate early in their prediction strategy.

Rabin's model is consistent with the representativeness heuristic and the Law of Small Numbers principles. People assign higher probabilities to *reversal* of streaks produced by random mechanical devices, because high reversal rates are representative of their mental model of random mechanical devices. People assign higher probabilities to *repetition* of streaks in human behavior, because they think that pattern is more representative of their mental model of intentional human actors. People exhibit mixed judgment patterns in their predictions for markets, because they hold heterogeneous beliefs about how markets work. Even experts can't agree about the presence or absence of patterns in market data.

To us, both of these explanations provide *post hoc* descriptions of the patterns researchers observe in their experimental and observational data rather than *ex ante* predictions of behavior. The representativeness heuristic and the Law of Small Numbers do not specify parameters of people's mental models *a priori*, or tell us much about their origin (cognitive, environmental, or otherwise).^5^ Accounts of these heuristics also do not provide definite predictions when people are faced with a new type of generator for which there are no previously accumulated judgment data (or in cases where contradictory patterns are observed in those data, as is the case for markets).

#### 1.2.2 Experience and Education

Two additional accounts also start with the assumption that judgment habits are adaptive and essentially rational. One explanation for the origin of people's expectations across different generators is an ecological account that posits people's beliefs result from a learning process that reflects the true statistical properties of their environment. Hahn & Warren (2009) present a version of this account (also Farmer, Warren, & Hahn, 2017; and related explanations are developed by Kareev, 2001; Miller & Sanjurjo, 2018a; Reimers, Donkin, & Le Pelley, 2018; and Sun & Wang, 2010). The authors remind us that people have limited attention and finite experience, and usually observe short sequences of events in experiential episodes (e.g., a basketball game, an evening playing roulette). While a theoretical i.i.d. Bernoulli process will produce an *infinite* sequence of outcomes in which all exact orderings of substrings (e.g., HHHH, HHTT) are equally likely, the same is not true for *finite* samples of sequences. Given finite samples, the probability of observing a given substring depends on the number of different realizations of the sample in which that substring occurs. To put it another way, imagine an observer watching sequential tosses of a fair coin. It will usually take longer for this observer to encounter the substring HHHH (about 30 tosses) than to encounter HHTT (about 16 tosses).^6^ If it is known *ex ante* that the observer will only watch 20 tosses total, then it is more likely the observer will encounter HHTT than HHHH within that sample of 20 tosses.

Reversal expectations for random generators also result from popular mathematics pedagogies used to teach probability and statistics in classrooms. The methods used to teach concepts related to probability and randomness reinforce reliance on the representativeness heuristic when judging sequences produced by random mechanical devices (Amir & Williams, 1999; Batanero, Chernoff, Engel, Lee, & Sánchez, 2016; Borovnik & Peard, 1996; Harradine, Batanero, & Rossman, 2011; Hawkins & Kapadia, 1984; Jones, 2004; Konold, 1995; Meletiou‐Mavrotheris, 2007; Morsanyi, Handley, & Serpell, 2013; Shaughnessy, 1992; Shaughnessy, Canada, & Ciancetta, 2003; Steinbring, 1990).^7^

Whether everyday experience or formal education leads people to build an assumption of negative serial correlation into their mental models of random generators, it does not seem as though reversal predictions are innate or automatic. The most common prediction pattern for very young children is to expect repetition of streaks (hot hand). The gambler's fallacy starts to show up in elementary school, and peaks among college students (Bogartz, 1965; Chiesi & Primi, 2009; Derks & Paclisanu, 1967; Estes, 1962; Craig & Meyers, 1963). People also take longer to predict reversal than repetition, and are more likely to predict repetition than reversal under time constraints or cognitive load (Braga, Ferreira, Sherman, Mata, Jacinto, & Ferreira, 2018; Diener & Thompson, 1985; Militana, Wolfson, & Cleaveland, 2010; Tyszka, Markiewicz, Kubińska, Gawryluk, & Zielonka, 2017).

People's mental models of intentional actors do reflect the true behavior of these generators. Gilovich and colleagues' (1985) seminal paper generated a lot of buzz for claiming that basketball fans’ belief in the hot hand was inaccurate. A flurry of empirical studies followed, some confirming these claims by apparently demonstrating that serial correlation in sequences of human performance did not exceed chance levels, and others counter‐arguing by demonstrating consistent, significant positive recency in sequences of skilled human performance (Alter & Oppenheimer, 2006; Bar‐Eli, Avugos, & Raab, 2006). Recently, Miller and Sanjurjo (2018b) identified an error in Gilovich and colleagues’ original analysis of basketball shooting data, as well as in several replications of those results. Miller and Sanjurjo's re‐analysis of these studies revealed, “significant evidence of streak shooting, with large effect sizes,” (*ibid*, p. 2022) in the original basketball data, as well as “hot hand effect sizes [that] are consistently moderate to large,” (*ibid*) in the data from replications by Avugos, Bar‐Eli, Ritov, and Sher (2013a), and Koehler and Conley (2003).

It is difficult to identify a “ground truth” for the behavior of financial market generators. There's evidence of both positive and negative serial autocorrelation in stock market outcomes (Conrad & Kaul, 1998; De Bondt & Thaler, 1989; Jegadeesh & Titman, 2011). Both momentum (betting on repetition) and contrarian (betting on reversal) investment strategies yield statistically significant profits in some market segments (Conrad & Kaul, 1998). So, it's difficult to say that either hot hand or gambler's fallacy predictions for stock market outcomes are unreasonable.

We find the ecological accounts of people's prediction behavior compelling (e.g. Hahn & Warren, 2009), but these accounts suffer from the same limitation as the heuristics and biases approach: It's not clear *a priori* which “real world” events are sources of influence on current predictions for different sequences. For example, how can we anticipate people's prior beliefs about the field goal success rate of a basketball player? Should we focus on the success rate for field goal shots taken by all athletes encountered over the observer's lifetime of experience with basketball, or only those taken by players on the observer's favorite team, or those taken only by his single favorite player? Or, might the observer be relying on experiences across multiple sports and other goal‐directed achievement activities? Without knowing which experiences people might draw upon to form their mental model of a given generator, we cannot derive hypotheses about their judgments of that generator.

In conclusion, our favored hypothesis about when to expect observers will predict repetitions versus reversals is based on Rabin's Small Urn Model. Rabin proposed gambler's fallacy (reversal) predictions will arise when people believe the generator has a *known*, *stationary* base rate, but that hot hand (repetition) predictions will arise when people are *uncertain* about the base rate of the generator (or when they believe that rate may change over time). We like this interpretation because it provides the most principled explanation for differences in participants’ prediction patterns for different types of generators. The major conceptual problem that remains is that we don't know *ex ante* whether people hold strong versus weak prior beliefs over the base rate of a given generator, especially an unfamiliar one.

### 1.3 Methodological Challenges in The Extant Literature

Most experimental studies investigating predictions of binary events in sequences present slightly different information about random versus intentional generators. Instead of clearly specifying identical base rates for each generator, experimenters either rely on participants’ prior beliefs (e.g., that a fair coin has a stationary .50 base rate, or that a basketball player performs at an unspecified, perhaps “typical,” rate), or they provide base rate information that may be interpreted as *stationary* for random devices, but *shifting* for intentional actors. For example, Braga and colleagues (2018) compared predictions for coin flips to those for athletic performances. The authors provided explicit information that the coin was *fair* (stationary .50 base rate), and that flipping the coin was a random process. Their description of the athletes provided no information about the athletes’ performance rates, and explicitly stated that these rates shift as the athlete ages. Burns and Corpus (2004) compared predictions for a roulette wheel to those for a competitive car salesman and a little sister shooting baskets. Participants were informed that each generator had produced each type of outcome on 50/100 past trials. But it was not clear whether this .50 rate was stationary or shifting, and both the car salesman and little sister were described as attempting to improve their performances over time.

If experimenters consistently present clear, concrete information for random mechanical devices, but not for intentional actors, then we should expect differences in participants’ judgment patterns for these types of generators.^8^ In each of the present studies, we provide participants with explicit, identical information about the base rate for all three of the generators (random mechanical device, intentional actor, and market process). Across studies, we systematically manipulate participants’ level of certainty about the generators’ base rates. This method provides a direct test of Rabin's conjecture that uncertainty about the base rate determines whether people exhibit hot hand or gambler's fallacy patterns in their predictions for sequences of binary events.

## 2 Overview of the Present Experiments

Participants were shown 18 sequences of 8 binary outcomes, and asked to predict the direction of the 9th (next) outcome in each sequence. The structure of the task makes it straightforward to study the way information from bottom‐up data and top‐down abstractions interact to produce a unitary response.

Participants were assigned to judge sequences produced by one of three different generators: (1) a bingo cage filled with red and blue balls (random mechanical device, Red or Blue outcomes); (2) an investment analyst whose portfolio increases or decreases in value (intentional, goal‐directed actor, Up or Down outcomes); and (3) a publicly traded company whose stock increases or decreases in price (market process, Up or Down outcomes). Each participant judged six Target experimental sequences, each ending in one of the following Streak Lengths: 2, 3, 4, 5, 6, and 7.^9^ According to the representativeness heuristic account, participants judging bingo cage sequences should exhibit a preference for reversal of streaks, and participants judging the investment analyst should exhibit a preference for repetition. (We do not have a strong representativeness account prediction for stock prices, due to the mixed results and interpretations in past studies.) An uncertainty‐dependent account (Rabin's Urn Model) predicts identical patterns of preferences across generator types as long as base rate information is held constant.

Participants are asked to predict the next (9th) outcome in each sequence either by making a dichotomous choice, or making a numerical probability rating. To date, there is no study that directly compares judgments across these two response formats, and elicitation formats vary unsystematically across studies in the extant literature. We include both formats to facilitate comparison between our results and those in preceding studies. There is also some evidence that response format influences the likelihood that people will engage in intuitive versus analytical reasoning styles. Intuitive reasoning seems to occur more often when people are faced with a dichotomous choice, and analytical reasoning more often when people report numerical probabilities.^10^ Further, we conjecture that responding on a numerical scale reminds the participant of the fixed numerical base rate, if one was specified, increasing the tendency to respond with that value. If gambler's fallacy and hot hand patterns result from reliance on intuitive (heuristic) reasoning, we should see more reversal predictions for the bingo cage (mechanical device) and more repetition predictions for the investment analyst (intentional actor) among participants asked to make a dichotomous choice than among those asked to provide numerical probability ratings.

Within each Study, we provided identical information about the base rates of the three generators.^11^ In Study 1, we provided no information about any of the generators’ base rates. We do not indicate the ratio of red to blue balls in the bingo cage, the rate at which the investment analysts’ portfolios increase or decrease in value, or the rate at which the companies’ stock prices increase or decrease. We anticipated participants’ expectation of repetition would *increase* with the length of the terminal streak at the end of each Target experimental sequence, as they update their beliefs about the base rate of the generator (Rabin, 2002; cf. Burns, 2002). In Study 2, we provided a stationary base rate of .50 for all three generators. We hypothesized that participants will *decrease* their expectation of repetition across the experimental sequences with shorter terminal streaks, and then *increase* judgments of repetition as streak length increases and the sequence seems less likely to have been produced by the stated base rate (Rabin, 2002). In Study 3, we specified the same distribution of possible base rates for all three generators. The precise specification of the prior distribution allows us to calculate a Bayesian updated posterior distribution. We expected participants to approximate the Bayesian updating pattern of increasing expectations of repetition as streak lengths increased.

### 2.1 Participant Recruitment

Participants in the present studies were sampled from Amazon Mechanical Turk, and were required to live in the United States and have a Human Intelligence Task (HIT) approval rate of at least 95% over at least 5 previously completed HITs (Mason & Suri, 2012).^12^ No participant took part more than once in any Study, nor did any participants take part in more than one of our Studies. No participant who completed the full procedure was excluded from our analyses.

### 2.2 Procedure

The procedure was implemented using the oTree platform (Chen, Schonger, & Wickens, 2016). Participants first read instructions corresponding to their assigned Condition (defined by the qualitative description of the generator). Participants faced with the Analyst generator were told that they would see quarterly changes in the value of investment portfolios managed by different stock analysts. They were told that stock analysts look for trends in the stock market, that they use this information to invest their clients' money wisely, and that the analysts' decisions determine whether the total value of their portfolios increase or decrease each quarter. Participants faced with the Stock generator were told that they would see quarterly changes in the price of different public companies' stocks. They were told that the price movement of a stock reflects the market's evaluation, and buyers' and sellers' expectations of a company's worth. They were also told that many factors influence stock prices, such as earnings reports, news about a company's leadership and products, economic policies, and political events. Participants faced with the Bingo generator were told that they would see draws made by a mechanical bingo machine from a covered cage containing red and blue balls.^13^ They were also told that each time a ball was drawn from the cage, it was replaced before the next draw was made.

After reading the instructions, participants were required to answer 4–5 comprehension questions correctly before beginning the experimental task.^14^ (Special care was taken to verify that all participants judging sequences from the bingo cage understood that the outcomes in these sequences were sampled *with replacement*.) Participants viewed one 8‐outcome sequence on each of 18 trials. The 6 Target sequences, each ending in a streak of between 2 and 7 identical outcomes, were mixed with 12 Filler sequences, each ending in a reversal (e.g. Red‐Blue, Down‐Up). Filler sequences balanced the frequency of streaks and proportion of signal types across trials of the experiment.^15^

On each trial, participants were instructed that they were observing a new sequence of 8 consecutive outcomes, not a continuation of the outcomes observed on previous trials. Each outcome was revealed one at a time, and remained visible on the screen for the duration of the trial. There was a one‐second delay between the appearance of each outcome.^16^ Participants were then asked to predict the next (9th) outcome by making a dichotomous choice or by selecting a numerical rating on a continuous probability scale (0% to 100%).^17^ No feedback was provided after the participants made each prediction. After completing the experimental procedure, participants answered 5 questions testing their knowledge of probability and financial literacy. Participants were also asked to report demographic information (age, gender, and highest degree),^18^ and to describe the strategy they used to make their predictions.^19^

## 3 Studies 1A and 1B

Our goal in Studies 1A and 1B was to study how people make judgments given verbal descriptions of our three generators (bingo cage, stock analyst, and public company) without any information about the generators’ base rates.

### 3.1 Participants

In Study 1A, 144 participants (M_AGE_ = 36.02, SD_AGE_ = 11.23, N_FEMALE_ = 58) took 18.69 minutes on average (SD = 10.02) to complete the procedure. In Study 1B, 300 participants (M_AGE_ = 35.43, SD_AGE_ = 11.79, N_FEMALE_ = 143) took 17.75 minutes on average (SD = 9.65) to complete the procedure. Participants were paid $2.50 upon approval of their completed tasks.^20^

### 3.2 Method

Participants in Study 1A made predictions on a continuous probability scale; in Study 1B they made a dichotomous choice. Participants were randomly assigned to one of three experimental conditions. In the BingoUnknown Condition, the events in each sequence were described as draws made *with replacement* by a mechanical bingo machine from a cage containing 100 red and blue balls. No information was provided about the ratio of red to blue balls. In the AnalystUnknown Condition, the events in each sequence were described as quarterly changes (Up/Down) in the value of one stock analyst's portfolio. No information was provided about the rate at which the analyst's portfolio increased or decreased in value. In the StockUnknown Condition, the events in each sequence were described as quarterly changes (Up/Down) in a single company's stock price. No information was provided about the rate at which the company's stock price increased or decreased. On each trial, participants were instructed that they were viewing a *new* sequence to emphasize that they were not seeing a continuation of the previous trial's sequence.

### 3.3 Results: Study 1A

**Fig. 1:**
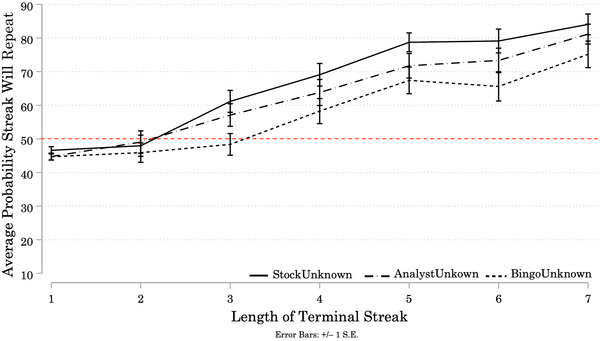
Study 1A: Average Probability Participants Assigned to Repetition of Terminal Streaks, by Streak Length and Condition *Note*. *N* = 144 (StockUnknown = 44; AnalystUnknown = 50; BingoUnknown = 50). Solid and patterned lines represent the average probability assigned by participants in each Condition to the event that the next (9th) outcome will repeat the streak of identical signals they observed at the end of each Target sequence. In this and subsequent figures, we also include participants' predictions for Filler sequences ending in a streak of length 1 to facilitate visual comparison between our results and those in the extant literature. Note that statistical analyses exclude Filler sequences (but see Chapter 3 of the Supplemental Material for more information about participants' responses to the Filler sequences). Error bars represent +/– 1 standard error. Starting at Streak Length 3, participants in all three Conditions assigned greater than 50% probability to the event that the next signal would repeat the streak, and these probabilities increased with Streak Length.

Figure 1 shows the average probability participants assigned to *repetition* of the streak at the end of each Target sequence. On average, participants in all three Conditions assigned greater than 50% probability to repetition of the terminal streaks and expectations of repetition increased with Streak Length. We conducted a one‐way mixed ANOVA to test the effects of one between‐subjects variable (Condition), and one within‐subjects variable (Streak Length) on ratings of the probability that the terminal streak would repeat (see Appendix A: Average Results, Table A1 for numerical results).^21^

There was a significant effect of Condition on participant predictions (*F*(2, 141) = 4.11, *p* = 0.018). Bonferroni‐corrected pairwise comparisons revealed that predictions made by participants in the BingoUnknown Condition were significantly lower than predictions made by participants in the StockUnknown Condition (Mean Difference = –9.90, *p* = 0.016). However, predictions made by participants in the AnalystUnknown Condition were *not* significantly different than predictions made by participants in the BingoUnknown Condition (Mean Difference = 5.92, *p* = 0.247), *or* by participants in the StockUnknown Condition (Mean Difference = –3.98, *p* = 0.770).

Longer streaks were assigned higher probabilities of repeating, (*F*(4.43, 623.98) = 58.46, *p* < 0.001).^22^ This finding suggests that participants are updating their estimates of the base rate as Streak Length increases, consistent with Rabin's (2002) model that predicts expectations of repetition will increase over longer streaks of identical signals when the base rate is ambiguous. There was no significant interaction between Streak Length and Condition (*F*(8.85, 623.98) = 0.64, *p* = 0.758). Participants faced with a generator described as an intentional actor updated their beliefs in a similar fashion to those faced with a generator described as a random mechanical device or as a market.

To understand participants’ individual prediction strategies, we look at the slopes of linear regressions fitted to each participant's predictions over the 6 target sequences. There was some heterogeneity in individual participants’ prediction strategies, as measured by the slopes of their predictions across Streak Lengths (see Appendix B for a graphical representation).^23^ On average, each incremental increase in the length of the terminal streak corresponds to an increase of about 5% in the probability participants assigned to repetition of that streak. The dominant strategy in all three Conditions is a positive slope, which again implies participants are updating their beliefs about the base rates of the generators as Streak Length increases (see Appendix B for a graphical summary). A minority of participants decreased their expectations of repetition as Streak Length increased, consistent with gambler's fallacy reasoning: 14% in the AnalystUnknown Condition, 7% in the StockUnknown Condition, and 20% in the BingoUnknown Condition.

### 3.4 Results: Study 1B

**Fig. 2:**
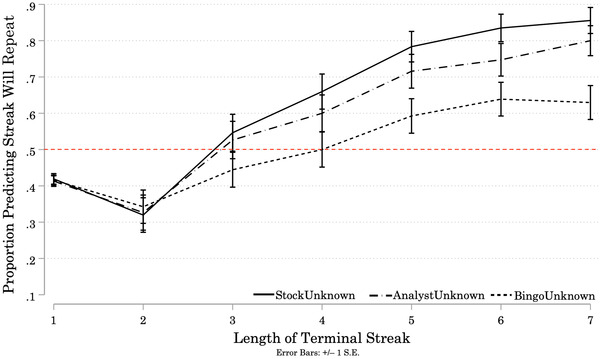
Study 1B: Proportion of Participants Predicting Repetition of Terminal Streaks, by Streak Length and Condition *Note*: Results of Study 1B. N = 300 (StockUnknown = 97; AnalystUnknown = 95; BingoUnknown = 108). Solid and patterned lines represent the proportion of participants in each Condition who predicted the next (9th) outcome will repeat the terminal streak at the end of each Target sequence. Error bars represent +/– 1 standard error. Starting at streaks of length 4, more than 50% of participants in the StockUnknown and AnalystUnknown Conditions predicted that the next signal would repeat the streak. At streaks of length 5 and greater, more than 50% of participants in the BingoUnknown Condition predicted the next signal would repeat the streak.

The results of Study 1B (Figure 2) reflect the results of Study 1A. A one‐way mixed ANOVA was conducted to test the effects of Condition and Streak Length on participants’ predictions that the streak at the end of each Target sequence would repeat (1) or reverse (0).^24^ There was a significant effect of Condition on participant predictions (*F*(2, 297) = 5.62, *p* = 0.004). Bonferroni‐corrected pairwise comparisons revealed a significantly smaller proportion of participants in the BingoUnknown Condition predicted streaks would repeat than in the StockUnknown Condition (Mean Difference = –0.14, *p* = 0.004). There were no significant differences between the proportions of participants predicting streaks would repeat in the AnalystUnknown and BingoUnknown Conditions (Mean Difference = 0.10, *p* = 0.092), or between the proportions in the AnalystUnknown and StockUnknown Condition (Mean Difference = –0.05, *p* = 0.872). There was a significant main effect of Streak Length on predictions, (*F*(4.52, 1343.13) = 57.74, *p* < 0.001).^25^ A higher proportion of participants predicted streaks would repeat as Streak Length increased. Participants responded to increases in Streak Length similarly across all three Conditions (Streak Length × Condition interaction: *F*(9.05, 1343.13) = 1.61, *p* = 0.107).

As in Study 1A, there was heterogeneity across individual participants’ prediction strategies (see Appendix B). We fitted a logistic regression to each participant's predictions over the Target sequences ending in streaks of length 2–7 to obtain the log odds coefficient for the effect of Streak Length on the probability that the participant predicts “repeat.”^26^ We then transformed the log odds coefficient into a percent‐change in the odds the participant predicts “repeat” for each unit increase in Streak Length.^27^ A positive percent‐change in the odds indicates the participant is more likely to predict repetition as Streak Length increases. A negative percent‐change in the odds indicates the opposite strategy. The distributions of percent‐changes are centered at or above 50% in all three Conditions, indicating most participants' expectations of repetition increased with Streak Length.^28^ A minority of participants exhibited a preference for reversal (negative percent‐change in the odds): 8% in the AnalystUnknown Condition, 10% in the StockUnknown Condition, and 16% in the BingoUnknown Condition.

### 3.5 Discussion: Studies 1A and 1B

When given no information about the generators' base rates, the majority of participants in Studies 1A and 1B increased their expectations that a streak would repeat as the length of that streak increased (hot hand pattern). A small number of participants in each study (8% to 16%) decreased their expectations that a streak would repeat as the length of that streak increased (gambler's fallacy). These findings support Rabin's and Burns's proposals that participants update their beliefs about the generator's base rate as Streak Length increases. The ordering of participants’ predictions does not suggest that participants are especially prone to believe in a hot hand process when observing the behavior of an intentional actor.

We interpret the shallower, more conservative numerical updating curve for the mechanical bingo cage generator as consistent with Rabin's hypothesis that conceptions of random mechanical devices are more strongly anchored on prior beliefs than those of intentional actors (and social market processes). More specifically, we speculate that the fixed, rigid mechanical process does not allow observers to imagine shifting performance states (according to the instructions, the cage had fixed contents and was sampled with replacement), compared to processes that include a human component that might shift motivational or learning states over time (e.g., from slacking to striving).

Every generator shows an early increase in reversal predictions (for streaks of lengths 1 and 2) on the dichotomous outcome response format. (This reversal judgment pattern is dramatic for streaks of 1 and 2, for all generating mechanisms, in all three dichotomous response scale experiments.). We interpret this as evidence for an outcome‐depletion component of participants’ mental models for all generators (consistent with Rabin and others’ notion of sampling without replacement from a small urn of outcomes). We highlight this small effect here because it will be observed in every experiment in this report in which the dichotomous response format is employed, and it is the most visible difference between responses on the continuous probability scale versus on the dichotomous choice format.

In order to compare the responses of participants in Studies 1A and 1B, participants’ responses in Study 1A were dichotomized. Predictions higher than 50% were coded as “1” (streak will repeat), and predictions lower than 50% were coded as “0” (streak will reverse).^29^ Obviously, this requires an assumption that proportions of responses across participants can be interpreted as reflecting individual strengths of belief, and some readers may want to disregard our comparative analyses.

We performed Welch's t‐test for unequal variances to compare the proportion of participants predicting streaks would repeat in Study 1A to the proportion of participants predicting streaks would repeat in Study 1B.^30^ A significantly smaller proportion of participants in each Condition of Study 1B predicted streaks would repeat than in each corresponding Condition of Study 1A (Bingo_STUDY1B_ – Bingo_STUDY1A_ = –0.14, *s.e*. = 0.03, *p* < 0.001; Analyst_STUDY1B_ – Analyst_STUDY1A_ = –0.11, *s.e*. = 0.03, *p* < 0.001; Stock_STUDY1B_ – Stock_STUDY1A_ = –0.13, *s.e*. = 0.03, *p* < 0.001). Participants were not more likely to exhibit a hot hand pattern of increasing beliefs in repetition when responding with a dichotomous choice than on a continuous probability scale.

## 4 Studies 2A and 2B

In Studies 2A and 2B, we provided explicit instructions that each generator produces each type of outcome at a fixed base rate of .50. We wanted to know whether people respond to stationary base rate information differently depending on the generator; specifically, whether the fixed base rate bingo cage generator would elicit higher rates of reversal predictions as implied by gambler's fallacy reasoning.

### 4.1 Participants

One hundred and fifty‐six participants (M_AGE_ = 35.57, SD_AGE_ = 10.25, N_FEMALE_ = 69) took 18.15 minutes on average (SD = 9.67) to complete Study 2A. Three hundred and one participants (M_AGE_ = 35.58, SD_AGE_ = 12.51, N_FEMALE_ = 141) took 18.57 minutes on average (SD = 9.47) to complete Study 2B. Participants in both studies were paid $2.50 upon approval of their completed tasks.

### 4.2 Method

The procedures for Studies 2A and 2B were identical to the procedures for Studies 1A and 1B, respectively, with the exception that base rates were explicitly provided and described as being fixed at .50.^31^ In the Bingo50 condition, the events were described as draws (Red/Blue) made *with replacement* from a cage containing exactly 50 red and 50 blue balls. In the Analyst50 Condition, participants were instructed that the analysts they judged had *average* skill levels, such that the value of their portfolios increased exactly 50% of the time, and decreased otherwise. In the Stock50 Condition, participants were instructed that the companies they judged had *average* performance levels, such that their stock prices increased exactly 50% of the time, and decreased otherwise.

### 4.3 Results: Study 2A

**Fig. 3:**
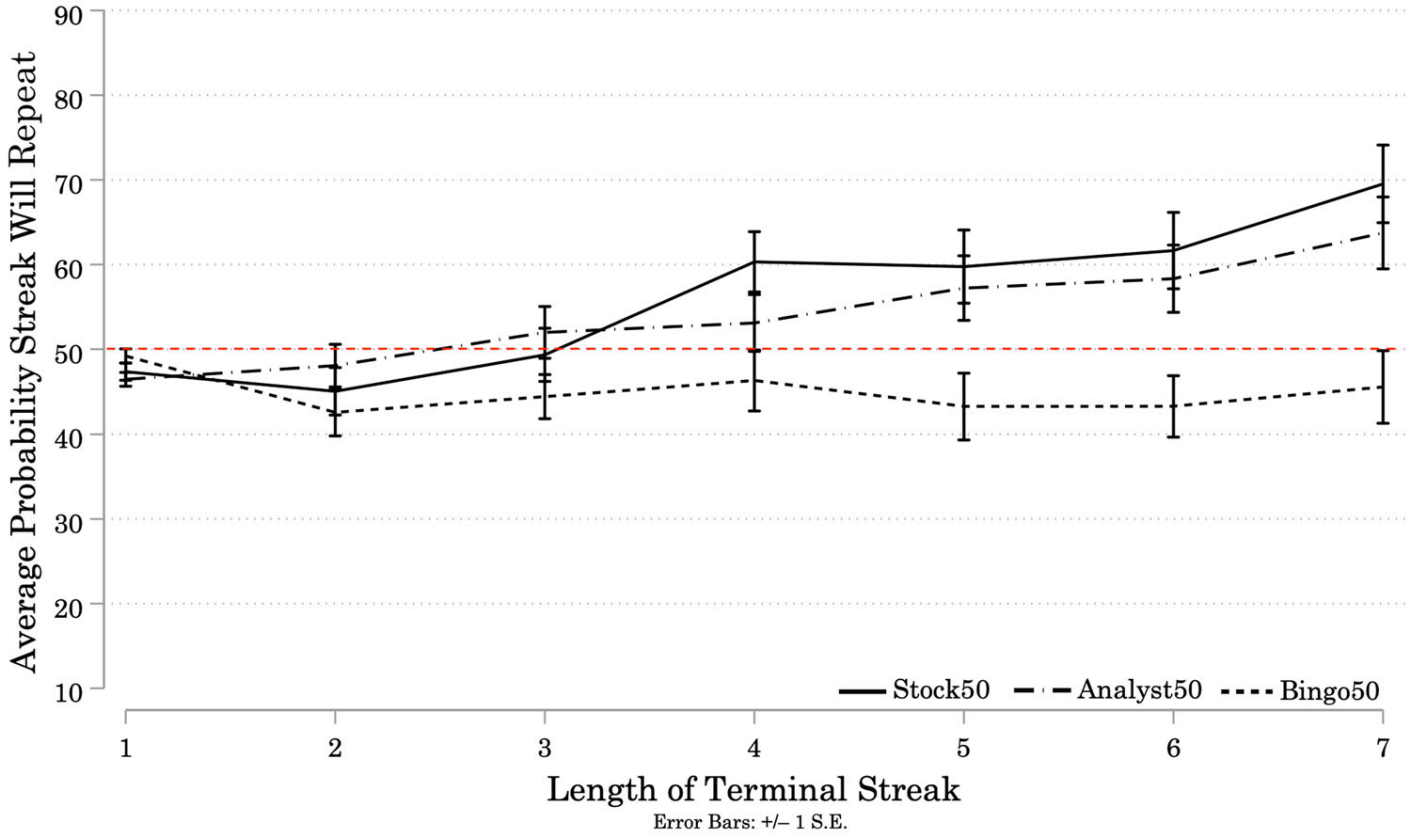
Study 2A: Average Probability Participants Assigned to Repetition of Terminal Streaks, by Streak Length and Condition *Note*: Results of Study 2A. N = 156 (Stock50 = 48; Analyst50 = 52; Bingo50 = 56). Solid and patterned lines represent the average probability participants assigned to repetition of the terminal streak at the end of each Target sequence. Error bars represent +/– 1 standard error. Starting at Streak Length 4, Analyst50 and Stock50 participants assigned greater than 50% probability to repetition of the streak, and increased their ratings with Streak Length. Bingo50 participants consistently assigned lower than 50% probability to repetition of the streak, and did not consistently increase or decrease their ratings as Streak Length increased.

Figure 3 presents the average probability participants assigned to repetition of the terminal streak at the end of each Target sequence. Predictions made by participants faced with an intentional actor (Analyst50 Condition) were *not* significantly different from predictions made by participants faced with a market process (Stock50 Condition). For streaks of length 2 or 3, participants made similar predictions across all three Conditions. Starting at streaks of length 4, participants in the Analyst50 and Stock50 Conditions assigned greater than 50% probability to repetition, and increased the probability as Streak Length increased. But, participants in the Bingo50 Condition assigned probabilities consistently lower than 50% across all Streak Lengths.

We conducted a one‐way mixed ANOVA to test the effects of one between‐subjects variable (Condition), and one within‐subjects variable (Streak Length) on ratings of the probability that the terminal streak would repeat.^32^ There was a significant effect of Condition on participant predictions (*F*(2, 153) = 7.46, *p* = 0.001).

Bonferroni‐corrected pairwise comparisons revealed that the mean predictions made by participants in the Bingo50 Condition were significantly lower than the mean predictions made by participants in the Analyst50 Condition (Mean Difference = −11.19, *p* = 0.009), and by participants in the Stock50 Condition (Mean Difference = −13.38, *p* = 0.002). There was no significant difference between the predictions made by participants in the Analyst50 and Stock50 Conditions (Mean Difference = −2.20, *p* = 1.001). Participants faced with a random mechanism (balls drawn from a bingo cage) were more likely to predict reversals across all Target sequences, and participants faced with an intentional actor (investment analyst) or a market process (publicly‐traded company) were more likely to predict repetition of terminal streaks of length 4 or greater.^33^

There was a significant main effect of Streak Length on predictions, (*F*(3.91, 598.01) = 10.07, *p* < 0.001).^34^ Longer Streak Lengths were assigned higher probabilities of continuing. There was a significant interaction between Streak Length and Condition (*F*(7.82, 598.01) = 2.56, *p* = 0.010). Bonferroni‐corrected pairwise comparisons revealed no significant differences between predictions made by participants in each of the three Conditions for streaks of length 2 or 3. For streaks of length 4, there was no significant difference between predictions made by participants in the Analyst50 and Bingo50 Conditions (Mean Difference = 6.79, *p* = 0.499), but Stock50 participants’ predictions were significantly higher than those of Bingo50 participants (Mean Difference = 14.01, *p* = 0.017). For streaks of length 5, 6, and 7, participants in the Analyst50 and Stock50 Conditions assigned significantly higher probabilities to repetition of the terminal streak than did participants in the Bingo50 Condition. Participants in the Analyst50 and Stock50 Conditions seemed to update their estimates of the generator's base rate as Streak Length increased, similar to what we observed in Study 1A. Predictions by participants in the Bingo50 Condition were consistently lower than 50% (and did not change with Streak Length).

To understand participants’ individual prediction strategies, we again look at the slopes of linear regressions fitted to each participant's predictions over the 6 Target sequences (see Appendix B). Individual participants employed similar, albeit more conservative, updating strategies in the Analyst50 and Stock50 Conditions (Study 2A) to what they did in the AnalystUnknown and StockUnknown Conditions (Study 1A). But, we do see a difference between the prediction strategies employed in the Bingo50 Condition and the BingoUnknown Condition.^35^ There were more extreme outliers in both the left‐ and right‐tails of the distribution in the Bingo50 Condition in Study 2A than there were in the BingoUnknown Condition of Study 1A. The predictions made by 54% of participants in the Bingo50 Condition in Study 2A exhibited a negative slope, compared to only 20% in the BingoUnknown Condition of Study 1A.

If we focus on the average results, we would conclude that participants given a stationary base rate for a random generator (Bingo50 Condition) show a bias to predict reversals, consistent with gambler's fallacy reasoning. Although the expectation of reversals appears constant across Streak Lengths at the aggregate level, our analysis of individual prediction slopes implies that a substantial number of participants exhibited an increasing tendency to predict reversals (54%). In spite of the explicit instructions about a stationary base rate, participants faced with a market process (Stock50 Condition) or an intentional agent (Analyst50 Condition) eventually updated their estimates of the base rate for longer streaks. (We interpret the fact that participants updated more conservatively in Study 2A than in Study 1A as an indication that their prior beliefs were to some extent anchored on the base rate in the instructions in 2A.)

### 4.4 Results: Study 2B

**Fig. 4:**
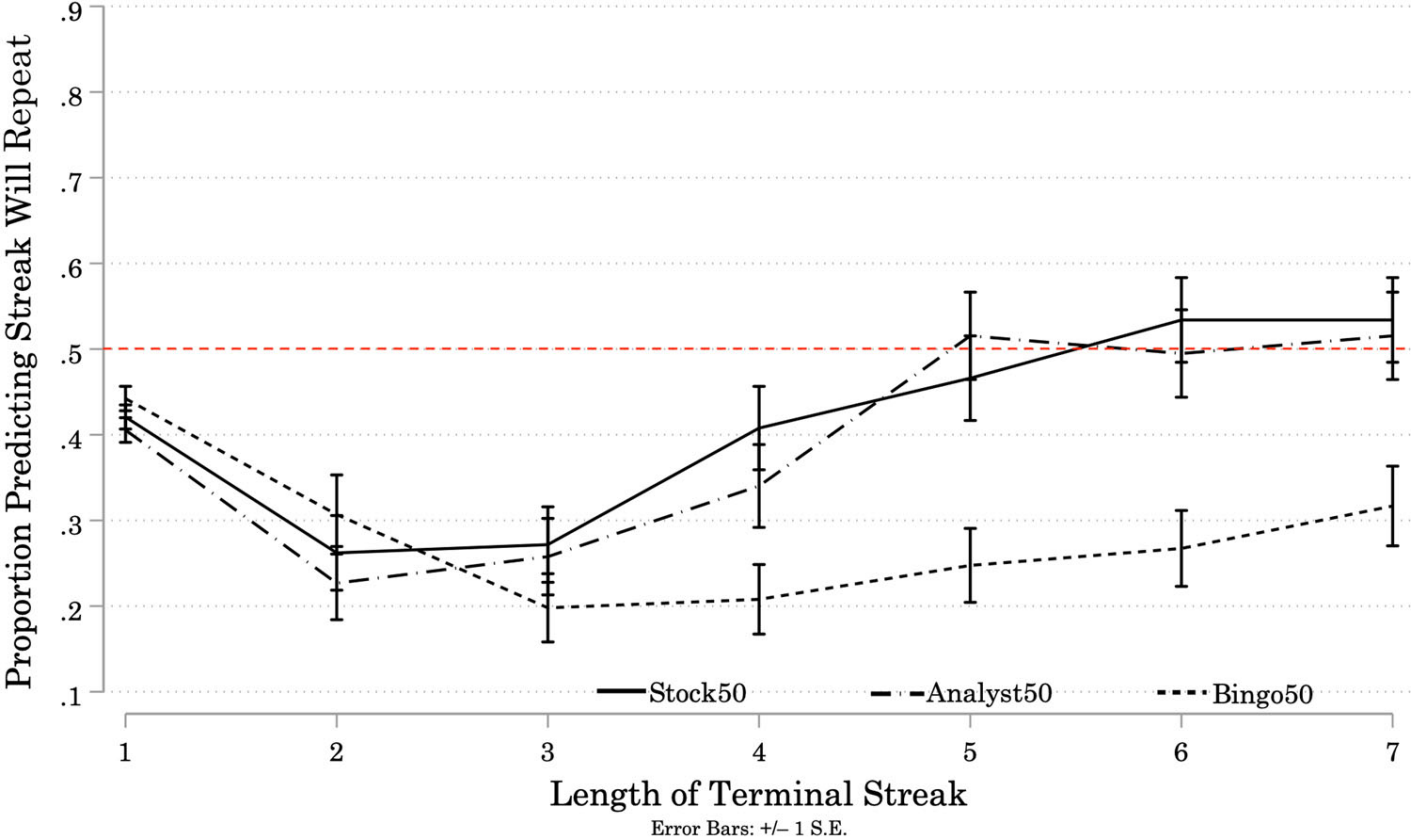
Study 2B: Proportion of Participants Predicting Repetition of Terminal Streaks, by Streak Length and Condition *Note*: Results of Study 2B. N = 301 (Stock50 = 103; Analyst50 = 97; Bingo50 = 101). Solid and patterned lines represent the proportion of participants in each Condition who predicted repetition of the terminal streak at the end of each Target sequence. Error bars represent +/– 1 standard error. Across streaks of length 2–4, fewer than 50% of participants in all three Conditions predicted that the streak would repeat. The proportion of participants predicting repetition in the Analyst50 and Stock50 Conditions increased across Streak Length 2–5, before leveling off at around 50%. In contrast, only 20% to 30% of participants in the Bingo50 Condition predicted streaks of any length would repeat.

Figure 4 shows the proportions of participants who predicted the streak at the end of each Target sequence would repeat. Across streaks of length 2–4, fewer than 50% of participants in all three Conditions predicted repetition. The proportion of participants predicting repetition in the Analyst50 and Stock50 Conditions increased across Streak Lengths 2–5, before leveling off at around 50%. In contrast, only 20% to 30% of participants in the Bingo50 Condition predicted repetition across all Streak Lengths.

We conducted a one‐way mixed ANOVA to test the effects of one between‐subjects variable (Condition), and one within‐subjects variable (Streak Length) on participants' predictions (“1” for *repeat*; “0” for *reverse*).^36^ There was a significant effect of Condition on participant predictions (*F*(2, 298) = 7.09, *p* = 0.001).^37^ Bonferroni‐corrected pairwise comparisons revealed a significantly smaller proportion of participants in the Bingo50 Condition predicted streaks would repeat than in the Stock50 Condition (Mean Difference = –0.16, *p* = 0.002) and in the Analyst50 Condition (Mean Difference = –0.13, *p* = 0.010). However, there was *not* a significant difference between the proportion of participants predicting streaks would repeat in the Analyst50 and Stock50 Conditions (Mean Difference = –0.02, *p* = 1.001).

There was a significant main effect of Streak Length on predictions, (*F*(4.47, 1332.46) = 17.86, *p* < 0.001).^38^ A higher proportion of participants predicted streaks would repeat as Streak Length increased. The interaction between Streak Length and Condition was also significant (*F*(8.94, 1332.46) = 3.84, *p* < 0.001). In the Stock50 and Analyst50 Conditions, the proportion of participants predicting a streak would repeat increased over Streak Lengths 2 through 5. In the Bingo50 Condition, the proportion of participants predicting a streak would repeat did not increase with Streak Length.

To understand participants’ individual prediction strategies, we again fitted logistic regressions to each participants’ predictions for the six Target sequences ending in a streak (see Appendix B).^39^ The distributions of percent‐change values are centered above 50% in the Analyst50 and Stock50 Conditions, and the distribution of values is centered near 15% in the Bingo50 Condition. The odds that a participant in the Bingo50 Condition would predict *repetition* for a streak of length *x* were only about 15% higher than the odds a participant in this Condition would predict repetition for a streak of length *x* – 1. Participants were more likely to predict reversals in the Bingo50 Condition than in the other two Conditions. In the Bingo50 Condition, 26% of participants exhibit a *negative* percent‐change in the odds they predict “repeat” as Streak Length increases. Only 14% of participants in the Analyst50 Condition, and 13% of participants in the Stock50 Condition, exhibit such a preference for reversal over repetition.

### 4.5 Discussion: Studies 2A and 2B

Participants facing all three generators showed a bias toward reversal for streaks of length 1 and 2 at the aggregate level in Study 2A. Participants presented with an intentional actor (Analyst50) or a market process (Stock50) eventually increased their expectations of repetition. These participants seemed to update their beliefs about the generators' base rate as Streak Length increased, but at a more conservative rate than participants facing an uncertain base rate in Study 1A. In contrast, participants faced with a random mechanical generator (Bingo50) showed a constant bias toward reversal. We believe that the numerical response scale in Study 2A may have encouraged participants to respond with probabilities near the prescribed .50 base rate.

Study 2B reveals an exaggerated version of the pattern in Study 2A. First, judgments of all generators initially exhibit dramatic gambler's fallacy reversal rate patterns for streaks of length 2–3. Starting at streaks of length 3, all three generators begin to show the updating pattern implied by Rabin's Small Urn Model, although the proportions for the mechanical bingo cage generator remain well below .50.

We checked for differences between response formats by dichotomizing the predictions made by participants in Study 2A.^40^ Welch's *t*‐test for unequal variances revealed that a significantly smaller proportion of participants in each Condition of Study 2B predicted streaks would repeat than in each corresponding Condition of Study 2A (Bingo_STUDY2B_ – Bingo_STUDY2A_ = –0.11, *s.e*. = 0.03, *p* < 0.001; Analyst_STUDY2B_ – Analyst_STUDY1A_ = –0.23, *s.e*. = 0.03, *p* < 0.001; Stock_STUDY2B_ – Stock_STUDY1A_ = –0.17, *s.e*. = 0.04, *p* < 0.001). Participants were not more likely to exhibit a preference for repetition when asked to respond with a dichotomous choice (Study 2B) than when asked to respond on a continuous numerical scale (Study 2A). Once again, the opposite seems to be the case. A greater proportion of participants in each Condition predicted streaks would repeat in Study 2A than in Study 2B.

As noted in the Introduction, we believe the most plausible explanation for the prediction patterns observed in Studies 2A and 2B is that participants respond to short streaks by imagining a sampling without replacement process or some analogue (Rabin, 2002, and others cited in the Introduction). Such a mental model could originate in a partial understanding of instructed principles; for example, that outcomes from random devices “average out” over time (Konold, 1995, and many others). Or, it could originate in a generalization from finite event sequences that exhibit a depletion pattern, such as the diminishing supply of some resource sampled repeatedly (Hahn & Warren, 2009). As streaks become longer, participants shift to updating their beliefs about the base rate.

As noted in our discussion of Studies 1A and 1B, we think the differences between average responses for the mechanical bingo cage versus the intentional actor and social market processes reflect different mental models of the random device generator versus the intentional actor and market generators. We speculate that participants interpreted the .50 performance rate of the companies and stock analysts as the *average* of two or more underlying performance states (e.g. striving versus slacking). This would make transitions between states (high versus low performance) more plausible for these generators than for the bingo cage, which could not transition between states (the ratio of blue to red balls never changes). Thus, our interpretation for the intentional and market generators reflects Rabin's proposal that priors over these base rates, even when specified as fixed probabilities, are likelier to be updated than base rates from a rigid mechanical device like a bingo cage.

## 5 Studies 3A and 3B

In Studies 3A and 3B, participants were told there were exactly three possible base rates for each generator (.25, .50, or .75), and that each of these rates was equally likely to generate the sequence revealed on each trial. We speculated that explicitly specifying a precise distribution of rates (three alternative performance states) for the company, stock analyst, and bingo cage generators would increase consistency across participants’ mental models of these generators, resulting in greater agreement in predictions across the three generators. Specifying the distribution of possible rates also allows us to use a Bayesian updating model to estimate a “rational” posterior probability that the terminal streak will repeat.

### 5.1 Participants

One hundred and fifty participants (M_AGE_ = 34.09, SD_AGE_ = 10.06, N_FEMALE_ = 74) took 18.76 minutes on average (SD = 9.45) to complete Study 3A. Three hundred participants (M_AGE_ = 36.83, SD_AGE_ = 11.86, N_FEMALE_ = 159) took 19.44 minutes on average (SD = 9.49) to complete Study 3B. Participants in both studies were paid $2.50 upon approval of their completed tasks.

### 5.2 Method

Participants were randomly assigned to one of three experimental Conditions, defined by the description and base rate distribution of the generator.^41^ In the Bingo25‐50‐75 Condition, the events in each sequence were described as draws (Red/Blue) made *with replacement* by a mechanical bingo machine from one of three cages, each having a different ratio of red to blue balls (25:75, 50:50, and 75:25). On each trial, the machine randomly selects one of the cages with equal probability, and then draws 8 outcomes from the selected cage *with replacement*. In the Analyst25‐50‐75 Condition, the events in each sequence were described as quarterly changes (Up/Down) in the value of a particular investment analyst's portfolio. Analysts were equally likely to have each of three skill levels, indicating the proportion of the time that their portfolios increase in value: Bad (.25), Average (.50), and Good (.75). In the Stock25‐50‐75 Condition, the events in each sequence were described as quarterly changes (Up/Down) in a particular company's stock price. Companies were equally likely to have each of three performance levels, indicating the proportion of the time that their stock price increased: Bad (.25), Average (.50), and Good (.75). Aside from these differences in the instructions, the experimental procedure was identical to the previous studies. Participants in Study 3A indicated their predictions using a continuous numerical scale, and participants in Study 3B indicated their predictions by making dichotomous choices.

### 5.3 Results: Study 3A

**Fig. 5:**
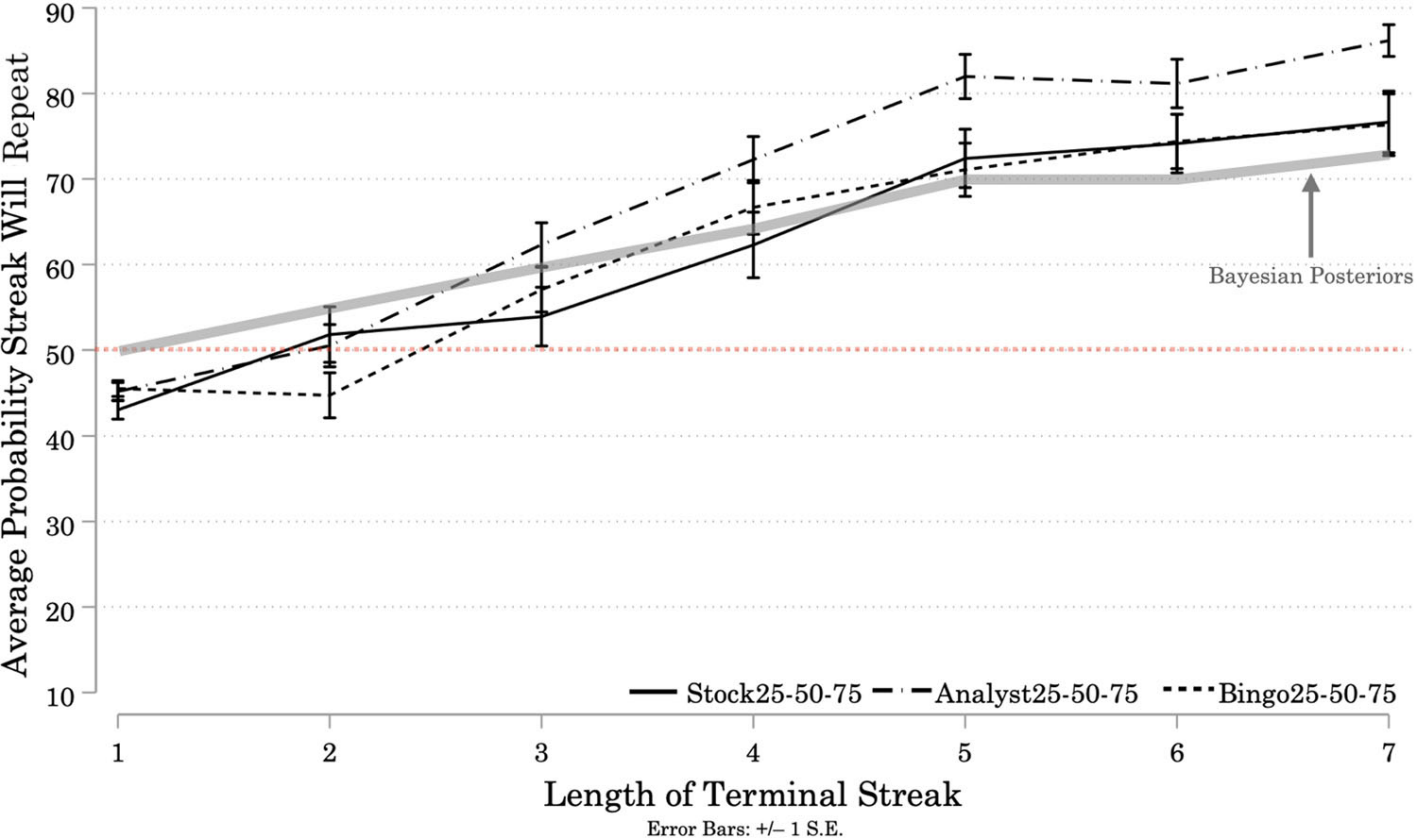
Study 3A: Average Probability Participants Assigned to Repetition of Terminal Streaks, by Streak Length and Condition *Note*: Results of Study 3A. N = 150 (Stock25‐50‐75 = 50; Analyst25‐50‐75 = 50; Bingo25‐50‐75 = 50). Solid and patterned lines represent the average probability participants in each Condition assigned to repetition of the streak at the end of each Target sequence. Error bars represent +/– 1 standard error. The solid gray bar represents the average Bayesian posterior probabilities of terminal streaks repeating, conditional on the pattern of outcomes in each Target sequence. Participants’ expectations of repetition increased with Streak Length. Probabilities assigned by participants in the Bingo25‐50‐75 and Stock25‐50‐75 Conditions converged to the maximum possible rate (.75) by Streak Length 6. Probabilities assigned by participants in the Analyst25‐50‐75 Condition exceeded the maximum possible rate starting at Streak Length 5, and increased beyond the maximum rate across Streak Lengths 6 and 7.

Figure 5 presents the average probabilities participants assigned to repetition of the terminal streak in each Target sequence. Participants in all three Conditions assigned greater than 50% probability to repetition of streaks of length 3 or greater. Predictions made by participants in all three Conditions follow a reasonable updating pattern as Streak Length increases, up to the point where the highest possible rate (.75) becomes the most likely to have produced the sequence. The Bayesian posterior probabilities of repetition are superimposed (solid gray line) over the predictions made by participants in each Condition. Participants in the Analyst25‐50‐75 Condition appear to overreact (compared to a perfect Bayesian) to streaks of length 4 or longer, assigning probabilities that are about 10 points higher than the Bayesian posteriors. This is the one feature in our results that could be interpreted as hinting that intentionality increases expectations of repetition above and beyond the results of a Bayesian belief‐updating process (though the results of Study 3B, discussed below, undermine this interpretation).

We conducted a one‐way mixed ANOVA to test the effects of one between‐subjects variable (Condition), and one within‐subjects variable (Streak Length) on ratings of the probability that the terminal streak would repeat.^42^ There was a significant effect of Condition on participants’ predictions (*F*(2, 147) = 3.59, *p* = 0.030). However, Bonferroni‐corrected pairwise comparisons reveal that differences between Conditions are only marginally significant. Participants in the Analyst25‐50‐75 Condition assigned slightly higher probabilities than those in the Bingo25‐50‐75 (Mean Difference = 7.36, *p* = 0.061) and Stock25‐50‐75 (Mean Difference = 7.20, *p* = 0.069) Conditions.

Recall that in Study 1A, Participants assigned significantly higher probabilities to repetition of streaks in the StockUnknown Condition than they did in the BingoUnknown Condition. In Study 3A, this difference was not significant (Mean Difference = –0.16, *p* = 1.001). When participants based their predictions on identical (uncertain) prior beliefs, we observe no reliable differences across the Bingo and Stock generators.

There was a significant main effect of Streak Length on predictions, (*F*(4.41, 647.80) = 82.32, *p* < 0.001).^43^ Longer Streak Lengths were assigned higher probability of repetition. The interaction between Streak Length and Condition was not significant (*F*(8.81, 647.80) = 1.28, *p* = 0.247). Participants’ predictions converge toward the rate with the highest posterior probability, conditional on the sequence of signals they observed (producing an apparent hot hand pattern).

**Fig. B5:**
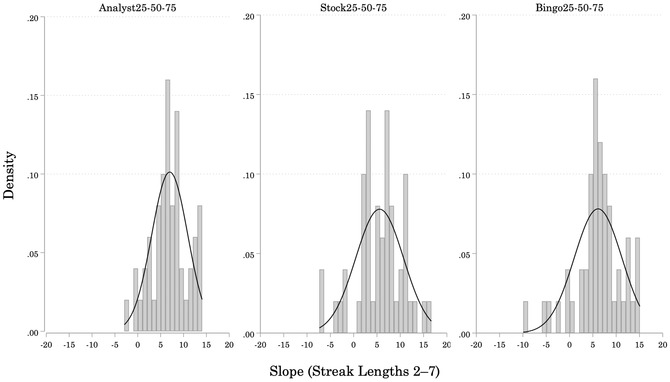
Distributions of Individual Participants’ Prediction Strategies in Study 3A *Note*: Study 3A. N = 150 (Stock25‐50‐75 = 50; Analyst25‐50‐75 = 50; Bingo25‐50‐75 = 50). Histograms of frequencies for slopes of linear regressions fitted to each participant's predictions over target stimuli (gray bars), overlaid with normal density curves (dark gray lines).

The distributions of slopes from regressions fitted to each participant's predictions over the Target sequences are centered just above 5 in all three Conditions (see Figure B5 in Appendix B). For each unit increase in Streak Length, participants increased the probability they assigned to repetition of that streak by a little over 5%. There is more heterogeneity in prediction strategies used by participants in the Stock25‐50‐75 Condition than by those in the Analyst25‐50‐75 and Bingo25‐50‐75 Conditions.^44^ There is also a higher proportion of negative “outlier” strategies – participants whose expectations of repetition *decrease* as Streak Length increases – in the Bingo25‐50‐75 Condition (12%) and in the Stock25‐50‐75 Condition (14%) than in the Analyst25‐50‐75 Condition (4%).

### 5.4 Results: Study 3B

**Fig. 6:**
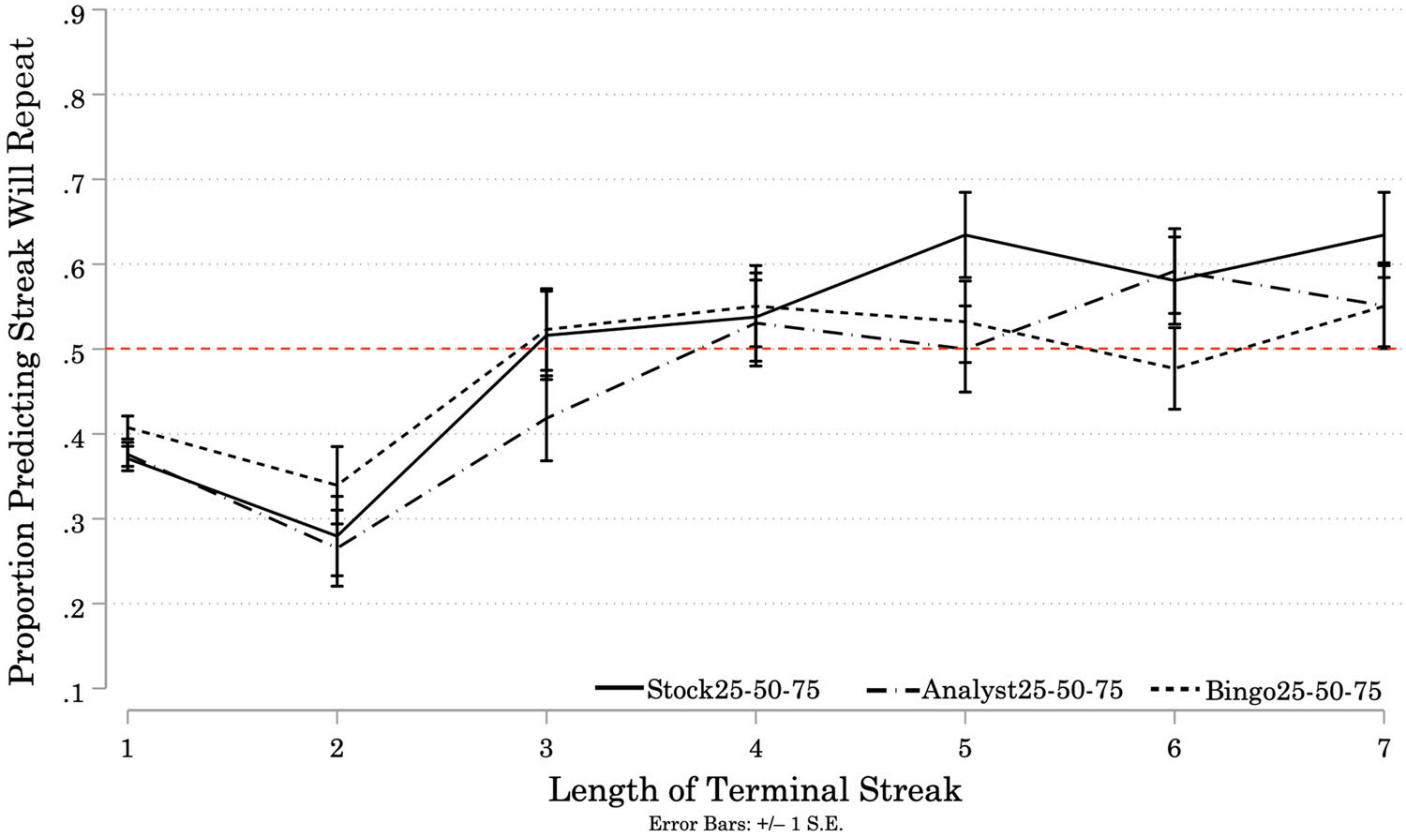
Study 3B: Proportion of Participants Predicting Repetition of Terminal Streak, by Streak Length and Condition *Note*: Results of Study 3B. N = 300 (Stock25‐50‐75 = 93; Analyst25‐50‐75 = 98; Bingo25‐50‐75 = 109). Solid and patterned lines represent the proportion of participants in each Condition who predicted repetition of the streak at the end of each Target sequence. Error bars represent +/– 1 standard error. In all three Conditions, the proportion of participants predicting repetition of the terminal streak increased between Streak Lengths 2 and 3. Across Streak Lengths 4 through 7, the proportion of participants predicting repetition does not consistently increase in the Analyst25‐50‐75 or Bingo25‐50‐75 Conditions, but there does seem to be a moderate increase in the Stock25‐50‐75 Condition. Unlike Study 3A, participants faced with an intentional actor were not more likely to predict repetition than those faced with a market or random mechanical generator.

Figure 6 presents the results of Study 3B. In all three Conditions, the proportion of participants predicting repetition of the terminal streak increased between Streak Lengths 2 and 3 (Figure 6). Across Streak Lengths 4 through 7, the proportion of participants predicting repetition does not consistently increase in the Analyst25‐50‐75 and Bingo25‐50‐75 Conditions, but there is a moderate increase in the Stock25‐50‐75 Condition.

We conducted a one‐way mixed ANOVA to test the effects of Condition and Streak Length on participants’ predictions that the streak at the end of each Target sequence would *repeat*.^45^ The effect of Condition on participant predictions was not significant (*F*(2, 297) = 0.62, *p* = 0.538). There was a significant main effect of Streak Length on predictions, (*F*(4.49, 1333.14) = 22.06, *p* < 0.001).^46^ A higher proportion of participants predicted streaks would repeat as Streak Length increased. However, Bonferroni‐corrected pairwise comparisons revealed that the only significant differences were between Streak Length 2 and each of the other Streak Lengths. There was no significant interaction between Streak Length and Condition (*F*(8.98, 1333.14) = 1.58, *p* = 0.115).

**Fig. B6:**
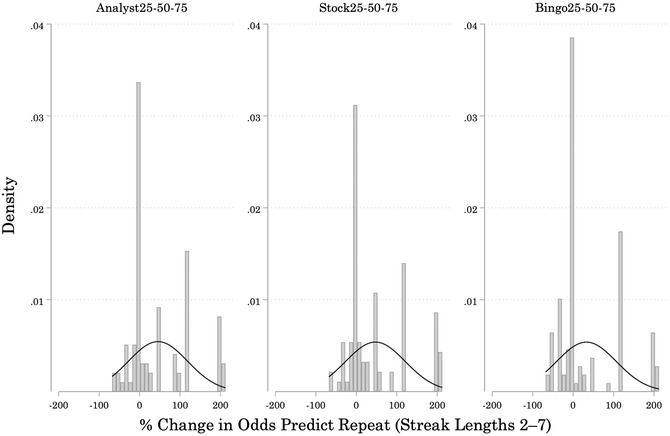
Distributions of Individual Participant's Prediction Strategies in Study 3B *Note*: Study 3B. N = 300 (Stock25‐50‐75 = 93; Analyst25‐50‐75 = 98; Bingo25‐50‐75 = 109). Histograms of frequencies for different values of percent‐changes (gray bars), overlaid with normal density curves (dark gray lines).

We again fitted logistic regressions to each participant's predictions for the six Target sequences, and transformed the resulting coefficients from the log odds to the percent‐change in the odds of predicting repetition for each unit increase in Streak Length (see Figure B6 in Appendix B).^47^ The distributions of individual percent‐change values are centered above 50% in the Analyst25‐50‐75 and Stock25‐50‐75 Conditions, and the distribution of values is centered above 25% in the Bingo25‐50‐75 Condition. The odds that a participant in the Bingo25‐50‐75 Condition predicts repetition for a streak of length *x* were about 25% higher than the odds a participant in this Condition predicts repetition for a streak of length *x* – 1. A minority of participants exhibit negative percent‐changes in the odds they predict “repeat” as Streak Length increases: 18% in the Analyst25‐50‐75 Condition, 16% in the Stock25‐50‐75 Condition, and 28% in the Bingo25‐50‐75 Condition.

### 5.5 Discussion: Studies 3A and 3B

Participants’ predictions in Studies 3A and 3B are consistent with a Bayesian updating pattern for all three generators. When provided with an explicit distribution of possible base rates, participants seemed to use this information appropriately. The significant differences we observed between predictions made by participants facing the Bingo and Stock generators in previous studies disappeared in Studies 3A and 3B.

We saw a slight overreaction to longer streaks when participants in the Analyst25‐50‐75 Condition of Study 3A were asked to respond using a continuous numerical scale. This could be interpreted as evidence that participants exhibit a stronger positive bias for repetition when evaluating outcomes produced by an intentional actor. However, this overreaction was not observed when participants were asked to respond with a dichotomous choice in the Analyst25‐50‐75 Condition of Study 3B. (Of course, this comparison requires us to make an assumption that the proportions of response rates across observers responding with a dichotomous choice can be interpreted as degrees of belief attributed to a single individual.) We conclude that, taken together, the results of Studies 3A and 3B do not support the hypothesis that intentionality of the generator increases expectations that streaks will repeat. Instead, the results support our favored hypothesis that the hot hand pattern arises from uncertainty over the base rate of the generator, combined with a reasonable updating process.

As before, we dichotomized the predictions of participants in Study 3A in order to compare them to predictions made by participants in Study 3B.^48^ Welch's *t*‐test for unequal variances indicated that a smaller proportion of participants in each Condition of Study 3B predicted streaks would repeat than in each corresponding Condition of Study 3A (Bingo_STUDY3B_ – Bingo_STUDY3A_ = –0.26, *s.e*. = 0.03, *p* < 0.001; Analyst_STUDY3B_ – Analyst_STUDY3A_ = –0.37, *s.e*. = 0.03, *p* < 0.001; Stock_STUDY3B_ – Stock_STUDY3A_ = –0.19, *s.e*. = 0.03, *p* < 0.001). These differences were significant at almost every Streak Length. Again, participants were not more likely to exhibit a preference for streaks to repeat when asked to respond with a dichotomous choice (Study 3B) than when asked to respond using a continuous numerical scale (Study 3A).

## 6 General Discussion and Future Directions

The present studies are the first to provide comparisons of precise manipulations of information about base rates of outcomes produced by three types of generators: a random mechanical device, an intentional actor, and a financial market. These studies are distinctive in providing comparisons of these manipulations across carefully controlled, comparable tasks, instructions, and participant samples. The research program is also the first to provide a controlled comparison of judgments expressed on continuous (numerical) versus dichotomous outcome response scales. We conclude that the dominant cognitive process underlying most predictions for sequences of binary outcomes is pragmatic belief‐updating about an uncertain base rate parameter.

We also identify a pocket of anomalous predictions for sequences generated by a random mechanical device with an explicit, fixed base rate of .50. We interpret those responses as representing gambler's fallacy reasoning, that is best conceptualized as a mental model of the generator based on an outcome‐depletion process like sampling without replacement from an abstract urn. As noted in our prior discussions of theoretical interpretations we endorse the “sampling without replacement” urn model that was proposed by Rabin (2002) and several precursors as a general description of this mental model.

A third model process (in addition to belief updating and outcome‐depletion) that is sometimes referenced when interpreting sequences of outcomes is a momentum process. Like the outcome‐depletion model, the momentum concept refers to a change in outcome rates, but increasing, rather than decreasing as implied by the outcome‐depletion principle. Although there were some hints of over‐reaction to outcomes (compared to the Bayesian updating process model), these effects were small, not statistically reliable, and were not moderated by the valence of the signal type (whether the streak was comprised of successes or failures). (Most momentum interpretations focus on streaks of *successful* outcomes for goal‐directed, intentional actors.)

We should note that the present studies do not verify details about plausible mental models that participants might believe describe the mechanisms that generate the events to be judged (random mechanical process, goal‐directed intentional actor, social‐economic market). Rabin (2002) proposed that stochastic urns with various sampling rules might provide a useful descriptive abstraction. Elsewhere, Oskarsson, Van Boven, McClelland, & Hastie (2009) proposed Markov Process graphical models as a general representational medium. The empirical analysis of beliefs about causal generating models is an obvious next step in research on forecasting events in sequences.

### 6.1 Why do participants exhibit gambler's fallacy prediction patterns for random generators with a fixed base rate?

Like other theorists we believe the outcome‐depletion belief arises from a combination of several experiences. First as noted in the introduction, some people are conditioned to produce this pattern through scholastic training in mathematics that essentially teaches students to use Law of Small Numbers reasoning, informally referred to as “The Law of Averages.” In a review of mathematics textbooks used in the United States between 1957 and 2004, Jones (2004) notes that teachers are most often directed to introduce the concept of probability (and randomness) using one of the following pseudo‐random devices: marbles in a jar, papers in a hat, cubic dice, coins, or spinners. The devices almost always have stationary, equiprobable base rates (e.g., .50 for each face of the coin). “Students therefore learn … that the purpose of drawing a random sample is to *ensure* representativeness in order to gain knowledge about the population from the sample” (Harradine, Batanero, & Rossman, p. 240, emphasis added). Thus, students learn to expect small samples of outcomes from equiprobable random mechanical devices will “represent” their population parameters (Stohl, 2005).

Our description of the bingo cage as a random mechanical device with a stationary, equiprobable base rate (Studies 2A and 2B) could have evoked these Law of Small Numbers beliefs, leading to the expectation that a sequence containing a streak of identical outcomes would “correct itself” and “balance out.” The developmental trend in predictions for sequences of binary outcomes like coin tosses is consistent with this interpretation. Preschool children have reasonable intuitions about probability, and exhibit a bias toward repetition of streaks (Bogartz, 1965; Chiesi & Primi, 2009; Craig & Meyers, 1963; Derks & Paclisanu, 1967; Estes, 1962; Fischbein, 1975; Fischbein & Schnarch, 1997). The gambler's fallacy pattern of predicting reversals increases with age (Chiesi & Primi, 2009; Derks & Paclisanu, 1967).

Participants’ verbal reports on their own prediction strategies in the present studies also support this Law of Small Numbers interpretation (acknowledging that *post hoc* verbal reports are only suggestive; cf. Nisbett & Wilson, 1977). At the end of the experimental procedure, participants were asked: “What was your strategy for predicting what would happen next? What information did you use to make your prediction?” Participants’ responses were classified into one of eight categories: (1) balancing outcomes; (2) guessing; (3) estimating a proportion or counting outcomes, including references to updating estimates; (4) momentum or increasing probability of one outcome over the other; (5) “following instructions” (often reported to justify sticking with the specified .50 rate in Studies 2A and 2B); (6) deciding which “type” of generator produced the sequence, particularly with reference to the distribution of rates provided in Studies 3A and 3B (e.g. high‐ versus low‐performing analysts); (7) performing a weighting calculation that takes into account the different types of generators, particularly in Studies 3A and 3B; and (8) “other” unclassifiable responses. (See Appendix C for a summary of the methods and results of this “think‐aloud” exercise.)

References to estimating or updating proportions comprise more than 50% of the responses for all experimental Conditions across our three Studies (categories labeled “Proportion” and “Momentum” in Appendix Table C1). The prevalence of these verbal reports fits with our hypothesis that base rate updating is the primary inference process underlying the pervasive positive recency prediction patterns in our experiments. Second, “balancing” reports are scattered across experimental conditions and occur at highest rates among participants faced with sequences produced by a random mechanical device in the Bingo Conditions of each study (18% and 30% for the fixed .50 base rate in Studies 2A and 2B, respectively). This is consistent with our conjecture that Law of Small Numbers reasoning is most likely to occur for random mechanical devices with an explicit, fixed base rate. Third, unsurprisingly, self‐reports of reasoning about “types” of generators (coding categories labeled “Type” and “Weighting”) are common when “types” (e.g., Bad, Average, and Good analysts) are mentioned explicitly in the experimental instructions (Studies 3A and 3B).

Another explanation for anomalous judgments of sequences generated by random mechanical devices is that observers transfer valid beliefs from analogous situations they have encountered outside the laboratory, in which outcome‐depletion actually occurs, to the experimental task, or by accurately extracting the statistical properties of short sequences of events (Hahn & Warren, 2009; Kareev, 2001; Miller & Sanjurjo, 2018a; and Reimers, Donkin, & Le Pelley, 2018, spell out detailed versions of this interpretation). This transfer process could be a simple generalization from the statistical properties of one situation to the new, to‐be‐judged situation (Farmer, Warren, & Hahn, 2017; Turk‐Browne, Scholl, Chun, & Johnson, 2009). Simple statistical induction would be consistent with our observation of reversal predictions for streaks of length 2–3 across all generators on the dichotomous outcome choice measure. Or, the statistical regularities in one situation could be used to construct a mental model of a causal mechanism, and then that abstracted mechanism would be applied to deduce predictions in a new context.

The weakness of these transfer‐of‐statistical‐patterns interpretations is that no one knows *ex ante* on which extra‐laboratory learning experiences observers will rely. Without that information, it is not possible to provide a strong test of this highly plausible, but imprecise, family of interpretations. One might ask, for example, why the anomalous gambler's fallacy predictions occur at the highest rates for random mechanical generators, when small samples and negative recency patterns occur for many other generators outside the laboratory.

### 6.2 Response Format Effects

For the most part, predictions made by participants using a continuous probability scale are similar to those made by participants asked to make a dichotomous choice. Our hypothesis was that the numerical response scales would evoke more analytic judgment strategies than the “choose one outcome” binary format instructions. Furthermore, the numerical response scale would remind participants of the prior base rate when one was specified in the instructions. This hypothesis is consistent with the results in the fixed .50 base rate experiments (2A and 2B).

The high rates of reversal predictions on the dichotomous response scale format given a random generator with a fixed base rate (consistently greater than 70% of participants predicting reversal for all streaks longer than 1) support the conclusion that gambler's fallacy response patterns are more common when attention is focused on discrete outcomes, rather than a magnitude or propensity as on the continuous probability response scale.

If we interpret the *proportions* of participants who predict *repetition* as degrees of belief, we find a couple of anomalies in the predictions made by participants responding with a dichotomous choice. First, the proportion of participants predicting repetition for streaks of length 2 is surprisingly low (between .30 and .35) on dichotomous response scales across all three Studies. Second, when given no information about the base rate, participants asked to make a dichotomous choice (Study 1B) seem to update their beliefs at about the same rate as participants responding with numerical probability estimates (Study 1A). However, participants given a stationary base rate, or a specified distribution of possible rates, update more conservatively when asked to make a dichotomous choice (Studies 2B and 3B) than when asked to provide a numerical probability estimate (Studies 2A and 3A). This difference is especially pronounced for longer streaks, with relatively flat proportions of participants predicting repetition across Streak Lengths 5–7 in Studies 2B and 3B (dichotomous choice).

### 6.3 Limitations

The present studies are subject to several limitations. First, we only presented participants with relatively short sequences of 8 outcomes, so we cannot draw conclusions about people's judgment behavior when exposed to longer sequences of outcomes. Second, despite our efforts to balance statistical properties across the full set of sequence stimuli, we still only used a small subset of possible sequences, and sequences with streaks of length 4 and greater were overrepresented (compared to a true random binomial process). Post hoc analyses that include our filler sequences (ending in reversals) show that *non‐terminal* streaks and simple *global proportions* of outcomes across all eight events were associated with belief‐updating prediction patterns, although the rate of updating was greater when the streak of similar events occurred at the end of a sequence (see Chapter 3 of the Supplementary Material).

We also sacrificed control over the experimental task by recruiting all of our participants online. Remote crowdsourced workers participating online are subject to a variety of distractions. And, it is difficult to define the population represented by participants sampled from the Amazon Mechanical Turk site, especially with regards to their past exposure to various experimental paradigms that might bias their behavior.

There is also an unavoidable methods problem that arises when any researcher presents participants with descriptions of event sequences, rather than having them experience the sequences in a palpable realistic situation (e.g., placing bets in a casino, tossing physical coins in a classroom). This means that some participants will be “updating” their beliefs by entertaining hypotheses about experimenter artifice and deception. The possibility of undetected participant suspicion is present in every situation labeled “a study,” or “an experiment.” We believe that the rate of participants rejecting our instructions is minimal in our studies because participants passed tests to verify comprehension of the instructions, and few reported suspicion when open‐ended inquiries were made about their reactions to the experimental experience. Individual and average responses were also consistent with patterns exhibited in many related studies, and with the conceptual interpretations we have proposed.

Finally, the artificial design of our experimental procedure, though consistent with prior work, strips away potentially impactful features of the judgment situation – for example, the emotionally‐charged experience of watching a live basketball game, the thrill of a big payout from a successful bet, or the social and emotional consequences of a major loss on a stock market investment. It is possible cues that evoke emotional or motivational responses in the observers are necessary for some judgment phenomena to occur (e.g., momentum effects).

### 6.4 Concluding Remarks

We believe the present research provides one of the most comprehensive overviews in the scientific literature on human prediction patterns for sequences of binary events. Our goal was to learn something general about the conditions that produce the two most common prediction patterns, the hot hand and the gambler's fallacy. The hot hand judgment pattern is most likely to occur after an observer sees a streak of similar outcomes from a generator with an ambiguous or uncertain base rate. We conclude that this pattern is best interpreted as the result of a reasonable, bottom‐up, evidence‐based process for updating beliefs about the generator's base rate. We observe this pattern for all three types of generators when the generator's base rate is ambiguous or uncertain, both when participants express their predictions as continuous probability estimates and as dichotomous choices.

However, when people hold strong prior beliefs about a stationary base rate, prediction patterns for a random mechanical device are different than those for an intentional actor or a market. Participants faced with a random mechanical device that has a stationary base rate exhibit a persistent bias toward reversal of streaks, especially strong when responding with a dichotomous choice. This anomalous habit may result from mis‐interpretations of principles of probability that participants learned in mathematics classes. Or, it may be that memories of non‐laboratory experiences are being transferred to the controlled, focused experiences provided in our experiments. Our experiments were not designed to discriminate between these two accounts.

In the present research, belief updating provides a sufficient and plausible explanation of positive recency prediction patterns. Notably, we found no compelling evidence for causal momentum or intentionality effects for any of our generators, beyond those implied by reasonable updating of beliefs about the generators’ base rates.^49^

We found that participants expected streaks produced by a financial market to repeat. Here again, we think belief updating is the dominant cognitive process. We suspect the difference between our results and those of some related experiments where participants exhibited a contrarian bias toward market streak reversal is due to differences in experimental instructions or to differences in participant samples. There is much heterogeneity in expertise and personal investment theories associated with the varied results reported in other studies of stock market forecasting.

The present studies advance our understanding of the conditions under which two general judgment patterns, hot hand and gambler's fallacy, are likely to dominate individual judgments. The gambler's fallacy pattern appears clearly in many observers’ predictions when judging a sequence generated by a random mechanical device that has an explicit, fixed base rate. But, we do not believe it is necessary to posit spooky causal beliefs, irrationally linking the outcomes produced by a random generator. We find it most plausible that gambler's fallacy patterns of predictions derive from experiences with a subset of naturally‐occurring sequences that actually exhibit negative recency, and from classroom instruction that teaches students to believe in the Law of Small Numbers.

### Open Research Badges

This article has earned Open Data and Open Materials badges. Data and materials are available at https://osf.io/2d93t/.

## Supporting information



Supporting Material

Supporting Material

## Figures and Tables

**Fig. B1:**
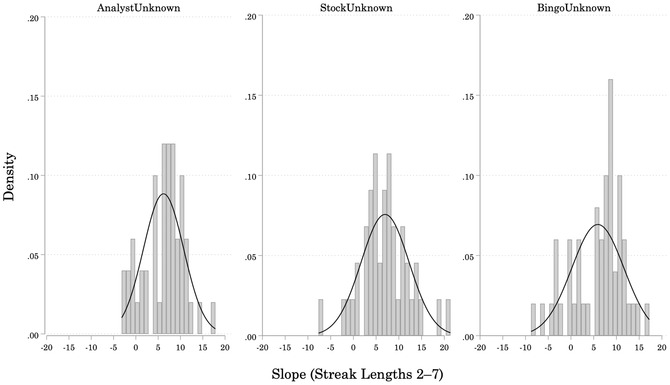
Distributions of Individual Participants’ Prediction Strategies in Study 1A *Note*: Study 1A. N = 144 (StockUnknown = 44; AnalystUnknown = 50; BingoUnknown = 50). Histograms of frequencies for fitted slopes of participants’ predictions over target stimuli ending in streaks of length 2–7 (gray bars), overlaid with normal density curves (dark gray lines).

**Fig. B2:**
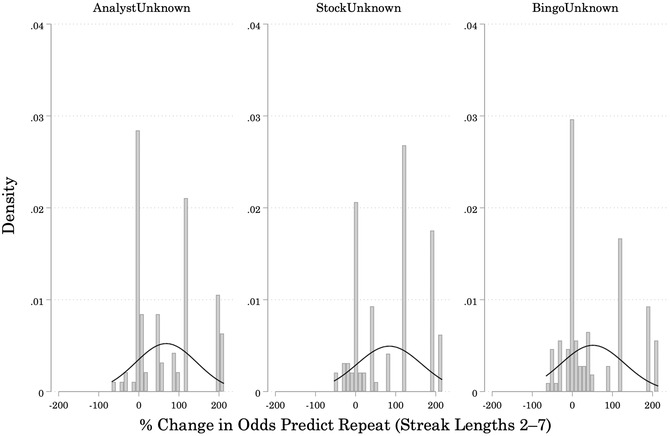
Distributions of Individual Participants’ Prediction Strategies in Study 1B *Note*: Study 1B. N = 300 (StockUnknown = 97; AnalystUnknown = 95; BingoUnknown = 108). Histograms of frequencies for different values of percent‐changes (gray bars), overlaid with normal density curves (dark gray lines).

**Fig. B3:**
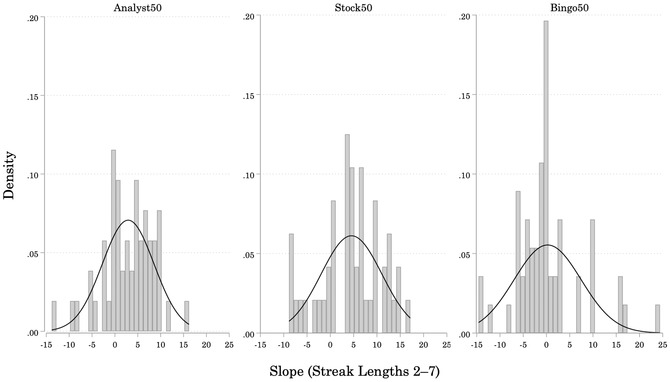
Distribution of Individual Participants’ Prediction Strategies in Study 2A *Note*: Study 2A. N = 156 (Stock50 = 48; Analyst50 = 52; Bingo50 = 56). Histograms of frequencies for fitted slopes of participants’ predictions over target stimuli ending in streaks of length 2–7 (gray bars), overlaid with normal density curves (dark gray lines).

**Fig. B4:**
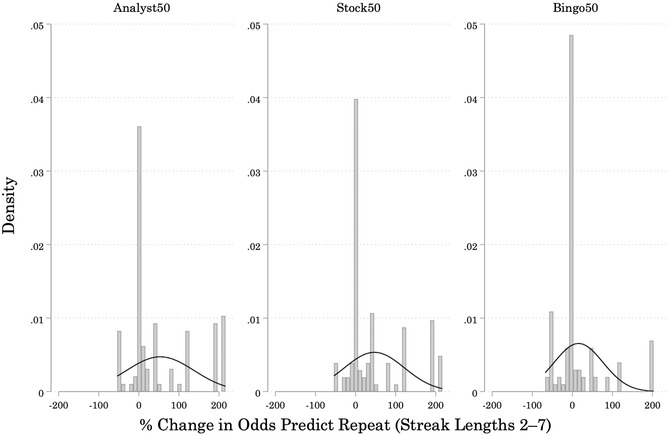
Distributions of Individual Participants’ Prediction Strategies in Study 2B *Note*: Study 2B. N = 301 (Stock50 = 103; Analyst50 = 97; Bingo50 = 101). Histograms of frequencies for different values of percent‐changes (gray bars), overlaid with normal density curves (dark gray lines).

## Notes

1 The alternation rate is defined as *p*(A) = (*r –* 1) / (*n* – 1), where *r* is the number of “runs” (a streak of identical outcomes), and *n* is the number of signals (outcomes) in the sequence. For example, the sequence *aabaaab* has *r* = 4 runs (*aa, b, aaa*, and *b*), and *n* = 7 signals. The alternation rate for this sequence is *p*(A) = (4 – 1) / (7 – 1) = 3/6 = .50.

2 Burns and Corpus (2004) and Tyszka, Zielonka, Dacey, and Sawicki (2008) both asked experimental participants to judge sequences produced by two different human actors: one rated as *more* random (your little sister shooting baskets in Burns & Corpus; a fortune‐teller in Tyszka et al.), and another rated as *less* random (a competitive car salesman in Burns & Corpus; a basketball player shooting baskets in Tyszka et al.). Participants exhibited hot hand beliefs in their predictions for the *less* random human actors. But, the judgment pattern for the *more* random human actors was either neutral (Burns & Corpus) or inconsistent (Tyszka et al.). Importantly, the little sister shooting baskets in Burns and Corpus's study was explicitly described as *intending* to improve her success rate, and the Polish participants in Tyszka and colleagues’ experiment were familiar with effortful performances by fortune‐tellers who use various physical props to underscore their particular skill in accurately forecasting the future. So, the differences in participants’ predictions for these generators cannot be explained by participants failing to perceive the little sister or fortune‐teller as intentional, goal‐directed actors.

3 Interested readers may find a discussion of the belief‐updating process that leads the observer to overinfer the likely extremity of the base rate in Section IV of Rabin (2002, p. 788).

4 Gilovich and colleagues' (1985) proposition that the sports fan starts out expecting the athlete to behave like a random device, and then shifts to expecting streaks, also implies a shift from expecting reversal to expecting repetition (updating a mental model of the shooter as “consistent” versus “streaky”). The important difference is that Rabin's observer is updating a base rate in a Bayesian fashion.

5 Though Rabin presents a coherent theoretical account of how gambler's fallacy and hot hand patterns arise, he doesn't provide specific guidance for how we might predict whether or not an observer will find it *a priori* plausible that a generator's rate changes over time. For that guidance, we again are referred back to the judgment data that have been previously accumulated for different types of generators.

6 For an explanation of how these wait times are estimated, see Hahn and Warren (2009).

7 We'll return to this point, and provide additional context, at the end of the present article when we reflect on the results of our experiments, particularly Studies 2A and 2B.

8 In experiments that elicit predictions for market outcomes, the description of the market generator sometimes provides explicit information about the base rate (e.g. by evoking the concept of a fair coin, or describing a random walk process), and sometimes provides no information about the base rate. These discrepancies are one possible source of variation in participants' prediction habits for market generators.

9 We use multiple versions of each Target sequence, varying the type of outcome in the streak as well as the pattern of outcomes preceding the streak. Please see Chapter 1 of the Supplementary Material for a detailed description of the stimuli and the method of randomization used for presenting these stimuli to participants.

10 The strongest empirical support for this hypothesis comes from some surprising results reported by Murphy and Ross (2010) in which a vast majority of respondents shifted from an irrational selective attention strategy when making dichotomous responses to an almost rational Bayesian strategy when making numerical, category‐based probability inferences (also see Hammond, Hamm, & Grassia, 1987; Önkal & Muradoglu 1996; Windschitl & Wells, 1996).

11 The experimental instructions provided to participants in each study can be found in Chapter 2 of the Supplementary Material.

12 The present studies were approved by the Institutional Review Board at the authors' home university, and conducted between 2018 and 2019.

13 In Studies 3A and 3B, the machine draws from one of three different covered cages with equal probability. This procedural difference is described in more detail in Section 5, and the full set of instructions is available in Chapter 2 of the Supplementary Material.

14 If participants answered any questions incorrectly, they were asked to check their answers and correct any mistakes. Participants were allowed to attempt the questions as many times as they wished. This process ensured that no participants started the experimental session without first answering all of the comprehension check questions correctly. A detailed description of the instructions and comprehension check questions can be found in Chapter 2 of the Supplementary Material.

15 Filler sequences were randomly selected from a pool of 24 sequences ending in reversal. Details can be found in Chapter 1 of the Supplementary Material. On the first trial, participants always saw one of the Filler sequences. The 11 remaining Filler sequences, and 6 Target experimental sequences, were then presented in random order on trials 2 through 18.

16 Barron and Leider (2010) found that gambler's fallacy patterns of prediction emerged when sequences were presented sequentially, but not when they were presented all‐at‐once, so it is important to note that we are using *sequential revelation* in all of our experiments.

17 The specific question prompts, as well as screenshots of the experimental interface, can be found in Chapter 2 of the Supplementary Material.

18 Please see Chapter 6 of the Supplementary Material for a detailed description of the probability, financial literacy, and demographic questions. Chapter 6 also provides summary statistics and correlation matrices for these measures. There were no significant relationships between any of these measures and the results of the present experiments, so they are not discussed further here.

19 A summary of these qualitative responses can be found in Appendix C: Verbal Reports.

20 For this and all following studies, our target recruitment numbers were set in advance based on rules of thumb (see, e.g., Cohen, 1988; Green, 1991; Harris, 1985), and subject to budgetary restrictions. Our target was 50 participants per Condition for the Studies eliciting predictions on a continuous probability scale (1A, 2A, and 3A). More data are required to estimate average responses from binary choices than from continuous ratings. So, we doubled our per‐Condition target to 100 in the Studies eliciting predictions as a binary choice (1B, 2B, and 3B). We continued recruitment until we came as close as possible to our recruitment targets. We did not analyze any partial data.

21 Participants’ predictions over the Filler sequences (all ending in a reversal) were *not* included in this or any subsequent analysis. Interested readers can find summary statistics for participants’ predictions over the filler sequences in Chapter 3 of the Supplementary Material.

22 Mauchly's test indicated that the assumption of sphericity had been violated (χ^2^(14) = 49.03, *p* < 0.001); therefore, degrees of freedom were corrected using Greenhouse‐Geisser estimates of sphericity (ε = 0.89).

23 See Chapters 5 and 6 of the Supplementary Material for the analyses of individual differences in prediction patterns in all 6 experiments.

24 See Table A2 in Appendix A for numerical results. We present the results of the ANOVA here for ease of exposition, but it is not the appropriate analysis for these data. Because participants’ responses are dichotomized (1, 0), the distributions of these responses are not normal. This violates the assumptions of the least‐squares model used in ANOVA. Interested readers can find the results of a repeated measures binary logistic regression analysis of these data, as well as the binary choice data from Studies 2B and 3B, in Chapter 4 of the Supplementary Material. There are no differences between the substantive conclusions drawn from the ANOVA and the repeated measures binary logistic regression analyses for any of the Studies presented in this article.

25 Mauchly's test indicated that the assumption of sphericity had been violated (χ^2^(14) = 76.63, *p* < 0.001); therefore, degrees of freedom were corrected using Greenhouse‐Geisser estimates of sphericity (ε = 0.90).

26 Separation was observed while running participant‐level logistic regressions on 127/300 participants’ predictions over Target sequences. Firth's procedure was applied to all of the participant‐level logistic regressions to resolve the separation issue, producing less biased coefficients (for an explanation of this procedure, see Heinze & Schemper, 2002).

27 We first exponentiate the coefficient to obtain the odds ratio, then we subtract 1 from the odds ratio and multiply by 100 to get the percent‐change in the odds: [(*e^β^* – 1) × 100]. Example: The log odds that Participant A predicts “repeat” increase by 0.78 for each unit increase in Streak Length. The odds that this participant predicts a streak will repeat are exp(0.78) = 2.18 times higher for each unit increase in Streak Length. The percent‐change in the odds this participant predicts “repeat” is [(2.18–1) × 100] = 118% for each unit increase in Streak Length.

28 See Appendix B and Chapter 5 of the Supplementary Material for the corresponding figures.

29 All predictions equal to 50% were dropped (12 predictions across 8 participants). Our method for recoding the results from Study 1A is not arbitrary, the 50% threshold seems like a neutral and meaningful choice. But, it may not accurately reflect participants’ decision rule, and so the following discussion is speculative.

30 We selected Welch's *t*‐test because it does not assume equal variances, and is robust to large differences in sample sizes across groups. Note that there were 300 participants in Study 1B, but only 144 participants in Study 1A.

31 See Chapter 2 of the Supplementary Material for transcripts of participant instructions.

32 See Table A3 in Appendix A for numerical results.

33 A one‐sample, one‐tailed t‐test also confirmed that the mean of participants’ predictions across all Streak Lengths in the Bingo50 Condition was significantly less than 50% (*t* = –4.06, *p* < 0.001).

34 Mauchly's test indicated that the assumption of sphericity had been violated (χ^2^(14) = 89.94, *p* < 0.001); therefore, degrees of freedom were corrected using Greenhouse‐Geisser estimates of sphericity (ε = 0.82).

35 Compare Figures B1 and B3 in Appendix B.

36 See Table A4 in Appendix A for numerical results.

37 The results of a repeated measures binary logistic regression analysis of these data can be found in Chapter 4 of the Supplementary Material. There are no substantive differences between the results of the binary logistic analysis and those presented here.

38 Mauchly's test indicated that the assumption of sphericity had been violated (χ^2^(14) = 79.88, *p* < 0.001); therefore, degrees of freedom were corrected using Greenhouse‐Geisser estimates of sphericity (ε = 0.89).

39 Separation was observed while running participant‐level logistic regressions on 83/301 participants’ predictions over Target sequences. Firth's procedure was applied to all of the participant‐level logistic regressions to resolve the separation issue.

40 Predictions were recoded as before, with predictions equal to 50% dropped from the analysis (47 predictions across 20 participants).

41 Full transcripts of the experimental instructions can be found in Chapter 2 of the Supplementary Material.

42 See Table A5 in Appendix A for numerical results.

43 Mauchly's test indicated that the assumption of sphericity had been violated (χ^2^(14) = 52.36, *p* < 0.001); therefore, degrees of freedom were corrected using Greenhouse‐Geisser estimates of sphericity (ε = 0.88).

44 We speculate this pattern results from the prevalence of contradictory beliefs about the stock market.

45 The results of a repeated measures binary logistic regression analysis of these data can be found in Chapter 4 of the Supplementary Material. There are no substantive differences between the results of the binary logistic analysis and those presented here.

46 Mauchly's test indicated that the assumption of sphericity had been violated (χ^2^(14) = 80.15, *p* < 0.001); therefore, degrees of freedom were corrected using Greenhouse‐Geisser estimates of sphericity (ε = 0.90).

47 Separation was observed while running participant‐level logistic regressions on 92/300 participants’ predictions over target sequences. Firth's procedure was applied to all of the participant‐level logistic regressions to resolve the separation issue.

48 Predictions were recoded as before, and those equal to 50% were dropped (17 predictions across 14 participants).

49 This is why the term “hot hand” does not appear in the title of this paper.

## Appendix A Average Results for Each Experimental Condition

Tables A1, A2, A3, A4, A5, A6 present the numerical results for each of the six experiments discussed in the present article.

**Table A1:** Study 1A: Average Probability Participants Assigned to Repetition of Terminal Streaks, by Streak Length and Condition

		Average Probability Terminal Streak Will Repeat					
Condition	N	Streak = 2	Streak = 3	Streak = 4	Streak = 5	Streak = 6	Streak = 7
AnalystUnknown	50	49.06	57.08	63.80	71.74	73.34	81.16
		(23.47)	(23.73)	(27.34)	(25.80)	(25.78)	(20.87)
StockUnknown	44	47.95	61.16	69.07	78.73	79.11	84.05
		(20.90)	(21.60)	(22.28)	(18.48)	(23.49)	(20.51)
BingoUnknown	50	45.90	48.36	58.26	67.40	65.60	75.14
		(20.34)	(22.80)	(26.35)	(28.05)	(30.73)	(27.89)
All	144	47.64	55.30	63.49	72.37	72.42	79.95
		(21.53)	(23.25)	(25.74)	(24.93)	(17.34)	(23.58)

*Note*: Standard deviations in parentheses.

**Table A2:** Study 1B: Proportion of Participants Predicting Repetition of Terminal Streaks, by Streak Length and Condition

		Proportion Who Predicted Streak Will Repeat					
Condition	N	Streak = 2	Streak = 3	Streak = 4	Streak = 5	Streak = 6	Streak = 7
AnalystUnknown	95	0.33	0.53	0.60	0.72	0.75	0.80
		(0.47)	(0.50)	(0.49)	(0.45)	(0.43)	(0.40)
StockUnknown	97	0.32	0.55	0.66	0.78	0.84	0.86
		(0.47)	(0.50)	(0.47)	(0.41)	(0.37)	(0.35)
BingoUnknown	108	0.34	0.44	0.50	0.59	0.64	0.63
		(0.47)	(0.50)	(0.50)	(0.49)	(0.48)	(0.48)
All	300	0.33	0.51	0.59	0.70	0.74	0.76
		(0.47)	(0.50)	(0.49)	(0.46)	(0.44)	(0.43)

*Note*: Standard deviations in parentheses.

**Table A3:** Study 2A: Average Probability Participants Assigned to Repetition of Terminal Streaks, by Streak Length and Condition

		Average Probability (Streak Will Repeat)					
Condition	N	Streak = 2	Streak = 3	Streak = 4	Streak = 5	Streak = 6	Streak = 7
Analyst50	52	48.06	51.98	53.10	57.21	58.33	63.73
		(18.15)	(22.04)	(24.15)	(27.51)	(28.65)	(30.62)
Stock50	48	45.02	49.33	60.31	59.75	61.65	69.52
		(19.43)	(21.75)	(24.67)	(29.93)	(31.25)	(31.75)
Bingo50	56	42.54	44.39	46.30	43.25	43.27	45.54
		(20.36)	(19.51)	(26.97)	(29.32)	(26.91)	(32.09)
All	156	45.14	48.44	52.88	52.98	53.94	58.98
		(19.37)	(21.18)	(25.83)	(29.67)	(29.82)	(32.97)

*Note*: Standard deviations in parentheses.

**Table A4:** Study 2B: Proportion of Participants Predicting Repetition of Terminal Streaks, by Streak Length and Condition

		Proportion Who Predicted Streak Will Repeat					
Condition	N	Streak = 2	Streak = 3	Streak = 4	Streak = 5	Streak = 6	Streak = 7
Analyst50	97	0.23	0.26	0.34	0.52	0.50	0.52
		(0.42)	(0.44)	(0.47)	(0.50)	(0.50)	(0.50)
Stock50	103	0.26	0.27	0.41	0.47	0.53	0.53
		(0.44)	(0.44)	(0.49)	(0.50)	(0.50)	(0.50)
Bingo50	101	0.31	0.20	0.21	0.25	0.27	0.32
		(0.46)	(0.40)	(0.41)	(0.43)	(0.44)	(0.47)
All	301	0.27	0.24	0.32	0.41	0.43	0.46
		(0.44)	(0.43)	(0.47)	(0.50)	(0.50)	(0.50)

*Note*: Standard deviations in parentheses.

**Table A5:** Study 3A: Average Probability Participants Assigned to Repetition of Terminal Streaks, by Streak Length and Condition

		Average Probability (Streak Will Repeat)					
Condition	N	Streak = 2	Streak = 3	Streak = 4	Streak = 5	Streak = 6	Streak = 7
Analyst25‐50‐75	50	50.50	62.32	72.26	81.98	81.16	86.18
		(17.46)	(18.03)	(19.13)	(18.30)	(20.04)	(13.12)
Stock25‐50‐75	50	51.80	53.90	62.28	72.40	74.14	76.66
		(22.95)	(24.25)	(27.15)	(24.11)	(24.32)	(25.44)
Bingo25‐50‐75	50	44.70	57.06	66.68	71.08	74.38	76.34
		(18.65)	(18.49)	(22.27)	(22.06)	(24.32)	(25.57)
All	150	49.00	57.76	67.07	75.15	76.56	79.73
		(19.94)	(20.61)	(23.30)	(22.02)	(22.48)	(22.48)

*Note*: Standard deviations in parentheses.

**Table A6:** Study 3B: Proportion of Participants Predicting Repetition of Terminal Streaks, by Streak Length and Condition

		Proportion Who Predicted Streak Will Repeat					
Condition	N	Streak = 2	Streak = 3	Streak = 4	Streak = 5	Streak = 6	Streak = 7
Analyst25‐50‐75	98	0.27	0.42	0.53	0.50	0.60	0.55
		(0.44)	(0.49)	(0.50)	(0.50)	(0.49)	(0.50)
Stock25‐50‐75	93	0.28	0.52	0.54	0.63	0.58	0.63
		(0.45)	(0.50)	(0.50)	(0.48)	(0.49)	(0.48)
Bingo25‐50‐75	109	0.34	0.52	0.55	0.53	0.48	0.55
		(0.47)	(0.50)	(0.50)	(0.50)	(0.50)	(0.50)
All	300	0.30	0.49	0.54	0.55	0.55	0.58
		(0.46)	(0.50)	(0.50)	(0.50)	(0.50)	(0.50)

*Note*: Standard errors in parentheses.

## Appendix B Histograms of Individual Participants’ Prediction Strategies

Figures B1, B3, and B5 present the distribution of fitted slopes for individual participants’ predictions over the target sequences (reported on continuous probability scales in Studies 1A, 2A, and 3A). Figures B2, B4, and B6 present distributions of the percent‐change in the odds individual participants predict “repeat” for each unit increase in Streak Length (reported as a dichotomous choice in Studies 1B, 2B, and 3B). See text in the respective Results sections for discussions of these data.

## Appendix C Verbal Reports of Strategies

In this section, we report on participants’ verbal descriptions of their prediction strategies, which were collected at the end of the experimental sessions (“What was your strategy for predicting what would happen next? What information did you use to make your prediction?”). Two independent raters (Rater #1 and Rater #2) classified each participant's description by assigning a single category according to the instructions (Table C2 at the end of this section). Raters were blind to our hypotheses, and to the Study/Condition to which each participant was assigned. Two questions were raised by the raters after they began their task. These questions, and the responses provided to raters, are presented in Table C3 at the end of this section.

**Table C1:** Summary of Verbal Reports

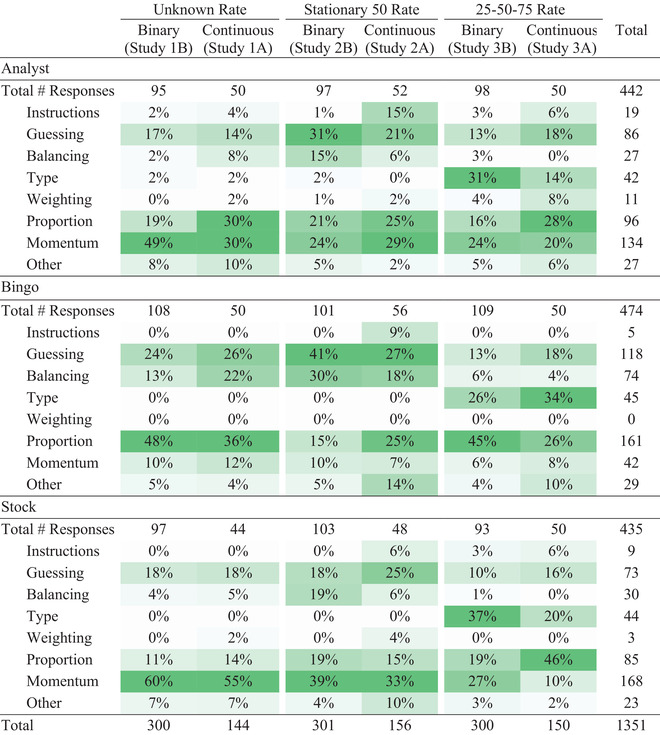

*Note*: Summary of verbal reports, Studies 1–3. Proportion of participant strategies assigned to each category, by Condition, Response Type, and Rate. Each count represented as a proportion of the total number of responses within a given Condition, Response Type, and Rate. Darker green cells indicate the more popular categories within a given Condition–Response Type–Rate section. Lighter green cells indicate less popular categories.

**Table C2:** Instructions Provided to Raters

Category	Description
Balancing	Participant mentioned something related to balancing out the number of outcome A (versus B) outcomes. For example, they chose A‐type (B‐type) to compensate for too few A‐type (B‐type) outcomes, or they chose B‐type (A‐type) to compensate for too many A‐type (B‐type) outcomes. General comments about having “too many” or “too few” of A‐type (B‐type) outcomes. Any reference to the number of Red or Blue balls remaining, e.g. “they took out 7 red balls, so there were only 43 red balls left.”
Guessing	Participant mentioned something about their “gut” reaction, their emotions, feeling or sensing that an outcome would occur, or simply guessing.
Proportion	Participant mentioned something about estimating the proportion of A‐type (B‐type) outcomes, or the ratio of A‐type to B‐type outcomes. Comments related to “counting the number of [Red, Up] or [Blue, Down] outcomes,” and choosing based on the number of each outcome. Any comments about basing their prediction on the relationship between A‐type and B‐type outcomes.
Momentum	Participant mentioned something about a trend, or change in the proportion of A‐type to B‐type outcomes over time. For example, participant says something like, “the company seems like it is on a roll, so the share price will probably continue to increase,” or “the analyst has been doing really well, so his book will probably increase in value.” Any comments related to momentum, increasing skill, increasing performance, learning, improving. Any comments related to deceleration, decreasing skill or effort, decreasing performance, something bad happening that changed the probability of a good outcome.
Instructions	Participant quotes the task instructions. For example, “the instructions said the rate was always 50%, so I always chose 50%.”
Weighting	Participant mentions some sort of weighted calculation. For example, “there were three types of companies, so I thought about the three success rates,” “only a good or average analyst could have so many successes, so I picked a rate halfway in between the good and average rates,” “if it looked like it could have been from the cage with 25 or 50 red balls, I guessed what might come next from either of those cages.”
Type	Participant talks about the “type” of analyst, company, stock, investor, bingo cage. For example, “I tried to figure out which type of analyst it was, and predicted based on the most likely type,” or “If it looked like it was a good company, I guessed what would happen next for a good company, if it looked bad, I guessed what would happen next for a bad company,” or “I tried to figure out which cage the draws were from, and guess what would come next from that cage.”
Other	Anything that cannot be classified into one of the above categories.

**Table C3:** Q&A Provided to Raters

**Question**: There are a lot of responses that vaguely talk about ‘I looked at the pattern’ or ‘I predicted the probability of the next one’ with no real specifics that fall into any one category. Would responses like these fall into that ‘other’ category? **Answer**: For things like “I looked at the pattern” I would generally go with either the “Proportion” or the “Momentum” buckets. If they mention anything about what happened “at the end” or “changes” then it should go in the “Momentum” bucket. Otherwise, you could randomize your categorization between “Proportion” and “Momentum” and that will still probably give us a good enough sense of what's going on. For things like “I predicted the probability of the next one” I would probably go with the “Guessing” bucket. But, whenever either of the above suggestions really feel too “forced” to you, feel free to use the “Other” bucket.
**Question**: If there is a combination of types, would that fall under the ‘other’ category? For example, someone might respond saying they ‘tried to follow the trend and made a prediction based on probability when they could, but occasionally went with their gut.’ **Answer**: If the participant mentions two strategies with no preference “Sometimes I did x, other times I did y,” record the first one they mention. If they indicate a preference “I tried to do x as much as I could, but when I could not do x I did y,” record the one they said they were “trying” to do.
**TIMING**: Try to limit yourself to less than 30 seconds for each response.

Participants’ responses were classified into one of 8 categories: (1) balancing outcomes; (2) guessing; (3) estimating a proportion or counting outcomes, including references to updating estimates; (4) momentum or increasing probability of one outcome over the other; (5) “following instructions” (often reported to justify an answer of 50% in Studies 2A and 2B); (6) deciding which “type” of generator produced the sequence, particularly with reference to the distribution of rates provided in Studies 3A and 3B (e.g. high‐ versus low‐performing analysts); (7) performing some sort of weighting calculation that takes into account the different “types” of generators, particularly in Studies 3A and 3B; 8) “other” unclassifiable responses.

Raters #1 and #2 only agreed on 58% of their ratings (781 out of 1,351). We measured inter‐rater reliability using Cohen's kappa, and obtained a kappa value of 0.40, which indicates “fair” agreement. A third independent rater (Rater #3) was asked to resolve the disagreements between Raters #1 and #2. Rater #3 was given only the 570 responses to which Raters #1 and #2 assigned conflicting categories, and was provided the same instructions and FAQs. Rater #3 was told to read each participant's response, and to decide whether Rater #1 or Rater #2's category was a better fit, given the instructions. Therefore, Rater #3 was *not* independently assigning categories to each response; rather, Rater #3 was restricted to the two categories previously assigned by Raters #1 and #2, and selected which of these two categories was a better match to the response. Rater #3 did not know the identity of Rater #1 or #2, was blind to our hypotheses, and to the Study/Condition to which each participant was assigned.

Table C1 presents a heatmap of the combined ratings that include Rater #3's resolutions. Darker green cells indicate that a category was assigned more often within a given Condition (Analyst, Bingo, Stock), Response Type (Continuous, Binary), and Rate (Unknown, Stationary .50, and Specified Distribution .25–.50–.75). There was considerable variety in these reports in every experimental Condition, but we see references to updating proportions comprise more than 50% of the responses for all experimental Conditions (categories labeled “Proportion” and “Momentum” in Table C1). This fits our hypothesis that base rate (proportion) updating is the primary inference process underlying the pervasive positive recency prediction patterns in our experiments.

We also see that “balancing” reports are scattered across experimental conditions and occur at highest rates in the Bingo Condition (30% and 18% for the Stationary .50 base rate in Studies 2A and 2B). This is consistent with our conclusion that Law of Small Numbers (“balancing out”) reasoning is likeliest to occur for random devices. Unsurprisingly, self‐reports of reasoning about “types” of generators (coding categories labeled “Type” and “Weighting”) are common when “types” (e.g. Bad, Average, and Good skill levels) are mentioned explicitly in the experimental instructions, as in the 25‐50‐75 Base Rate Studies (3A and 3B).

## References

[cogs13211-bib-0001] Alter, A. L. , & Oppenheimer, D. M. (2006). From a fixation on sports to an exploration of mechanism: The past, present, and future of hot hand research. Thinking and Reasoning, 12, 431–444.

[cogs13211-bib-0002] Altmann, E. M. , & Burns, B. D. (2005). Streak biases in decision making: Data and a memory model. Cognitive Systems Research, 6(1), 5–16.

[cogs13211-bib-0003] Amir, G. S. , & Williams, J. S. (1999). Cultural influences on children's probabilistic thinking. The Journal of Mathematical Behavior, 18(1), 85–107.

[cogs13211-bib-0004] Anderson, N. H. (1960). Effect of first‐order conditional probability in a two‐choice learning situation. Journal of Experimental Psychology, 59, 73–93. 13793434 10.1037/h0049023

[cogs13211-bib-0005] Anderson, M. J. , & Sunder, S. (1995). Professional traders as intuitive Bayesians. Organizational Behavior and Human Decision Processes, 64(2), 185–202.

[cogs13211-bib-0006] Asparouhova, E. , Hertzel, M. , & Lemmon, M. (2009). Inference from streaks in random outcomes: Experimental evidence on beliefs in regime shifting and the law of small numbers. Management Science, 55(1), 1766–1782.

[cogs13211-bib-0007] Avugos, S. , Bar‐Eli, M. , Ritov, I. , & Sher, E. (2013a). The elusive reality of efficacy– performance cycles in basketball shooting: an analysis of players’ performance under invariant conditions. International Journal of Sport and Exercise Psychology, 11(2), 184–202.

[cogs13211-bib-0008] Ayton, P. , Hunt, A. J. , & Wright, G. (1989). Psychological conceptions of randomness. Journal of Behavioral Decision Making, 2(4), 221–238.

[cogs13211-bib-0009] Ayton, P. , & Fischer, I. (2004). The hot hand fallacy and the gambler's fallacy: Two faces of subjective randomness? Memory & Cognition, 32(8), 1369–1378. 15900930 10.3758/bf03206327

[cogs13211-bib-0010] Baquero, G. , & Verbeek, M. (2015). Hedge fund flows and performance streaks: How investors weigh information (No. ESMT‐15‐01). ESMT European School of Management and Technology.

[cogs13211-bib-0011] Bar‐Eli, M. , Avugos, S. , & Raab, M. (2006). Twenty years of “hot hand” research: Review and critique. Psychology of Sport and Exercise, 7(6), 525–553.

[cogs13211-bib-0012] Bar‐Hillel, M. , & Wagenaar, W. A. (1991). The perception of randomness. Advances in Applied Mathematics, 12, 428–454.

[cogs13211-bib-0013] Barberis, N. , Shleifer, A. , & Vishny, R. (1998). A model of investor sentiment. Journal of Financial Economics, 49, 307–343.

[cogs13211-bib-0014] Barron, G. , & Leider, S. (2010). The role of experience in the gambler's fallacy. Journal of Behavioral Decision Making, 23(1), 117–129.

[cogs13211-bib-0015] Batanero, C. , Chernoff, E. J. , Engel, J. , Lee, H. S. , & Sánchez, E. (2016). Research on teaching and learning probability. In Research on teaching and learning probability (pp. 1–33). Springer, Cham.

[cogs13211-bib-0016] Blinder, D. S. , & Oppenheimer, D. M. (2008). Beliefs about what types of mechanisms produce random sequences. Journal of Behavioral Decision Making, 21(4), 414–427.

[cogs13211-bib-0017] Bloomfield, R. , & Hales, J. (2002). Predicting the next step of a random walk: experimental evidence of regime‐shifting beliefs. Journal of Financial Economics, 65(3), 397–414.

[cogs13211-bib-0018] Bogartz, R. S. (1965). Sequential dependencies in children's probability learning. Journal of Experimental Psychology, 70(4), 365–370. 5826023 10.1037/h0022372

[cogs13211-bib-0112] Borovnik, M. , and Peard, R. (1996). Probability. In A.J. Bishop (Ed.), International Handbook of Mathematics Education (239‐287). Netherlands: Kluwer Academic Publishers.

[cogs13211-bib-0019] Boynton, D. M. (2003). Superstitious responding and frequency matching in the positive bias and gambler's fallacy effects. Organizational Behavior and Human Decision Processes, 91, 119–127.

[cogs13211-bib-0020] Braga, J. N. , Ferreira, M. B. , Sherman, S. J. , Mata, A. , Jacinto, S. , & Ferreira, M. (2018). What's next? Disentangling availability from representativeness using binary decision tasks. Journal of Experimental Social Psychology, 76, 307–319.

[cogs13211-bib-0021] Bulkley, G. , & Harris, R. D. F. (1997). Irrational analysts’ expectations as a cause of excess volatility in stock prices. Royal Economic Society, 107(441), 359–371.

[cogs13211-bib-0022] Burns, B. D. (2002). Heuristics as beliefs and as behaviors: The adaptiveness of the “hot hand.” Cognitive Psychology, 48(3), 295–331. 10.1016/j.cogpsych.2003.07.003 15020214

[cogs13211-bib-0023] Burns, B. D. (2003). When it is adaptive to follow streaks: Variability and stocks. In Proceedings of the Annual Meeting of the Cognitive Science Society (Vol. 25, No. 25).

[cogs13211-bib-0024] Burns, B. D. , & Corpus, B. (2004). Randomness and inductions from streaks: “Gambler's fallacy” versus” hot hand.” Psychonomic Bulletin & Review, 11(1), 179–184. 15117006 10.3758/bf03206480

[cogs13211-bib-0025] Caruso, E. M. , Waytz, A. , & Epley, N. (2010). The intentional mind and the hot hand: Perceiving intentions makes streaks seem likely to continue. Cognition, 116(1), 149–153. 20472229 10.1016/j.cognition.2010.04.006

[cogs13211-bib-0026] Chen, D. L. , Schonger, M. , & Wickens, C. (2016). oTree ‐ An open‐source platform for laboratory, online and field experiments. Journal of Behavioral and Experimental Finance, 9, 88–97.

[cogs13211-bib-0027] Chiesi, F. , & Primi, C. (2009). Recency effects in primary‐age children and college students. International Electronic Journal of Mathematics Education, 4(3), 259–279.

[cogs13211-bib-0028] Cohen, J. (1988). Statistical power analysis for the behavioral sciences (2nd ed.). Hillsdale, NJ: Erlbaum.

[cogs13211-bib-0029] Conrad, J. , & Kaul, G. (1998). An anatomy of trading strategies. The Review of Financial Studies, 11(3), 489–519.

[cogs13211-bib-0030] Craig, G. J. , & Meyers, J. L. (1963). A developmental study of sequential two‐choice decision‐ making. Child Development, 34, 483–493. 14023622 10.1111/j.1467-8624.1963.tb05153.x

[cogs13211-bib-0031] Croson, R. , & Sundali, J. (2005). The gambler's fallacy and the hot hand: Empirical data from casinos. Journal of Risk and Uncertainty, 30(3), 195–209.

[cogs13211-bib-0032] De Bondt, W. F. M. (1991). What do economists know about the stock market? Journal of Portfolio Management, 17(2), 84–91.

[cogs13211-bib-0033] De Bondt, W. F. M. (1993). Betting on trends: Intuitive forecasts of financial risk and return. International Journal of Forecasting, 9, 355–371.

[cogs13211-bib-0034] De Bondt, W. F. , & Thaler, R. H. (1989). Anomalies: A mean‐reverting walk down Wall Street. Journal of Economic Perspectives, 3(1), 189–202.

[cogs13211-bib-0035] De Bondt, W. F. M. , & Thaler, R. H. (1990). Do security analysts overreact? The American Economic Review, 80(2), 52–57.

[cogs13211-bib-0036] Derks, P. L. , & Paclisanu, M. I. (1967). Simple strategies in binary prediction by children and adults. Journal of Experimental Psychology, 73(2), 278.

[cogs13211-bib-0037] Diener, D. , & Thompson, W.B. (1985). Recognizing randomness. The American Journal of Psychology, 98(3), 433–447.

[cogs13211-bib-0038] Dohmen, T. , Falk, A. , Huffman, D. , Marklein, F. , & Sunde, U. (2009). Biased probability judgment: Evidence of incidence and relationship to economic outcomes from a representative sample. Journal of Economic Behavior & Organization, 72(3), 903–915.

[cogs13211-bib-0039] Durham, G. R. , Hertzel, M. G. , & Martin, J. S. (2005). The market impact of trends and sequences in performance: New evidence. The Journal of Finance, 60(5), 2551–2569.

[cogs13211-bib-0040] Edwards, W. (1961). Probability learning in 1,001 trials. Journal of Experimental Psychology, 62, pp. 381–390. 10.1037/h0041970 13889318

[cogs13211-bib-0041] Estes, W. K. (1962). Learning theory. Annual Review of Psychology, 13, 107–144. 10.1146/annurev.ps.13.020162.000543 13890856

[cogs13211-bib-0042] Estes, W. K. (1964). Probability learning. In A.W. Melton (Ed.), Categories of Human Learning (pp. 88–128). New York: Academic Press.

[cogs13211-bib-0043] Falk, R. (1981). The perception of randomness. In Proceedings of the Fifth International Conference for the Psychology of Mathematics Education (pp. 222–229). Grenoble, France.

[cogs13211-bib-0044] Farmer, G.D. , Warren, P.A. , & Hahn, U. (2017). Who “believes” in the Gambler's Fallacy and why? Journal of Experimental Psychology: General, 146(1 *)*, 63–76. 28054813 10.1037/xge0000245 PMC5215234

[cogs13211-bib-0045] Feller, W. (1968). An Introduction to Probability Theory and its Applications (3rd Edition, Volume 1). New York: John Wiley.

[cogs13211-bib-0046] Fiorina, M.P. (1971). A note on probability matching and rational choice. Behavioral Science, 16(2 *)*, 158–166.

[cogs13211-bib-0047] Fischbein, E. (1975). The intuitive sources of probabilistic thinking in children. Dordrecht, The Netherlands: Reidel.

[cogs13211-bib-0048] Fischbein, E. , & Schnarch, D. (1997). The evolution with age of probabilistic, intuitively based misconceptions. Journal for Research in Mathematics Education, 28, 96–105.

[cogs13211-bib-0049] Fischer, I. , & Savranevski, L. (2015). Extending the two faces of subjective randomness: From the gambler's and hot‐hand fallacies toward a hierarchy of binary sequence perception. Memory & cognition, 43(7), 1056–1070. 26044942 10.3758/s13421-015-0523-5

[cogs13211-bib-0050] Forbes, W. P. (1995). Picking winners? A survey of the mean reversion and overreaction of stock prices literature. Journal of Economic Surveys, 10(2), 123–158.

[cogs13211-bib-0051] Gilovich, T. , Vallone, R. , & Tversky, A. (1985). The hot hand in basketball: On the misperception of random sequences. Cognitive Psychology, 17(3), 295–314.

[cogs13211-bib-0052] Green, S. B. (1991). How many subjects does it take to do a regression analysis? Multivariate Behavioral Research, 26, 499‐510. 26776715 10.1207/s15327906mbr2603_7

[cogs13211-bib-0053] Gronchi, G. , & Sloman, S. A. (2008). Do causal beliefs influence the hot‐hand and the gambler's fallacy? In Proceedings of the 30th annual conference of the cognitive science society (pp. 1164–1168). Cognitive Science Society. Austin, TX.

[cogs13211-bib-0054] Hahn, U. , & Warren, P. A. (2009). Perceptions of randomness: why three heads are better than four. Psychological Review, 116(2), 454. 19348550 10.1037/a0015241

[cogs13211-bib-0055] Hammond, K. R. , Hamm, R. M. , Grassia, J. , & Pearson, T. (1987). Direct comparison of the efficacy of intuitive and analytical cognition in expert judgment. IEEE Transactions on systems, man, and cybernetics, 17(5), 753–770.

[cogs13211-bib-0115] Harradine, A. , Batanero, C. , Rossman, A. (2011). Students and Teachers’ Knowledge of Sampling and Inference. In C. Batanero , G. Burrill , & C. Readinged (Eds.), Teaching Statistics in School Mathematics‐Challenges for Teaching and Teacher Education. New ICMI Study Series, vol 14. Springer, Dordrecht.

[cogs13211-bib-0057] Harris, R. J. (1985). A primer of multivariate statistics (2nd ed.). New York: Academic Press.

[cogs13211-bib-0058] Hawkins, A. S. , & Kapadia, R. (1984). Children's conceptions of probability—a psychological and pedagogical review. Educational Studies in Mathematics, 15(4), 349–377.

[cogs13211-bib-0059] Heinze, G. and Schemper, M. (2002). A solution to the problem of separation in logistic regression. Statistics in Medicine, 21(16), 2409–2419. 12210625 10.1002/sim.1047

[cogs13211-bib-0060] Jarvik, M. E. (1951). Probability learning and a negative recency effect in the serial anticipation of alternative symbols. Journal of Experimental Psychology, 41(4), 291–297. 14850645 10.1037/h0056878

[cogs13211-bib-0061] Jegadeesh, N. , & Titman, S. (2011). Momentum. Annual Review of Financial Economics, 3(1), 493–509.

[cogs13211-bib-0062] Johnson, J. , Tellis, G. J. , & Macinnis, D. J. (2005). Losers, winners, and biased trades. Journal of Consumer Research, 32, 324–329.

[cogs13211-bib-0063] Jones, D. L. (2004). Probability in middle grades mathematics textbooks: An examination of historical trends, 1957–2004. University of Missouri‐Columbia.

[cogs13211-bib-0064] Kahneman, D. , & Tversky, A. (1973). On the psychology of prediction. Psychological Review, 80(4), 237–251.

[cogs13211-bib-0065] Kareev, Y. (2001). Seven (indeed, plus or minus two) and the detection of correlations. Psychological Review, 107(2), 397. 10.1037/0033-295x.107.2.397 10789204

[cogs13211-bib-0066] Koehler, J. J. , & Conley, C. A. (2003). The “hot hand” myth in professional basketball. Journal of Sport and Exercise Psychology, 25(2), 253–259.

[cogs13211-bib-0067] Konold, C. (1995). Confessions of a coin flipper and would‐be instructor. The American Statistician, 49(2), 203–209.

[cogs13211-bib-0068] Laplace, P. S. (1902). A philosophical essay on probabilities ( F. W. Truscott & F. L. Emory , Translators). New York, NY: John Wiley & Sons. (Original work published 1814).

[cogs13211-bib-0069] Lecoutre, M. P. (1992). Cognitive models and problem spaces in “purely random” situations. Educational Studies in Mathematics, 23(6), 557–568.

[cogs13211-bib-0070] Lee, W. (1971). Decision Theory and Human Behavior (pp. 163–167). New York: John Wiley & Sons.

[cogs13211-bib-0071] Lindman, H. , & Edwards, W. (1961). Supplementary report: Unlearning the gambler's fallacy. Journal of Experimental Psychology, 62(6), 630. 14465494 10.1037/h0046635

[cogs13211-bib-0072] Loh, R. K. , & Warachka, M. (2012). Streaks in earnings surprises and the cross‐section of stock returns. Management Science, 58(7), 1305–1321.

[cogs13211-bib-0073] Lopes, L. L. (1982). Doing the impossible: A note on induction and the experience of randomness. Journal of Experimental Psychology: Learning, Memory and Cognition, 8, 626–636.

[cogs13211-bib-0074] Lopes, L. L. , & Oden, G. C. (1987). Distinguishing between random and nonrandom events. Journal of Experimental Psychology: Learning, Memory and Cognition, 13(3), 392–400.

[cogs13211-bib-0075] Mason, W. , & Suri, S. (2012). Conducting behavioral research on Amazon's Mechanical Turk. Behavior Research Methods, 44(1), 1–23. 21717266 10.3758/s13428-011-0124-6

[cogs13211-bib-0113] Meletiou‐Mavrotheris, M. (2007). The formalist mathematical tradition as an obstacle to stochastical reasoning. In Philosophical dimensions in mathematics education (pp. 131‐155). Springer, Boston, MA.

[cogs13211-bib-0076] Militana, E. , Wolfson, E. , & Cleaveland, J. M. (2010). An effect of inter‐trial duration on the gambler's fallacy choice bias. Behavioural Processes, 84(1), 455–459. 20176090 10.1016/j.beproc.2010.02.010

[cogs13211-bib-0077] Miller, J. B. , & Sanjurjo, A. (2018a). How experience confirms the gambler's fallacy when sample size is neglected. OSF Preprints, 30.

[cogs13211-bib-0078] Miller, J. B. , & Sanjurjo, A. (2018b). Surprised by the hot hand fallacy? A truth in the law of small numbers. Econometrica, 86(6), 2019–2047.

[cogs13211-bib-0079] Morrison, R.S. , & Ordeshook, P.C. (1975). Rational choice, light guessing and the gambler's fallacy. Public Choice, 22(1 *)*, 79–89.

[cogs13211-bib-0080] Morsanyi, K. , Handley, S. J. , & Serpell, S. (2013). Making heads or tails of probability: An experiment with random generators. British Journal of Educational Psychology, 83, 379–395. 23822527 10.1111/j.2044-8279.2012.02067.x

[cogs13211-bib-0081] Murphy, G.L. , & Ross, B.H. (2010). Uncertainty in category‐based induction: When do people integrate across categories? Journal of Experimental Psychology: Learning, Memory, and Cognition, 36(2 *)*, 263–276. 20192530 10.1037/a0018685 PMC2856341

[cogs13211-bib-0082] Nickerson, R. S. (2002). The production and perception of randomness. Psychological Review, 109, 330–357. 11990321 10.1037/0033-295x.109.2.330

[cogs13211-bib-0083] Nicks, D. C. (1959). Prediction of sequential two‐choice decisions from event runs. Journal of Experimental Psychology, 57(2), 105. 13641581 10.1037/h0045193

[cogs13211-bib-0084] Nisbett, R.E. , & Wilson, T.D. (1977). Telling more than we can know: Verbal reports on mental processes. Psychological Review, 84(3 *)*, 231–259.

[cogs13211-bib-0085] Olivola, C. Y. , & Oppenheimer, D. M. (2008). Randomness in retrospect: Exploring the interactions between memory and randomness cognition. Psychonomic Bulletin & Review, 15(5), 991–996. 18926994 10.3758/PBR.15.5.991

[cogs13211-bib-0086] Önkal, D. , & Muradoglu, G. (1996). Effects of task format on probabilistic forecasting of stock prices. International Journal of Forecasting, 12(1), 9–24.

[cogs13211-bib-0087] Oskarsson, A. T. , Boven, L. V. , McClelland, G. H. , & Hastie, R. (2009). What's next? Judging sequences of binary events. Psychological Bulletin, 135, 262–285. 19254080 10.1037/a0014821

[cogs13211-bib-0088] Rabin, M. (2002). Inference by believers in the law of small numbers. The Quarterly Journal of Economics, 117(3), 775–816.

[cogs13211-bib-0089] Rabin, M. , & Vayanos, D. (2010). The gambler's and hot‐hand fallacies: Theory and applications. The Review of Economic Studies, 77(2), 730–778.

[cogs13211-bib-0090] Rapoport, A. , & Budescu, D. V. (1997). Randomization in individual choice behavior. Psychological Review, 104(3), 603.

[cogs13211-bib-0091] Rao, J. M. (2009). Experts’ perceptions of autocorrelation: The hot hand fallacy among professional basketball players. Unpublished technical manuscript. California. San Diego. Downloaded from http://www.justinmrao.com/playersbeliefs.pdf (July 11th, 2012).

[cogs13211-bib-0092] Reimers, S. , Donkin, C. , Le Pelley, M. E. (2018). Perceptions of randomness in binary sequences: Normative, heuristic, or both? Cognition, 172, 11–25. 29202364 10.1016/j.cognition.2017.11.002

[cogs13211-bib-0093] Restle, F. (1961). The Psychology of Judgment and Choice: A Theoretical Essay. New York: Wiley & Sons.

[cogs13211-bib-0094] Restle, F. (1966). Run structure and probability learning: Disproof of Restle's model. Journal of Experimental Psychology, 72(3), 382–389. 5968684 10.1037/h0023637

[cogs13211-bib-0095] Roney, C. J. , & Trick, L. M. (2009). Sympathetic magic and perceptions of randomness: The hot hand versus the gambler's fallacy. Thinking & reasoning, 15(2), 197–210.

[cogs13211-bib-0096] Shaughnessy, J. M. (1992). Research in probability and statistics: Reflections and directions. In D. A. Grouws (Ed.), Handbook o f research on mathematics teaching and learning (pp. 465–494). Reston, VA: National Council of Teachers of Mathematics.

[cogs13211-bib-0097] Shaughnessy, J. M. , Canada, D. , & Ciancetta, M. (2003). Middle school students' thinking about variability in repeated trials: A cross‐task comparison. In N. A. Pateman , B. J. Dougherty & J. T. Zilliox (Eds.), Proceedings o f the 2003 Joint Meeting of PME and PMENA (Vol. 4, pp. 159–165). Honolulu, HI: University of Hawai'i.

[cogs13211-bib-0098] Scholl, S. , & Greifeneder, R. (2011). Disentangling the effects of alternation rate and maximum run length on judgments of randomness. Judgment and Decision Making, 6(6), 531–541.

[cogs13211-bib-0099] Shanthikumar, D. M. (2012). Consecutive earnings surprises: Small and large trader reactions. The Accounting Review, 87(5), 1709–1736.

[cogs13211-bib-0100] Soetens, E. , Boer, L. C. , & Hueting, J. E. (1985). Expectancy or automatic facilitation? Separating sequential effects in two‐choice reaction time. Journal of Experimental Psychology: Human Perception and Performance, 11(5), 598.

[cogs13211-bib-0101] Steinbring, H. (1990). The use of chance‐concept in everyday teaching – Aspects of socially constituted epistemology of mathematical knowledge. In J. B. Garfield (Ed.), Research papers from the Third International Conference on Teaching Statistics . University of Otago, Dunedin, New Zealand.

[cogs13211-bib-0102] Stohl, H. (2005). Probability in teacher education and development. In Exploring probability in school (pp. 345–366). Springer, Boston, MA.

[cogs13211-bib-0103] Studer, B. , Limbrick‐Oldfield, E.H. , & Clark, L. (2015). “Put your money where your mouth is”: Effects of streaks on confidence and betting in a binary choice task. Journal of Behavioral Decision Making, 28(3), 239–249. 26236092 10.1002/bdm.1844 PMC4515090

[cogs13211-bib-0104] Suetens, S. , Galbo‐Jorgensen, C.B. , & Tyran, J.‐R. (2016). Predicting Lotto numbers: A natural experiment on the Gambler's Fallacy and the Hot‐Hand Fallacy. Journal of the European Economic Association, 14(3 *)*, 584–607.

[cogs13211-bib-0105] Sun, Y. , & Wang, H. (2010). Gambler's fallacy, hot hand belief, and the time of patterns. Judgment and Decision Making, 5(2), 124–132.

[cogs13211-bib-0106] Turk‐Browne, N.B. , Scholl, B.J. , Chun, M.M. , & Johnson, M.K. (2009). Neural evidence of statistical learning: Efficient detection of visual regularities without awareness. Journal of Cognitive Neuroscience, 21(10 *)*, 1934–1945. 18823241 10.1162/jocn.2009.21131 PMC2773825

[cogs13211-bib-0107] Tversky, A. , & Kahneman, D. (1971). Belief in the law of small numbers. Psychological bulletin, 76(2), 105.

[cogs13211-bib-0114] Tversky, A. , & Kahneman, D. (1983). Extensional versus intuitive reasoning: The conjunction fallacy in probability judgment. Psychological review, 90(4), 293.

[cogs13211-bib-0108] Tyszka, T. , Markiewicz, Ł. , Kubińska, E. , Gawryluk, K. , & Zielonka, P. (2017). A belief in trend reversal requires access to cognitive resources. Journal of Cognitive Psychology, 29(2), 202–216.

[cogs13211-bib-0109] Tyszka, T. , Zielonka, P. , Dacey, R. , & Sawicki, P. (2008). Perception of randomness and predicting uncertain events. Thinking & Reasoning, 14(1), 83–110.

[cogs13211-bib-0110] Vergin, R. C. (2001). Winning streaks in sports and the misperception of momentum. Journal of Sport Behavior, 23(2), 181.

[cogs13211-bib-0111] Windschitl, P.D. , & Wells, G.L. (1996). Measuring psychological uncertainty: Verbal versus numeric methods. Journal of Experimental Psychology: General, 2(4 *)*, 343–364.

