# Supplementary material for: Predicting Outcomes in a Sequence of Binary Events: Belief Updating and Gambler's Fallacy Reasoning
Source: Cogn Sci. 2023 Jan 21;47(1):e13211. doi: 10.1111/cogs.13211 (PMC10078382; doi:10.1111/cogs.13211)
Supplement: Supplementary file 1 — Supporting Material [file COGS-47-0-s001.docx]

**Predicting Outcomes in a Sequence of Binary Events:**

**Belief Updating and Gambler’s Fallacy Reasoning**

ONLINE SUPPLEMENT

Kariyushi Rao and Reid Hastie

Department of Behavioral Science, The University of Chicago Booth School of Business,

Chicago, IL, USA

September 21, 2022

Kariyushi Rao: https://orcid.org/0000-0002-5027-8233

© Kariyushi Rao and Reid Hastie, 2022

Correspondence should be addressed to Kariyushi Rao, Department of Behavioral Science, Chicago Booth School of Business, 5807 S Woodlawn Avenue, Chicago IL 60637. Email: Kariyushi.Rao@ChicagoBooth.edu.

**Table of Contents**

Introduction 3

Chapter 1: Stimulus Materials 3

Section 1.1: Description of stimulus sequences 3

Section 1.2: Method of Randomization 7

Chapter 2: Instructions and Procedure 8

Section 2.1: Instructions 8

Section 2.2: Comprehension Check Questions 13

Section 2.3: Prediction Prompts 14

Section 2.4: Screenshots of Experimental Interface 17

Chapter 3: Filler Sequences 20

Chapter 4: Binary Logistic Regression Analysis 33

Section 4.1: Explanation of the Binary Logistic Repeated Measures Model 33

Section 4.2: Study 1B 35

Section 4.3: Study 2B 36

Section 4.4: Study 3B 38

Chapter 5: Strategic Heterogeneity in Reversal versus Repetition Patterns 39

Section 5.1: Study 1A 39

Section 5.2: Study 1B 41

Section 5.3: Study 2A 43

Section 5.4: Study 2B 45

Section 5.5: Study 3A 47

Section 5.6: Study 3B 49

Chapter 6: Individual Differences 50

Section 6.1: Individual Difference Measures and Participant Strategies 51

Section 6.2: Interpretation of Binary Logistic Regression Coefficients 62

References 64

# Introduction

This supplement contains six Chapters. Chapter 1 provides an overview of the stimulus materials, including a description of the stimulus sequences and the method of randomization for selection and presentation of stimulus materials to participants. A detailed explanation of the experimental instructions and procedure can be found in Chapter 2, including: 1) transcripts of the instructions presented to participants in each Condition of each Study, 2) a description of the comprehension check questions participants were required to complete prior to the start of the procedure, and 3) screenshots of the experimental interface, including the prediction prompts presented to participants during the procedure. Graphic and numeric summaries of participants' response patterns for the Filler sequences (those ending in reversals) are presented in Chapter 3. Chapter 4 provides a brief tutorial for performing a binary logistic repeated measures analysis, which is the appropriate analytical model for the binary response Studies. The results of this analysis for Studies 1B, 2B, and 3B are also presented in this Chapter. Chapter 5 describes strategic heterogeneity observed in participants' patterns of response across all six Studies in the present research program. Chapter 6 concludes with a discussion of individual difference measures and participants' strategies.

# Chapter 1: Stimulus Materials

## Section 1.1: Description of stimulus sequences

The same stimulus sequences were used in all six of the present Studies. Though the images representing the outcomes in each sequence differed across Conditions in each Study, the patterns of outcomes were the same across Conditions. There are two pools of stimuli: Targets and Fillers. The Target stimuli pool contains 22 sequences, each comprised of 8 signals, and each ending in a streak of at least two identical signals. For each terminal Streak Length 2 through 6, four different sequences were created. Two sequences end in a streak of Red/Up outcomes, and two sequences end in a streak of Blue/Down outcomes. The sequences ending in a streak of Red/Up outcomes are mirror images of the sequences ending in a streak of Blue/Down outcomes. For terminal Streak Length 7, two sequences were created. One ends in 7 Red/Up outcomes, and the other ends in 7 Blue/Down outcomes.

The patterns of outcomes that precede the terminal streak in each Target sequence were designed to achieve the several goals: 1) achieve an average alternation rate close to 0.50 across all Target sequences, 2) include sequences having the same terminal Streak Length, but different alternation rates, 3) include sequences having the same terminal Streak Length, but different Bayesian posterior probabilities. The third goal is only relevant to Studies 3A and 3B. In those Studies, a distribution of possible base rates was provided to participants, which made it possible to estimate the posterior probability that each sequence’s terminal streak would repeat, conditional on the pattern of outcomes in that sequence and the distribution of possible rates. The full list of Target stimuli sequences is enumerated in Table OS1.

The Filler stimuli pool contains 24 sequences, each comprised of 8 signals, and each ending in a reversal (e.g., Red-Blue, Up-Down). The patterns of outcomes in each Filler sequence were designed to achieve several goals: 1) achieve an average alternation rate close to 0.50 across both Target and Filler sequences, 2) introduce streaks of various lengths that appear toward the beginning and middle of a given sequence, 3) achieve an average Bayesian posterior probability close to 0.50 across both Target and Filler sequences, 4) balance the total number of Red/Up signals and Blue/Down signals across all Target and Filler sequences. The full list of Filler stimuli sequences is enumerated in Table OS2.

**Table OS1**

*Description of Target Stimulus Sequences.*

| Sequence | Terminal Streak  Type | | Terminal Streak  Length | Proportion Streak Type | Alternation  Rate | Bayesian  Posterior |
| --- | --- | --- | --- | --- | --- | --- |
| 01001011 10110100 01010100 10101011 | 1  0  0  1 | | 2  2  2  2 | 0.50  0.50  0.63  0.63 | 0.71  0.71  0.86  0.86 | 0.50 0.50 0.60 0.60 |
| 01010111 10101000 01011000 10100111 | 1  0  0  1 | | 3  3  3  3 | 0.63  0.63  0.63  0.63 | 0.71  0.71  0.57  0.57 | 0.60 0.60 0.60 0.60 |
| 00101111 11010000 01010000 10101111 | 1  0  0  1 | | 4  4  4  4 | 0.63  0.63  0.75  0.75 | 0.43  0.43  0.57  0.57 | 0.60 0.60 0.68 0.68 |
| 00100000 11011111 01011111 10100000 | 0  1  1  0 | | 5  5  5  5 | 0.88  0.88  0.75  0.75 | 0.29  0.29  0.43  0.43 | 0.72 0.72 0.68 0.68 |
| 01000000 10111111 11000000 00111111 | 0  1  0  1 | | 6  6  6  6 | 0.88  0.88  0.75  0.75 | 0.29  0.29  0.14  0.14 | 0.72 0.72 0.68 0.68 |
| 01111111 10000000 | 1  0 | | 7  7 | 0.88  0.88 | 0.14  0.14 | 0.72 0.72 |
| **Mean** |  |  |  | **0.72** | **0.47** | **0.65** |

*Note:* Terminal Streak Type is the signal type repeated in the terminal streak at the end of each sequence (Coding: 1 = Up/Red, 0 = Down/Blue). Terminal Streak Length is the number of identical signals repeated at the end of each Target sequence. Proportion Streak Type is the proportion of signals in a given sequence that match the signal type repeated in the terminal streak. Bayesian Posterior applies to Studies 3A and 3B only. It is the posterior probability that a given terminal streak will repeat, conditional on the pattern of signals in the sequence, and the distribution of probability rates provided to participants: *p*(0.25) = *p*(0.50) = *p*(0.75) = 0.33.

**Table OS2**

*Description of Filler Stimulus Sequences.*

| Sequence | Longest Streak  Type | Longest Streak  Length | Proportion  Streak Type | Alternation  Rate | Bayesian Posterior |
| --- | --- | --- | --- | --- | --- |
| 01010101  10101010 | –  – | –  – | –  – | 1.00  1.00 | 0.50  0.50 |
| 00110101  00110110  01010010  01101010  10100110  11001101  11010010  11011001 | 1  1  0  1  1  1  0  0 | 2  2  2  2  2  2  2  2 | 0.50  0.50  0.63  0.50  0.50  0.63  0.50  0.38 | 0.71  0.57  0.86  0.86  0.71  0.57  0.71  0.57 | 0.50  0.50  0.60  0.50  0.50  0.60  0.50  0.60 |
| 01000101  01011101  01110010  10001010  11000110  11101010 | 0  1  1  0  0  1 | 3  3  3  3  3  3 | 0.63  0.63  0.50  0.63  0.50  0.63 | 0.71  0.71  0.57  0.71  0.43  0.71 | 0.40  0.60  0.50  0.60  0.50  0.40 |
| 00001101 01011110 10000101 11110010 | 0  1  0  1 | 4  4  4  4 | 0.63  0.63  0.63  0.63 | 0.43  0.57  0.57  0.43 | 0.40  0.40  0.40  0.40 |
| 01000001 11111010 | 0  1 | 5  5 | 0.75  0.75 | 0.43  0.43 | 0.32  0.32 |
| 10000001 11111101 | 0  1 | 6  6 | 0.75  0.88 | 0.29  0.29 | 0.32  0.72 |
| Mean |  |  | **0.59** | **0.62** | **0.48** |

*Note:* Longest Streak Type is the signal type repeated in the longest streak in each sequence (Coding: 1 = Up/Red, 0 = Down/Blue).  Longest Streak Length is the number of identical signals repeated in the longest streak in each sequence. For sequences with multiple “longest streaks” of length 2, we take the signal type of the streak closest to the end of the sequence. Proportion Streak Type is the proportion of signals in a given sequence that match the signal type repeated in the longest streak. Bayesian Posterior is the posterior probability that the final (8th) signal in the sequence will repeat, conditional on the pattern of signals in the sequence, and the distribution of probability rates in Studies 3A and 3B: *p*(0.25) = *p*(0.50) = *p*(0.75) = 0.33.

## Section 1.2: Method of Randomization

The same procedure for randomly selecting, and presenting, stimulus sequences was used in all six of the reported Studies. Each participant was shown 18 sequences – 12 Filler sequences and 6 Target sequences. For each participant, the 12 Filler sequences were randomly drawn from a pool of 24 sequences ending in reversals. The 6 focal Target stimuli were randomly selected from the pool of 22 sequences ending in streaks. One sequence was selected for each Streak Length 2 through 7. For each Streak Length 2 through 6, we prepared 4 different Target stimuli. For Streak Length 7, we prepared 2 different Target stimuli. For each Streak Length, half of the stimuli end in Red/Up streaks, and half end in Blue/Down streaks. In this way, we were able to alternate the signal type of the terminal streak, and introduce variation in the patterns preceding the terminal streak in each Target sequence. One of the 12 Filler sequences randomly selected for each participant is randomly selected to appear in the first round of the procedure. The remaining 11 Filler sequences, and 6 Target sequences, are shuffled and presented in random order across rounds 2-18. Figure OS1 illustrates the randomization process.

**Figure OS1**

*Illustration of Process Used to Randomly Select and Present Stimuli to Participants*


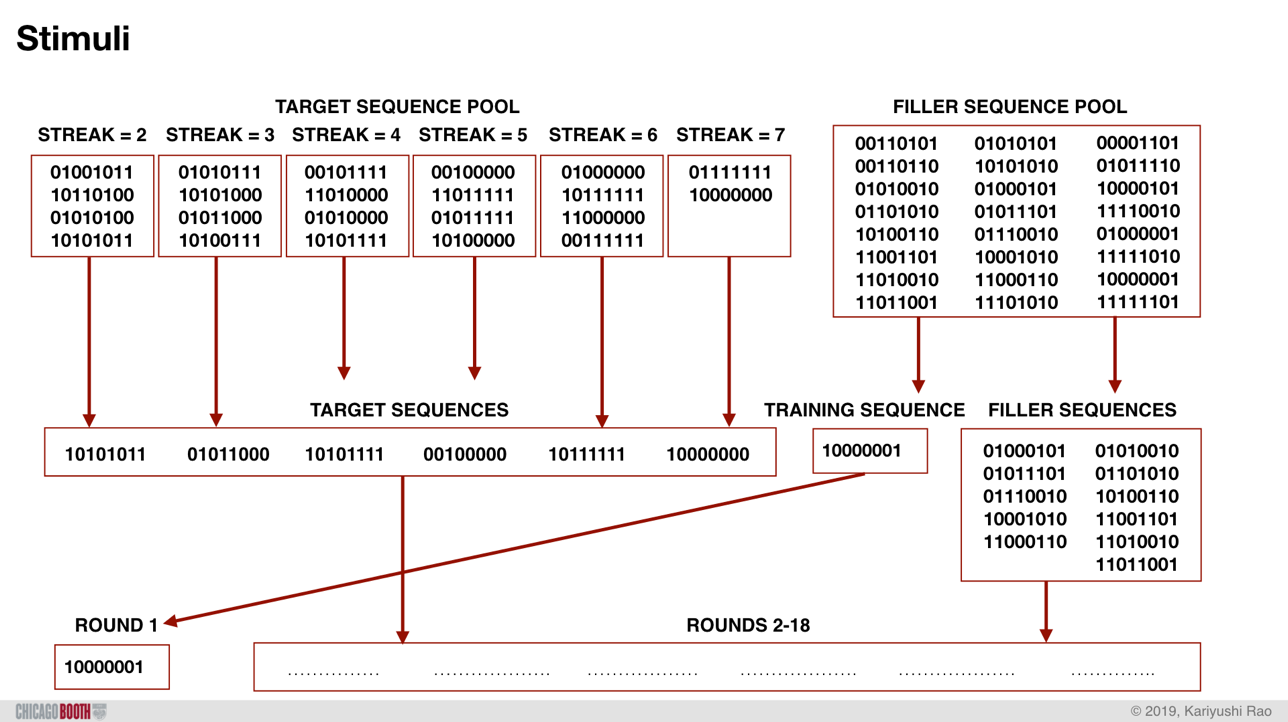


# Chapter 2: Instructions and Procedure

## Section 2.1: Instructions

The transcripts of the instructions provided to participants in each of the present Studies can be found below. Instructions were identical across “A” and “B” versions of each study. Bold, underline, and italic formats that appeared in the original instructions are reproduced in the text below.

***Studies 1A and 1B – Unknown (Ambiguous) Rate***

AnalystUnknown Condition Instructions:

Stock analysts look for trends in the stock market, and use that information to invest their clients’ money wisely. Most stock analysts manage a “book” of stocks, which is a collection of investments they’ve made for their clients. Each quarter, analysts will evaluate trends in the market, and decide whether to sell some of the stocks in their book, purchase more of those stocks, or purchase new (different) stocks. **The result of these decisions determines whether the total value of their book increases or decreases in the subsequent quarter.**

In this task, you will see the **outcomes of investment decisions made by several stock analysts**. We will reveal to you, one at a time, the **change in the value of each analyst’s book over the course of eight quarters** (2 years). If an analyst made good decisions in the prior quarter, then you will see the value of that analyst’s book go up, if the analyst made poor decisions, you will see the value of that analyst’s book go down. **After watching what happens to each analyst’s book** over the course of eight quarters, **your job is to predict what will happen to the analyst’s book in the ninth (next) quarter**: Do you predict it will go up or down? After each prediction, we will move on and show you the results for a new, different stock analyst. This process will repeat 18 times, so you’ll see 18 different stock analysts’ performance histories in total. **Remember that each round** you will see the changes in value of one analyst’s book over 8 quarters, and you will make a prediction about how the value of that analyst’s book will change in the 9th (next) quarter.

StockUnknown Condition Instructions:

Stock prices change constantly. **Price movement of a stock reflects the market’s evaluation, and buyers’ and sellers’ expectations of a company’s worth**. Many factors influence stock prices, such as earnings reports, news about a company’s leadership and products, economic policies, and political events.

In this task, you will observe **changes in different companies’ stock prices.** We will reveal to you, one at a time, the **change in the value of each company’s stock price over the course of eight quarters** (2 years). If the market evaluates a company’s worth as higher than it was worth the previous quarter, then you will see that company’s stock price go up. If the market evaluates a company’s worth as lower than it was worth the previous quarter, you will see that company’s stock price go down. **After watching what happens to each company’s stock price** over the course of eight quarters, **your job is to predict what will happen to the company’s stock price in the ninth (next) quarter**: Do you predict it will go up or down? After each prediction, we will move on and show you the results for a new, different company. This process will repeat 18 times, so you’ll see 18 different companies’ stock price histories in total. **Remember that each round** you will see the changes in one company’s stock price over 8 quarters, and you will make a prediction about how that company’s stock price will change in the 9th (next) quarter.

BingoUnknown Condition Instructions:

In this task you will watch a mechanical bingo machine draw red and blue balls from its **covered** cage. The cage contains **a mix of red and blue balls**. Since the cage is covered, **no one knows exactly how many red balls or how many blue balls are in the cage**. At the start of every round, the machine spins the cage, mixing up all of the balls, and then rolls one ball out of the cage so that an announcer sitting next to the cage can see the color of the ball. The announcer calls out the color of the ball (“red” or “blue”), and the machine rolls the ball back into its covered cage. The machine then spins the cage again, and rolls another ball out. The announcer calls out the color of the ball, and the machine rolls that ball back into its cage. The machine continues **randomly drawing colored balls from the cage until 9 balls have been drawn**. After the 9th ball has been drawn, the round ends, and there is a pause.

Each round, we will reveal to you, one at a time, the colors of the first 8 balls drawn by the machine. **After watching the outcomes** of these eight draws, **your job is to predict the color of the next (9th) ball drawn by the machine**. After you make your prediction about the 9th ball, you will move on to the next round, where you will watch the machine draw another 8 balls and you will again predict the color of the 9th ball. This process will repeat 18 times, so you will watch 18 different rounds of bingo ball draws. **Remember that each round** you will see the color of the balls from the first 8 draws, and you will make a prediction about the color of the 9th ball drawn.

***Studies 2A and 2B – Stationary 50% Rate***

Analyst50 Condition Instructions:

Stock analysts look for trends in the stock market, and use that information to invest their clients' money wisely. Most stock analysts manage a "book" of stocks, which is a collection of investments they've made for their clients. Each quarter, analysts will evaluate trends in the market, and decide whether to sell some of the stocks in their book, purchase more of those stocks, or purchase new (different) stocks. **The result of these decisions determines whether the total value of their book increases or decreases in the subsequent quarter**. If an analyst has a successful quarter and the value of his or her book increases, he or she gets a bonus; but if the book drops in value, there is no bonus and sometimes the analyst has to pay a penalty fine.

In this task, you will see the **outcomes of investment decisions made by several stock analysts**. The analysts you will see all have the same level of skill. **All of the analysts are of Average skill level. The probability that each analyst's book of business will increase in value is always 50%**.

All of the analysts you will see have stable skill levels, and their **skill levels do not change over time**. We will reveal to you, one at a time, the **change in the value of each analyst's book over the course of eight quarters** (2 years). If an analyst made good decisions in the prior quarter, then you will see the value of the book go up, if the analyst made poor decisions, you will see the value of the book go down. **After watching what happens** to each analyst's book over the course of 8 quarters, **your job is to predict what will happen to that analyst's book in the next (9th) quarter**: Do you predict it will go up or down? After each prediction, we will move on and show you the results for a new, different stock analyst. This process will repeat 18 times, so you'll see 18 different stock analysts' performance histories in total. **Remember that each round** you see the changes in value of one analyst’s book over 8 quarters, and you will make a prediction about how the value of that analyst’s book will change in the 9th (next) quarter.

Stock50 Condition Instructions:

Stock prices change constantly. **Price movement of a stock reflects the market’s evaluation, and buyers’ and sellers’ expectations of a company’s worth**. Many factors influence stock prices, such as earnings reports, news about a company’s leadership and products, economic policies, and political events.

In this task, you will observe **changes in different companies’ stock prices**. The companies you will observe all have the same level of performance. **All of the companies are Average performers. The probability that each company's stock price will increase in value is always 50%**.

All of the companies you will see have stable performance levels, and their **performance levels do not change over time**. We will reveal to you, one at a time, the **change in the value of each company’s stock price over the course of eight quarters** (2 years). If the market evaluates a company’s worth as higher than it was worth the previous quarter, then you will see the stock price go up. If the market evaluates a company’s worth as lower than it was worth the previous quarter, you will see the stock price go down. **After watching what happens** to a company's stock price over the course of 8 quarters, **your job is to predict what will happen to that company’s stock price in the next (9th) quarter**: Do you predict it will go up or down? After each prediction, we will move on and show you the results for a new, different company. This process will repeat 18 times, so you'll see 18 different companies’ stock price histories in total. **Remember that each round** you will see the changes in one company’s stock price over 8 quarters, and you will make a prediction about how that company’s stock price will change in the 9th (next) quarter.

Bingo50 Condition Instructions:

In this task you will watch a mechanical bingo machine draw red and blue balls from its **covered** cage. The cage contains **50 red balls and 50 blue balls**. At the start of every round, the machine spins the cage, mixing up all of the balls, and then rolls one ball out of the cage so that an announcer sitting next to the cage can see the color of the ball. The announcer calls out the color of the ball (“red” or “blue”), and the machine rolls the ball back into its covered cage. The machine then spins the cage again, and rolls another ball out. The announcer calls out the color of the ball, and the machine rolls that ball back into its cage. The machine continues **randomly drawing colored balls from the cage until 9 balls have been drawn**. After the 9th ball has been drawn, the round ends, and there is a pause.

Each round, we will reveal to you, one at a time, the colors of the first 8 balls drawn by the machine. **After watching the outcomes** of these eight draws, **your job is to predict the color of the next (9th) ball drawn by the machine**. After you make your prediction about the 9th ball, you will move on to the next round, where you will watch the machine draw another 8 balls and you will again predict the color of the 9th ball. This process will repeat 18 times, so you will watch 18 different rounds of bingo ball draws. **Remember that each round** you will see the color of the balls from the first 8 draws, and you will make a prediction about the color of the 9th ball drawn.

***Studies 3A and 3B ­– Specified Distribution of Rates (.25, .50, .75)***

Analyst25-50-75 Condition Instructions:

Stock analysts look for trends in the stock market, and use that information to invest their clients' money wisely. Most stock analysts manage a "book" of stocks, which is a collection of investments they've made for their clients. Each quarter, analysts will evaluate trends in the market, and decide whether to sell some of the stocks in their book, purchase more of those stocks, or purchase new (different) stocks. **The result of these decisions determines whether the total value of their book increases or decreases in the subsequent quarter**. If an analyst has a successful quarter and the value of his or her book increases, he or she gets a bonus; but if the book drops in value, there is no bonus and sometimes the analyst has to pay a penalty fine.

In this task, you will see the **outcomes of investment decisions made by several stock analysts**. The analysts you will observe have **different skill levels: Bad, Average, and Good**.

- Bad analysts’ book values go up about 25% of the time (1 out of 4 quarters)
- Average analysts’ book values go up about 50% of the time (2 out of 4 quarters)
- Good analysts’ book values go up about 75% of the time (3 out of 4 quarters)

All of the analysts you will see have stable skill levels, and their **skill levels do not change over time**. There are about the same number of Bad, Average, and Good analysts. We will reveal to you, one at a time, the **change in the value of each analyst's book over the course of eight quarters** (2 years). If an analyst made good decisions in the prior quarter, then you will see the value of the book go up, if the analyst made poor decisions, you will see the value of the book go down. **After watching what happens** to each analyst's book over the course of 8 quarters, **your job is to predict what will happen to that analyst's book in the next (9th) quarter**: Do you predict it will go up or down? After each prediction, we will move on and show you the results for a new, different stock analyst. This process will repeat 18 times, so you'll see 18 different stock analysts' performance histories in total. **Remember that each round** you see the changes in value of one analyst’s book over 8 quarters, and you will make a prediction about how the value of that analyst’s book will change in the 9th (next) quarter.

Stock25-50-75 Condition Instructions:

Stock prices change constantly. **Price movement of a stock reflects the market’s evaluation, and buyers’ and sellers’ expectations of a company’s worth**. Many factors influence stock prices, such as earnings reports, news about a company’s leadership and products, economic policies, and political events.

In this task, you will observe **changes in different companies’ stock prices**. The companies you will observe have **different performance levels: Bad, Average, and Good**.

- Bad companies’ stock prices go up about 25% of the time (1 out of 4 quarters)
- Average companies’ stock prices go up about 50% of the time (2 out of 4 quarters)
- Good companies’ stock prices go up about 75% of the time (3 out of 4 quarters)

All of the companies you will see have stable performance levels, and their **performance levels do not change over time**. There are about the same number of Bad, Average, and Good companies. We will reveal to you, one at a time, the **change in the value of each company’s stock price over the course of eight quarters** (2 years). If the market evaluates a company’s worth as higher than it was worth the previous quarter, then you will see the stock price go up. If the market evaluates a company’s worth as lower than it was worth the previous quarter, you will see the stock price go down. **After watching what happens** to a company's stock price over the course of 8 quarters, **your job is to predict what will happen to that company’s stock price in the next (9th) quarter**: Do you predict it will go up or down? After each prediction, we will move on and show you the results for a new, different company. This process will repeat 18 times, so you'll see 18 different companies’ stock price histories in total. **Remember that each round** you will see the changes in one company’s stock price over 8 quarters, and you will make a prediction about how that company’s stock price will change in the 9th (next) quarter.

Bingo25-50-75 Condition Instructions:

In this task you will watch a mechanical bingo machine draw red and blue balls from one of three **covered** cages. Each covered bingo cage contains a mix of 100 red and blue balls.

- Cage #1 contains 25 red balls and 75 blue balls.
- Cage #2 contains 50 red balls and 50 blue balls.
- Cage #3 contains 75 red balls and 25 blue balls.

**At the start of every round**, the machine **randomly selects ONE of the 3 cages**. Each cage has an equal probability of being selected.**Since the cages are covered, no one knows what the mix of red and blue balls is in the cage the machine selects**. The machine spins whichever cage it randomly selected, then rolls one ball out of that cage so that an announcer sitting next to the machine can see the color of the ball. The announcer calls out the color of the ball (“red” or “blue”), and the machine rolls the ball back into the cage. The machine continues **randomly drawing colored balls from the current cage until 9 balls have been drawn**. After the 9th ball has been drawn, there is a pause. **During that pause, the machine selects which covered bingo cage** to use **for the next 9 draws**. The machine is **equally likely to choose any cage**. Sometimes it picks the same cage from the prior round again, sometimes it chooses a new cage. **The machine’s selection is completely random, and totally unrelated to the mix of red and blue balls in the cage it picks**.

Each round, we will reveal to you, one at a time, the colors of the first 8 balls drawn by the machine. **After watching the outcomes** of these eight draws, **your job is to predict the color of the next (9th) ball drawn by the machine from the current cage**. After you make your prediction about the 9th ball, you will move on to the next round, where you will watch the machine draw another 8 balls and you will again predict the color of the 9th ball the machine will draw that round. This process will repeat 18 times, so you will watch 18 different rounds of bingo ball draws. **Remember that each round** you will see the color of the balls from the first 8 draws, and you will make a prediction about the color of the 9th ball drawn.

## Section 2.2: Comprehension Check Questions

In each of the present studies, participants were required to pass a comprehension check after reading the instructions. Participants were allowed to attempt the comprehension check questions as many times as they wished. In Study 1A, participants were not allowed to review the instructions between attempts. We observed a small amount of differential attrition across Conditions at the comprehension check stage of the Study 1A. 12 participants abandoned the procedure after reaching the StockUnknown comprehension check, versus 8 who abandoned after reaching the AnalystUnknown comprehension check, and 2 after reaching the BingoUnknown comprehension check. No other patterns of differential attrition appeared at any other stage of the procedure. In Studies 1B, 2A, 2B, 3A, and 3B the comprehension check page was updated so that the instructions appeared below the comprehension questions. Participants in these studies could refer to the instructions if they were struggling to answer any of the questions. We did not observe differential attrition across conditions in any of these Studies.

The comprehension check questions tested participants understanding of the following:

1. That their task was to predict the *next* outcome in the sequence
2. That each sequence of 8 outcomes was *new* and *not a continuation* of the previous sequences (in the Stock and Analyst Conditions, this meant that each sequence was produced by a *different* company or analyst, respectively).
3. That each sequence of 8 outcomes was produced consecutively by the *same* agent (bingo machine, stock analyst, company).
4. **[Bingo Conditions Only]** That draws from the bingo cage(s) were made *with replacement*.
5. **[Studies 3A and 3B Only]** That the rate at which each agent (bingo machine, stock analyst, company) produced Red/Up outcomes was either 25%, 50%, or 75%, and that there was an equal probability that a given agent produced Red/Up outcomes at each of these rates.

## Section 2.3: Prediction Prompts

Participants were asked to predict the next (9th) outcome of each sequence after watching the sequential revelation of the preceding 8 outcomes. Participants in Studies 1A, 2A, and 3A made their predictions using a continuous sliding scale labeled *0%* on the left-hand side, and *100%* on the right-hand side. Figure OS2 presents a screenshot of the prediction prompt and scale used in the Bingo Conditions of these studies.^^[[1]](#footnote-1)^^ (The inputs for the Analyst and Stock Conditions were identical except for the wording of the question prompts.)

**Figure OS2**

*Screenshot of Prediction Prompt and Response Scale Used in The Bingo Conditions of Studies 1A, 2A, and 3A*


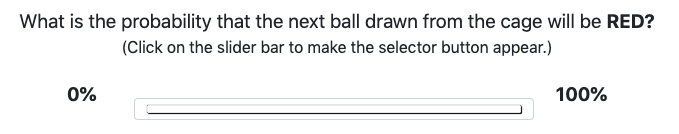


In the Bingo Conditions of Studies 1A, 2A, and 3A, participants were asked, “What is the probability that the next ball drawn from the cage will be **RED**?” In the Analyst Conditions, participants were asked, “What is the probability that next quarter the value of this analyst's book will go **UP**?” In the Stock Conditions, participants were asked, “What is the probability that next quarter the value of this company's stock price will go **UP**?”^[[2]](#footnote-2)^

Participants in Studies 1B, 2B, and 3B made their predictions by selecting one of the two possible outcomes. Figure OS3 presents a screenshot of the prediction prompt and radial response buttons used in the Bingo Conditions of these studies.^[[3]](#footnote-3)^ (The inputs for the Analyst and Stock Conditions were identical except for the wording of the question prompts.)

**Figure OS3**

*Screenshot of Prediction Prompt and Radial Response Used in Bingo Conditions*


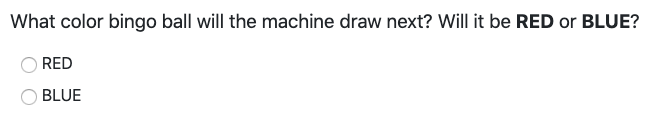


In the Bingo Conditions of Studies 1B, 2B, and 3B, participants were asked, “What color bingo ball will the machine draw next? Will it be **RED** or **BLUE**?” In the Analyst Conditions, participants were asked, “What will happen to the value of this analyst’s book next quarter, will it go **UP** or **DOWN**?” In the Stock Conditions, participants were asked, “What will happen to the value of this company’s stock next quarter? Will it go **UP** or **DOWN**?”

Recall that the dependent variable in the present studies is the participant’s prediction that the last (8th) outcome in each sequence will *repeat*. However, participants were not asked to predict repetition directly; rather, participants in the continuous response (“A”) version of each study were always asked for the probability that the next outcome would be Red (Bingo Conditions) or Up (Analyst and Stock Conditions), and participants in the binary choice (“B”) versions of each study were always asked to choose which outcome they thought would occur next (Red/Up or Blue/Down). Because each Target sequence was randomly drawn from several possible versions ending in either Red (Up) streaks or Blue (Down) streaks, each of the target sequences seen by a participant could end in a streak of Red (Up) signals or a streak of Blue (Down) signals.

For this reason, participants’ responses needed to be recoded to represent their predictions of repetition. For predictions about sequences ending in Red/Up outcomes, we took the raw response (between 0% and 100%) for participants in the continuous (“A”) versions of each Study, and we coded Red/Up as “1” and Blue/Down as “0” for participants in the binary choice (“B”) versions of each Study. For predictions about sequences ending in Blue/Down outcomes, we subtracted the raw response (between 0% and 100%) from 100 for participants in the continuous (“A”) versions of each Study, and we coded Red/Up as “0” and Blue/Down as “1” for participants in the binary choice (“B”) versions of each Study.

We found no significant differences between participants’ predictions for sequences ending in Up or Down outcomes in the Analyst or Stock Conditions of any of our studies. We found no significant differences between participants’ predictions for sequences ending in Red or Blue outcomes in the Bingo Conditions of Studies 1A, 1B, 2B, 3A, or 3B. We found a slight difference between participants’ predictions for sequences ending in Red or Blue outcomes in the Bingo50 Condition of Study 2A. Participants assigned close to 50% probability to repetition of Red streaks across all streak lengths, but slightly lower than 50% probability to repetition of Blue streaks across all streak lengths.

## Section 2.4: Screenshots of Experimental Interface

Figures OS4-OS6 present screenshots of the experimental interface used in each of the present studies. We present a sample from one Condition of each of the continuous (“A”) versions of each Study. The experimental interface for the binary choice (“B”) versions of each Study was identical, except for the question prompts and response inputs (refer to Figure OS3, above).

**Figure OS4**

*Screenshot of Experimental Interface for The AnalystUnknown Condition of Study 1A*


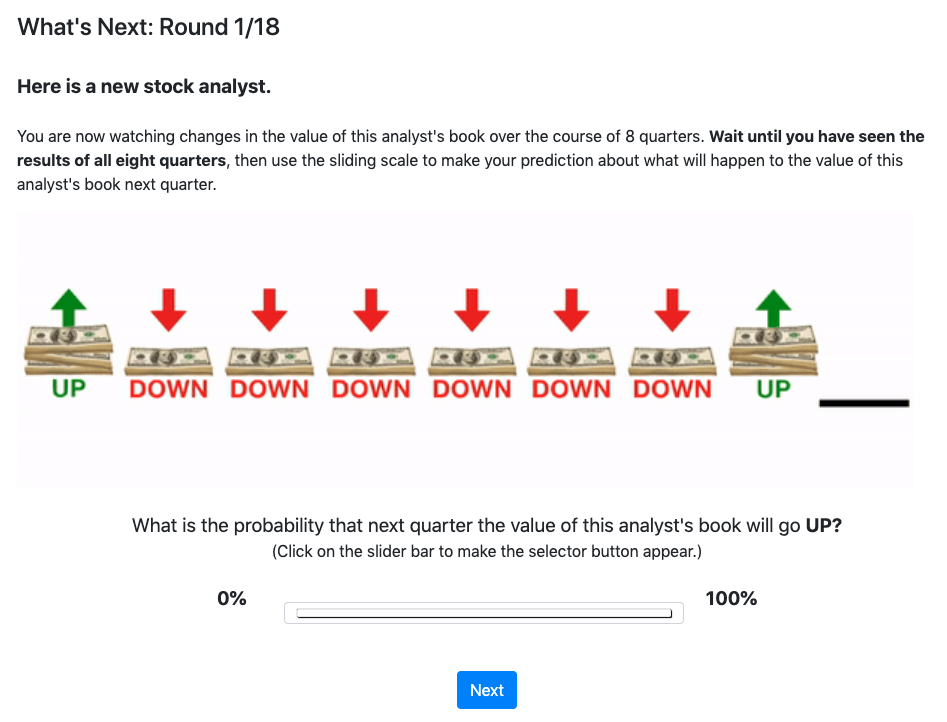


*Note:* Images representing each outcome were revealed one at a time, from left to right, with a 1-second interval between the appearance of each image. In the Analyst Conditions of each of the present studies, Up outcomes were represented by an image of three stacks of dollar bills, with a green arrow pointing up above the stack, and the word “UP” below the stack. Down outcomes were represented by an image of one stack of dollar bills, with a red arrow pointing down above the stack, and the word “DOWN” below the stack. After all 8 stacks were revealed, a black bar beneath the 9th position in the sequence flashed three times. The selector button was hidden so that participants had to click on the sliding scale to make it appear. This step was taken to avoid anchoring participants at any given point on the scale.

**Figure OS5**

*Screenshot of Experimental Interface for The Bingo50 Condition of Study 2A*


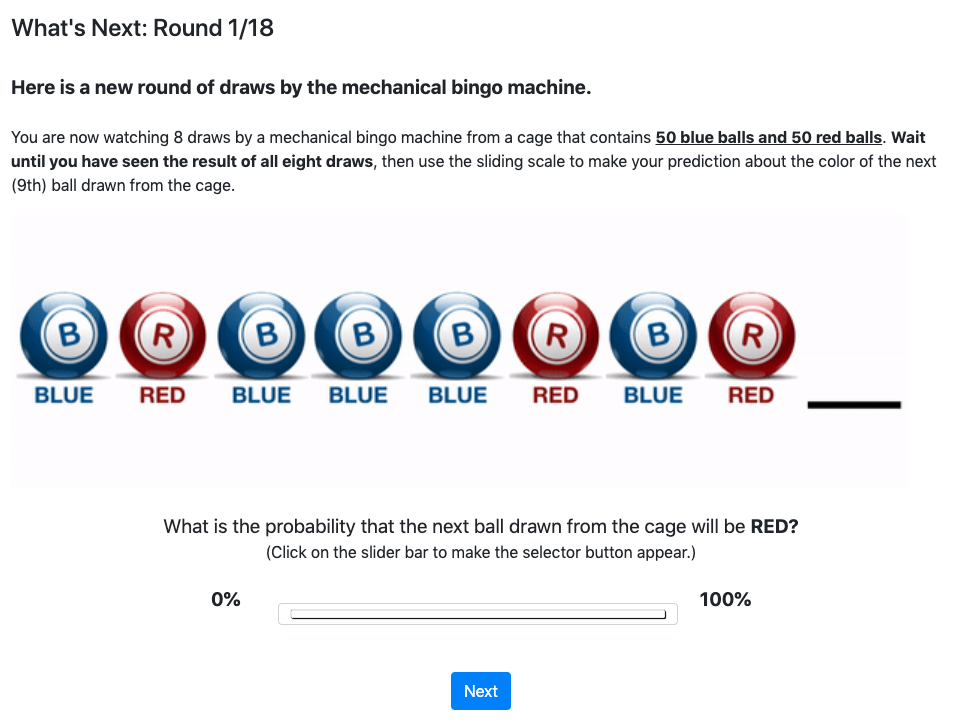


*Note:* Images representing each outcome were revealed one at a time, from left to right, with a 1-second interval between the appearance of each image. In the Bingo Conditions of each of the present studies, Red outcomes were represented by an image of a red bingo ball marked with an “R” in its center, and the word “RED” below the ball. Blue outcomes were represented by an image of a blue bingo ball marked with a “B” in its center, and the word “BLUE” below the ball. After all 8 balls were revealed, a black bar beneath the 9th position in the sequence flashed three times. The selector button was hidden so that participants had to click on the scale to make it appear. This step was taken to avoid anchoring participants at any given point on the scale.

**Figure OS6**

*Screenshot of Experimental Interface for The Stock25-50-75 Condition of Study 3A*


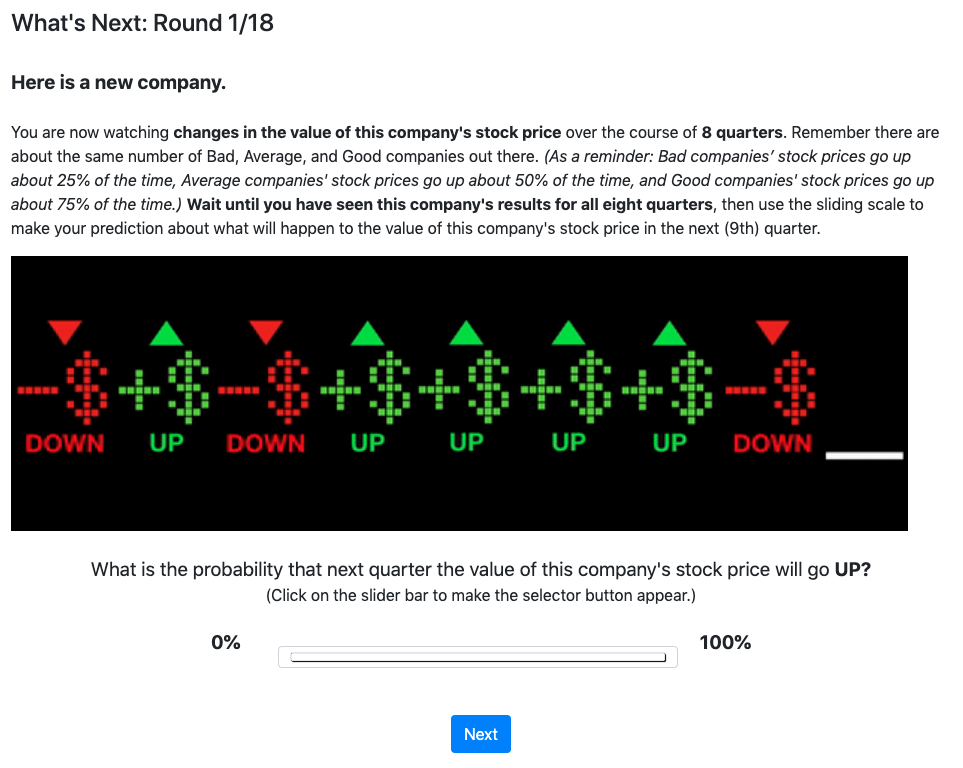


*Note:* Images representing each outcome were revealed one at a time, from left to right, with a 1-second interval between the appearance of each image. In the Stock Conditions of each of the present studies, Up outcomes were represented by an image of a green plus sign next to a green dollar symbol, with a green arrow pointing up above the dollar symbol, and the word “UP” below the dollar symbol. Down outcomes were represented by an image of a minus sign next to a red dollar symbol, with a red arrow pointing down above the dollar symbol, and the word “DOWN” below the dollar symbol. After all 8 symbols were revealed, a white bar beneath the 9th position in the sequence flashed three times. The selector button was hidden so that participants had to click on the scale to make it appear. This step was taken to avoid anchoring participants at any given point on the scale.

#

# Chapter 3: Filler Sequences

Participants in the present studies each judged 12 Filler sequences, in addition to the 6 Target experimental sequences. These 12 Filler sequences were randomly selected from a pool of 24 Filler stimuli that all ended in a reversal (e.g. Blue-Red, Down-Up). Twenty-two of the filler sequences contained a streak of at least 2 identical signals preceding the reversal at the end of the sequence.^[[4]](#footnote-4)^ In the summary that follows, we present participants’ predictions that the next (9th) signal will *repeat* the longest streak of identical signals appearing in each of the 22 Filler sequences.^[[5]](#footnote-5)^

In Studies 1A and 1B, participants’ expectations that the longest streak would repeat increased with Streak Length (Figures OS7 and OS8). The patterns of responses were similar across Conditions in both Studies (Tables OS3 and OS4). In Studies 2A and 2B, participants in the Analyst50 and Stock50 Conditions slightly increased their expectations of repetition as Streak Length increased (Figures OS9 and OS10). Participants in the Bingo50 Condition of Study 2A consistently assigned 50% probability to repetition of the longest streak (Table OS5). In Study 2B, fewer than 50% of participants in Study 2B predicted repetition of the longest streak, across all Streak Lengths (Table OS6). In Studies 3A and 3B, participants’ posterior beliefs are consistent with the Bayesian posteriors for each Filler sequence (Figures OS11 and OS12; Tables OS7 and OS8). Participants in Study 3B were somewhat more likely to predict repetition of the longest streak in the Filler sequences than they were to predict repetition of the terminal streak in the Target sequences (Compare Table OS8 to Table A6 in Appendix A).

**Figure OS7**

*Study 1A: Average Probability Participants Assigned to Repetition of Longest Streak in Each Filler Sequence, by Streak Length and Condition*


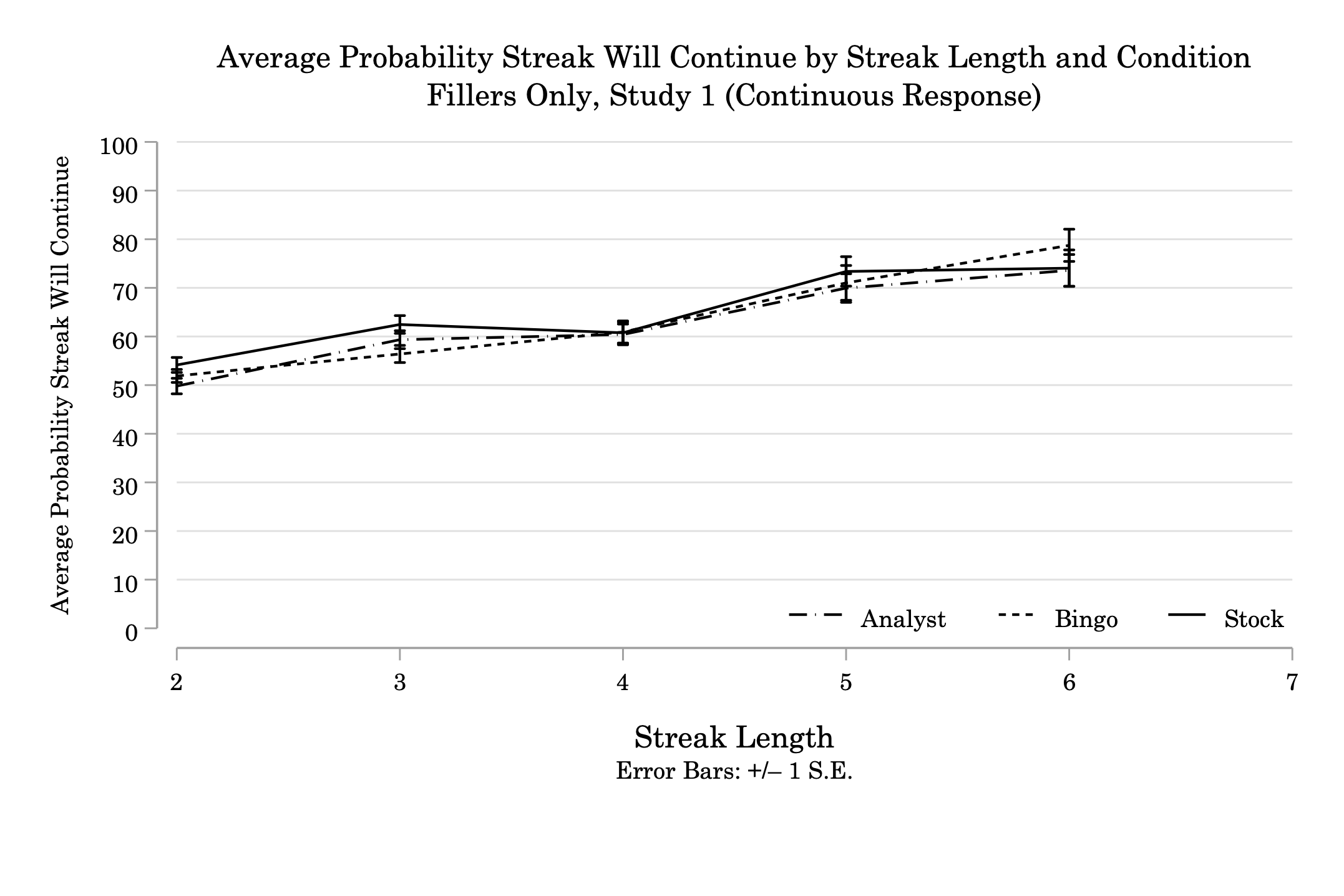


*Note:*  Results of Study 1A. N = 144 (StockUnknown = 44; AnalystUnknown = 50; BingoUnknown = 50). The average probability participants assigned to repetition of the longest streak in each Filler sequence (Error Bars: +/– 1 S.E.). Streak Length is the length of the longest streak preceding the reversal at the end of each sequence. Participants increased the probability they assigned to repetition of the longest streak in the Filler sequences as the length of that streak increased. Filler sequences contained streaks that ranged from 2 to 6 identical signals.

**Table OS3**

*Study 1A: Average Probability Participants Assigned to Repetition of Longest Streak in Each Filler Sequence*

|  | **Streak Type** | **Analyst** | |  | **Bingo** | |  | **Stock** | |
| --- | --- | --- | --- | --- | --- | --- | --- | --- | --- |
|  |  | **N** | **Mean Repeat** |  | **N** | **Mean Repeat** |  | **N** | **Mean Repeat** |
| **Streak Length = 2** |  | **178** | **49.80** |  | **207** | **51.89** |  | **182** | **54.15** |
| 00**11**0101 | 1 | 23 | 48.61 |  | 19 | 51.84 |  | 23 | 55.04 |
| 00110**11**0 | 1 | 20 | 53.20 |  | 27 | 49.59 |  | 19 | 61.42 |
| 0101**00**10 | 0 | 19 | 55.21 |  | 25 | 57.32 |  | 21 | 51.29 |
| 0**11**01010 | 1 | 16 | 60.88 |  | 21 | 68.48 |  | 28 | 59.54 |
| 10100**11**0 | 1 | 23 | 48.30 |  | 29 | 57.66 |  | 26 | 52.96 |
| 1100**11**01 | 1 | 26 | 57.73 |  | 31 | 46.13 |  | 21 | 66.67 |
| 1101**00**10 | 0 | 24 | 46.50 |  | 28 | 48.18 |  | 21 | 49.81 |
| 11011**00**1 | 0 | 27 | 34.52 |  | 27 | 40.56 |  | 23 | 37.17 |
| **Streak Length = 3** |  | **158** | **59.34** |  | **138** | **56.41** |  | **132** | **62.47** |
| 01**000**101 | 0 | 23 | 66.70 |  | 20 | 62.40 |  | 29 | 65.62 |
| 010**111**01 | 1 | 27 | 58.11 |  | 21 | 56.76 |  | 21 | 64.86 |
| 0**111**0010 | 1 | 23 | 56.74 |  | 21 | 56.95 |  | 22 | 48.77 |
| 1**000**1010 | 0 | 32 | 55.25 |  | 25 | 57.48 |  | 22 | 56.50 |
| 11**000**110 | 0 | 25 | 54.76 |  | 26 | 48.15 |  | 18 | 66.67 |
| **111**01010 | 1 | 28 | 65.39 |  | 25 | 58.36 |  | 20 | 73.25 |
| **Streak Length = 4** |  | **110** | **60.42** |  | **104** | **60.92** |  | **83** | **60.76** |
| **0000**1101 | 0 | 25 | 58.72 |  | 27 | 58.67 |  | 21 | 53.33 |
| 010**1111**0 | 1 | 27 | 62.44 |  | 25 | 59.52 |  | 21 | 65.71 |
| 1**0000**101 | 0 | 28 | 64.64 |  | 27 | 66.19 |  | 20 | 66.70 |
| **1111**0010 | 1 | 30 | 56.07 |  | 25 | 59.08 |  | 21 | 57.57 |
| **Streak Length = 5** |  | **46** | **69.96** |  | **52** | **71.02** |  | **42** | **73.38** |
| 01**00000**1 | 0 | 25 | 67.08 |  | 27 | 73.85 |  | 19 | 81.79 |
| **11111**010 | 1 | 21 | 73.38 |  | 25 | 67.96 |  | 23 | 66.43 |
| **Streak Length = 6** |  | **54** | **73.59** |  | **47** | **78.74** |  | **42** | **74.05** |
| 1**000000**1 | 0 | 28 | 69.29 |  | 22 | 73.55 |  | 17 | 59.00 |
| **111111**01 | 1 | 26 | 78.23 |  | 25 | 83.32 |  | 25 | 84.28 |

*Note:* Several of the sequences with a maximum streak length of 2 contained more than one streak of length 2. In these cases, we considered the streak occurring closest to the end of the sequence as the focal streak in the analysis. Focal streaks are highlighted in bold type in the first column of the table. (Coding: 1 = UP/RED; 0 = DOWN/BLUE.)

**Figure OS8**

*Study 1B: Proportion of Participants Predicting Repetition of Longest Streak in Each Filler Sequence, by Streak Length and Condition*


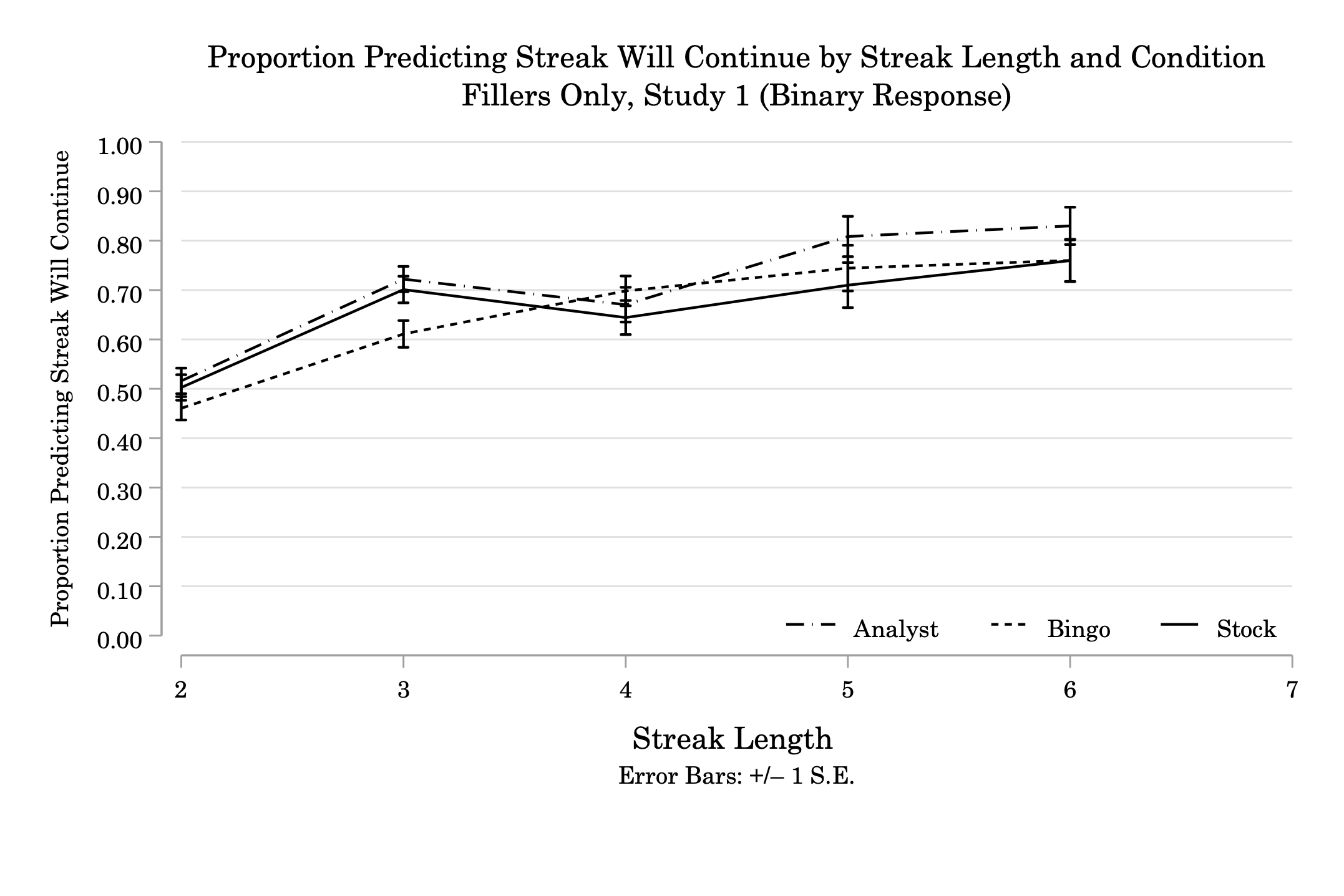


*Note:* Results of Study 1B. N = 300 (StockUnknown = 97; AnalystUnknown = 95; BingoUnknown = 108). Proportion of participants predicting repetition of the longest streak in each filler sequence (Error Bars: +/– 1 S.E.). Streak Length is the length of the longest streak present in the Filler sequence, prior to the reversal at the end of that sequence. The proportion of participants predicting repetition increased as the length of that streak increased. Filler sequences contained streaks that ranged from 2 to 6 identical signals in length.

**Table OS4**

*Study 1B: Proportion of Participants Predicting Repetition of Longest Streak in Each Filler Sequence, by Streak Length and Condition*

|  | **Streak Type** | **Analyst** | |  | **Bingo** | |  | **Stock** | |
| --- | --- | --- | --- | --- | --- | --- | --- | --- | --- |
|  |  | **N** | **Prop Repeat** |  | **N** | **Prop Repeat** |  | **N** | **Prop Repeat** |
| **Streak Length = 2** |  | **374** | **0.52** |  | **443** | **0.46** |  | **374** | **0.50** |
| 00**11**0101 | 1 | 50 | 0.52 |  | 48 | 0.40 |  | 56 | 0.38 |
| 00110**11**0 | 1 | 50 | 0.66 |  | 57 | 0.58 |  | 39 | 0.54 |
| 0101**00**10 | 0 | 47 | 0.51 |  | 64 | 0.48 |  | 53 | 0.49 |
| 0**11**01010 | 1 | 56 | 0.82 |  | 57 | 0.68 |  | 44 | 0.82 |
| 10100**11**0 | 1 | 40 | 0.55 |  | 54 | 0.44 |  | 46 | 0.48 |
| 1100**11**01 | 1 | 38 | 0.47 |  | 54 | 0.50 |  | 46 | 0.65 |
| 1101**00**10 | 0 | 50 | 0.40 |  | 57 | 0.37 |  | 49 | 0.41 |
| 11011**00**1 | 0 | 43 | 0.09 |  | 52 | 0.19 |  | 41 | 0.29 |
| **Streak Length = 3** |  | **306** | **0.72** |  | **324** | **0.61** |  | **291** | **0.70** |
| 01**000**101 | 0 | 52 | 0.73 |  | 51 | 0.76 |  | 47 | 0.77 |
| 010**111**01 | 1 | 56 | 0.79 |  | 56 | 0.63 |  | 39 | 0.69 |
| 0**111**0010 | 1 | 43 | 0.53 |  | 51 | 0.53 |  | 45 | 0.51 |
| 1**000**1010 | 0 | 44 | 0.64 |  | 59 | 0.54 |  | 54 | 0.63 |
| 11**000**110 | 0 | 54 | 0.70 |  | 55 | 0.58 |  | 53 | 0.74 |
| **111**01010 | 1 | 57 | 0.88 |  | 52 | 0.63 |  | 53 | 0.85 |
| **Streak Length = 4** |  | **179** | **0.67** |  | **232** | **0.70** |  | **194** | **0.64** |
| **0000**1101 | 0 | 49 | 0.49 |  | 53 | 0.68 |  | 56 | 0.50 |
| 010**1111**0 | 1 | 46 | 0.78 |  | 58 | 0.72 |  | 44 | 0.77 |
| 1**0000**101 | 0 | 39 | 0.79 |  | 65 | 0.77 |  | 46 | 0.74 |
| **1111**0010 | 1 | 45 | 0.64 |  | 56 | 0.61 |  | 48 | 0.60 |
| **Streak Length = 5** |  | **94** | **0.81** |  | **90** | **0.74** |  | **100** | **0.71** |
| 01**00000**1 | 0 | 44 | 0.75 |  | 41 | 0.76 |  | 53 | 0.60 |
| **11111**010 | 1 | 50 | 0.86 |  | 49 | 0.73 |  | 47 | 0.83 |
| **Streak Length = 6** |  | **100** | **0.83** |  | **100** | **0.76** |  | **104** | **0.76** |
| 1**000000**1 | 0 | 52 | 0.73 |  | 50 | 0.74 |  | 54 | 0.61 |
| **111111**01 | 1 | 48 | 0.94 |  | 50 | 0.78 |  | 50 | 0.92 |

*Note:* Focal streaks are highlighted in bold type in the first column of the table.

**Figure OS9**

*Study 2A: Average Probability Participants Assigned to Repetition of Longest Streak in Each Filler Sequence, by Streak Length and Condition*


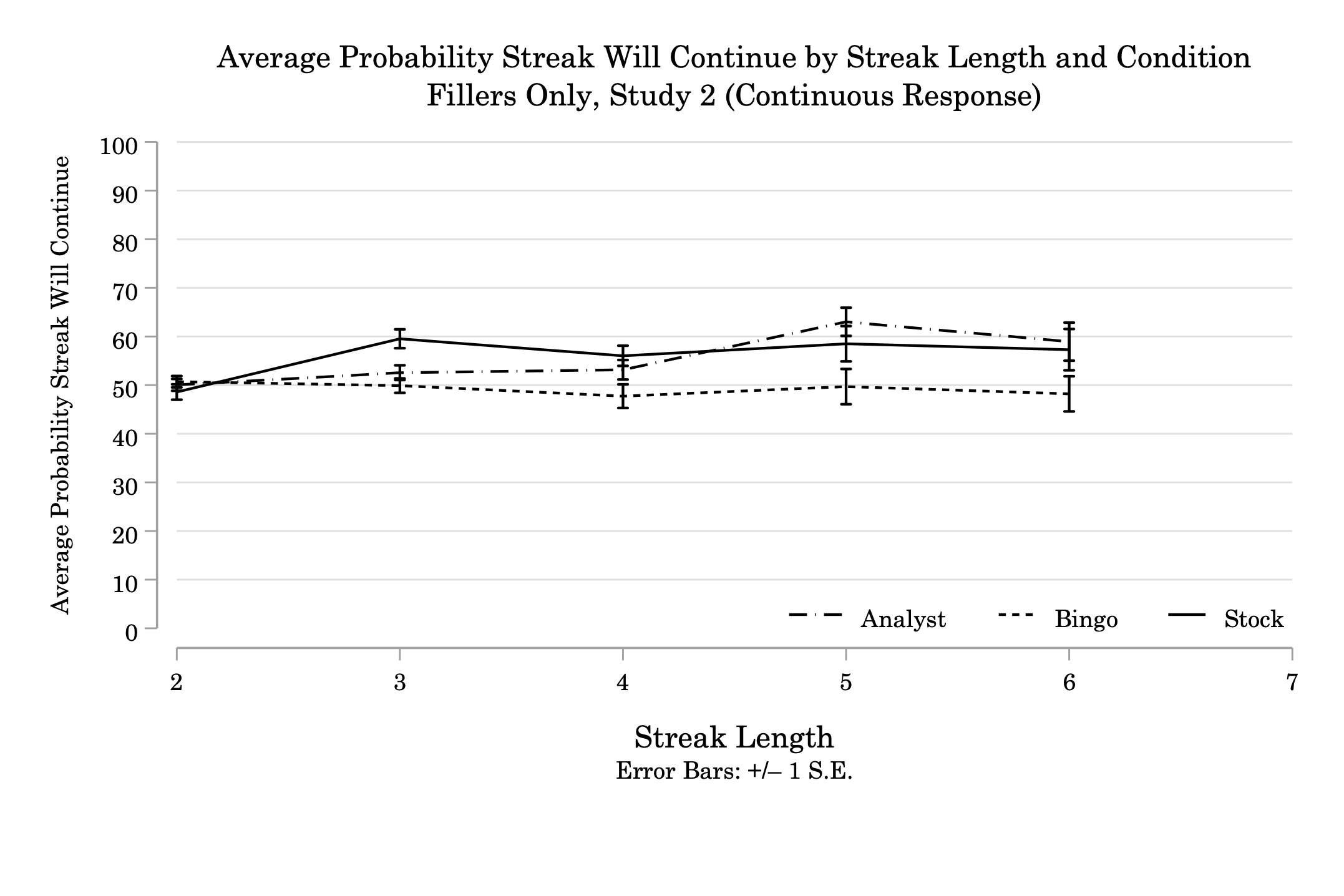


*Note:* Results of Study 2A. N = 156 (Stock50 = 48; Analyst50 = 52; Bingo50 = 56). The average probability participants assigned to repetition of the longest streak in each Filler sequence (Error Bars: +/– 1 S.E.). Streak Length is the length of the longest streak preceding the reversal at the end of each sequence. Participants in the Analyst50 Condition gradually increased the probability they assigned to repetition of the longest streak in the Filler sequences as the length of that streak increased. Participants in the Stock50 Condition initially increase the probability they assign to repetition when Streak Length increases from 2 to 3, but have a flat slope across Streak Lengths 3 to 6. Participants in the Bingo50 Condition have a flat slope across streak lengths - they stick pretty close to 50% probability of repetition across streak lengths.

**Table OS5**

*Study 2A: Average Probability Participants Assigned to Repetition of Longest Streak in Each Filler Sequence*

|  | **Streak Type** | **Analyst** | |  | **Bingo** | |  | **Stock** | |
| --- | --- | --- | --- | --- | --- | --- | --- | --- | --- |
|  |  | **N** | **Mean Repeat** |  | **N** | **Mean Repeat** |  | **N** | **Mean Repeat** |
| **Streak Length = 2** |  | **218** | **50.06** |  | **236** | **50.70** |  | **203** | **48.58** |
| 00**11**0101 | 1 | 30 | 51.60 |  | 30 | 51.60 |  | 27 | 41.89 |
| 00110**11**0 | 1 | 29 | 51.52 |  | 35 | 53.63 |  | 27 | 50.30 |
| 0101**00**10 | 0 | 18 | 44.22 |  | 24 | 42.46 |  | 32 | 51.34 |
| 0**11**01010 | 1 | 27 | 55.59 |  | 38 | 56.26 |  | 20 | 56.90 |
| 10100**11**0 | 1 | 28 | 51.36 |  | 29 | 55.14 |  | 33 | 50.12 |
| 1100**11**01 | 1 | 28 | 53.00 |  | 23 | 55.96 |  | 19 | 56.11 |
| 1101**00**10 | 0 | 30 | 44.87 |  | 28 | 41.89 |  | 20 | 47.50 |
| 11011**00**1 | 0 | 28 | 46.61 |  | 29 | 45.69 |  | 25 | 36.88 |
| **Streak Length = 3** |  | **143** | **52.56** |  | **160** | **49.89** |  | **128** | **59.53** |
| 01**000**101 | 0 | 17 | 60.00 |  | 28 | 43.79 |  | 26 | 59.19 |
| 010**111**01 | 1 | 32 | 53.63 |  | 20 | 56.00 |  | 20 | 56.75 |
| 0**111**0010 | 1 | 20 | 48.70 |  | 30 | 52.70 |  | 23 | 51.52 |
| 1**000**1010 | 0 | 24 | 51.75 |  | 29 | 44.45 |  | 19 | 56.42 |
| 11**000**110 | 0 | 22 | 46.73 |  | 21 | 51.33 |  | 23 | 67.78 |
| **111**01010 | 1 | 28 | 54.86 |  | 32 | 52.78 |  | 17 | 66.47 |
| **Streak Length = 4** |  | **103** | **53.16** |  | **107** | **47.73** |  | **102** | **56.03** |
| **0000**1101 | 0 | 25 | 53.68 |  | 32 | 40.53 |  | 23 | 50.83 |
| 010**1111**0 | 1 | 27 | 53.67 |  | 28 | 49.71 |  | 34 | 58.09 |
| 1**0000**101 | 0 | 27 | 58.07 |  | 20 | 48.85 |  | 23 | 63.26 |
| **1111**0010 | 1 | 24 | 46.50 |  | 27 | 53.37 |  | 22 | 50.73 |
| **Streak Length = 5** |  | **53** | **63.02** |  | **55** | **49.69** |  | **45** | **58.51** |
| 01**00000**1 | 0 | 27 | 64.11 |  | 30 | 44.83 |  | 20 | 57.20 |
| **11111**010 | 1 | 26 | 61.88 |  | 25 | 55.52 |  | 25 | 59.56 |
| **Streak Length = 6** |  | **51** | **58.94** |  | **63** | **48.21** |  | **52** | **57.29** |
| 1**000000**1 | 0 | 27 | 58.48 |  | 30 | 43.73 |  | 29 | 52.79 |
| **111111**01 | 1 | 24 | 59.46 |  | 33 | 52.27 |  | 23 | 62.96 |

*Note:* Focal streaks are highlighted in bold type in the first column of the table.

**Figure OS10**

*Study 2B: Proportion of Participants Predicting Repetition of Longest Streak in Each Filler Sequence, by Streak Length and Condition*


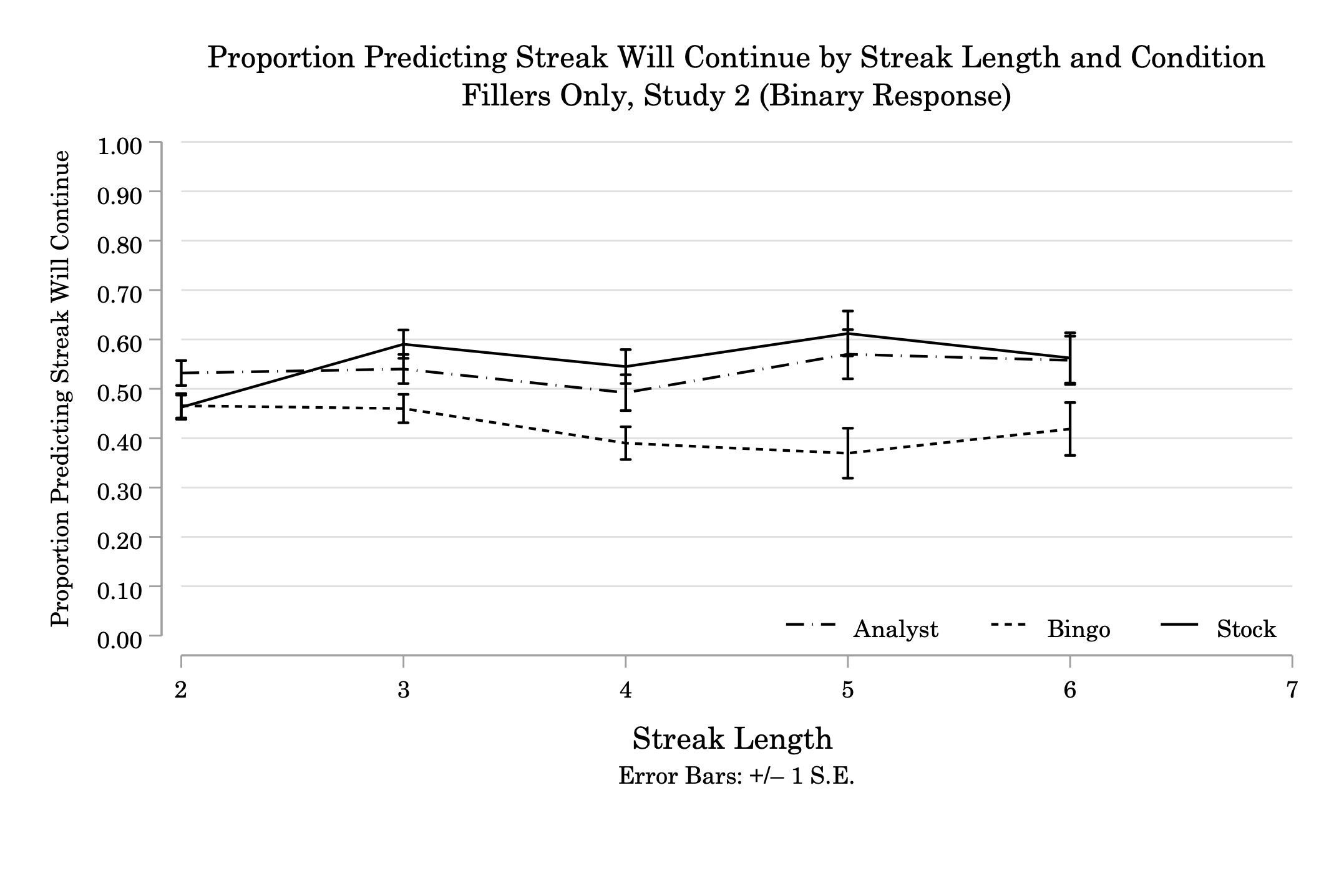


*Note:* Results of Study 2B. N = 301 (Stock50 = 103; Analyst50 = 97; Bingo50 = 101). Proportion of participants predicting repetition of the longest streak in each filler sequence (Error Bars: +/– 1 S.E.). Streak Length is the length of the longest streak present in the Filler sequence, prior to the reversal at the end of that sequence. Filler sequences contained streaks that ranged from 2 to 6 identical signals in length. The updating pattern for participants submitting binary responses in the Analyst50 and Stock50 Conditions of Study 2B is similar to the pattern we observed among participants submitting continuous responses in Study 2A. But, participants submitting binary responses in the Bingo50 Condition of Study 2B show a stronger preference for reversal than those submitting continuous responses in Study 2A. The proportion of participants predicting repetition increased slightly between Streak Lengths 2 and 6 in the Analyst50 and Stock50 Conditions. In the Bingo50 Condition, the proportion of participants predicting repetition of the Longest Streak decreased slightly with Streak Length.

**Table OS6**

*Study 2B: Proportion of Participants Predicting Repetition of Longest Streak in Each Filler Sequence, by Streak Length and Condition*

|  | **Streak Type** | **Analyst** | |  | **Bingo** | |  | **Stock** | |
| --- | --- | --- | --- | --- | --- | --- | --- | --- | --- |
|  |  | **N** | **Prop Repeat** |  | **N** | **Prop Repeat** |  | **N** | **Prop Repeat** |
| **Streak Length = 2** |  | **391** | **0.53** |  | **408** | **0.47** |  | **415** | **0.46** |
| 00**11**0101 | 1 | 50 | 0.46 |  | 60 | 0.37 |  | 49 | 0.43 |
| 00110**11**0 | 1 | 57 | 0.70 |  | 54 | 0.44 |  | 61 | 0.54 |
| 0101**00**10 | 0 | 46 | 0.35 |  | 40 | 0.38 |  | 49 | 0.33 |
| 0**11**01010 | 1 | 48 | 0.83 |  | 49 | 0.63 |  | 45 | 0.76 |
| 10100**11**0 | 1 | 38 | 0.74 |  | 53 | 0.60 |  | 50 | 0.64 |
| 1100**11**01 | 1 | 47 | 0.43 |  | 55 | 0.31 |  | 51 | 0.57 |
| 1101**00**10 | 0 | 56 | 0.32 |  | 49 | 0.39 |  | 48 | 0.21 |
| 11011**00**1 | 0 | 49 | 0.47 |  | 48 | 0.63 |  | 62 | 0.27 |
| **Streak Length = 3** |  | **287** | **0.54** |  | **300** | **0.46** |  | **293** | **0.59** |
| 01**000**101 | 0 | 45 | 0.60 |  | 54 | 0.43 |  | 42 | 0.45 |
| 010**111**01 | 1 | 48 | 0.48 |  | 48 | 0.23 |  | 51 | 0.57 |
| 0**111**0010 | 1 | 54 | 0.56 |  | 51 | 0.59 |  | 47 | 0.68 |
| 1**000**1010 | 0 | 46 | 0.37 |  | 47 | 0.26 |  | 50 | 0.38 |
| 11**000**110 | 0 | 52 | 0.48 |  | 51 | 0.55 |  | 51 | 0.65 |
| **111**01010 | 1 | 42 | 0.79 |  | 49 | 0.69 |  | 52 | 0.79 |
| **Streak Length = 4** |  | **191** | **0.49** |  | **218** | **0.39** |  | **211** | **0.55** |
| **0000**1101 | 0 | 51 | 0.29 |  | 55 | 0.29 |  | 58 | 0.41 |
| 010**1111**0 | 1 | 45 | 0.60 |  | 51 | 0.45 |  | 55 | 0.65 |
| 1**0000**101 | 0 | 44 | 0.55 |  | 55 | 0.47 |  | 53 | 0.60 |
| **1111**0010 | 1 | 51 | 0.55 |  | 57 | 0.35 |  | 45 | 0.51 |
| **Streak Length = 5** |  | **100** | **0.57** |  | **92** | **0.37** |  | **116** | **0.61** |
| 01**00000**1 | 0 | 46 | 0.50 |  | 44 | 0.36 |  | 63 | 0.56 |
| **11111**010 | 1 | 54 | 0.63 |  | 48 | 0.38 |  | 53 | 0.68 |
| **Streak Length = 6** |  | **104** | **0.56** |  | **86** | **0.42** |  | **96** | **0.56** |
| 1**000000**1 | 0 | 54 | 0.43 |  | 40 | 0.45 |  | 60 | 0.47 |
| **111111**01 | 1 | 50 | 0.70 |  | 46 | 0.39 |  | 36 | 0.72 |

*Note:* Focal streaks are highlighted in bold type in the first column of the table.

**Figure OS11**

*Study 3A: Average Probability Participants Assigned to Repetition of Longest Streak in Each Filler Sequence, by Streak Length and Condition*


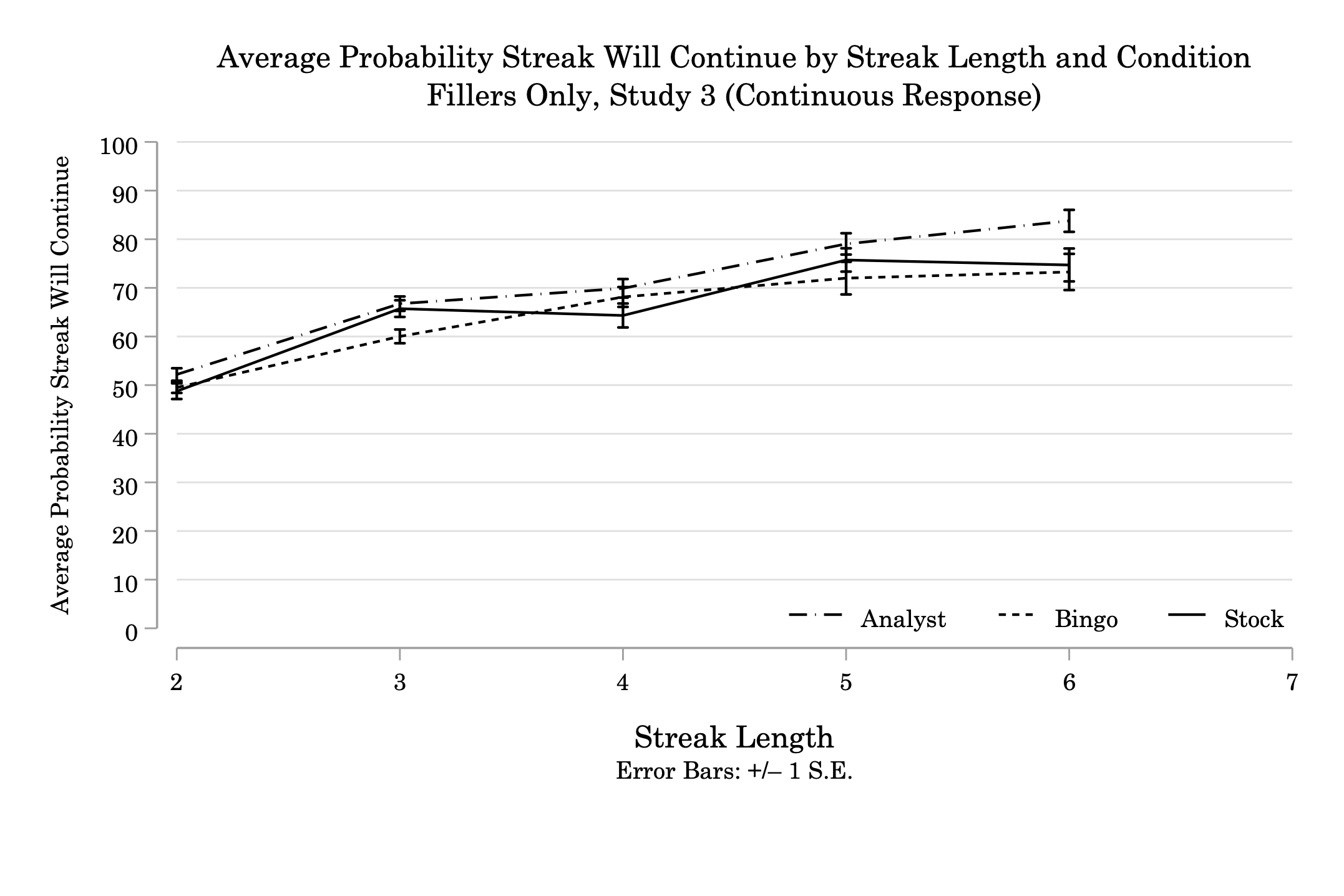


*Note:* N = 150 (Stock25-50-75 = 50; Analyst25-50-75 = 50; Bingo25-50-75 = 50). The average probability participants assigned to repetition of the longest streak in each Filler sequence (Error Bars: +/– 1 S.E.). Streak Length is the length of the longest streak preceding the reversal at the end of each sequence. Filler sequences contained streaks that ranged from 2 to 6 identical signals. Participants in all 3 Conditions increased the probability they assigned to repetition of the longest streak as the length of that streak increased. Participants in the Analyst25-50-75 Condition assigned slightly higher probability to streaks of length 6 than did participants in the other two Conditions, but otherwise predictions were similar across all 3 Conditions.

**Table OS7**

*Study 3A: Average Probability Participants Assigned to Repetition of Longest Streak in Each Filler Sequence*

*Note:* Focal streaks are highlighted in bold type in the first column of the table.

|  | **Streak Type** | **Analyst** | |  | **Bingo** | |  | **Stock** | |
| --- | --- | --- | --- | --- | --- | --- | --- | --- | --- |
|  |  | **N** | **Mean Repeat** |  | **N** | **Mean Repeat** |  | **N** | **Mean Repeat** |
| **Streak Length = 2** |  | **203** | **52.19** |  | **189** | **49.51** |  | **186** | **48.75** |
| 00**11**0101 | 1 | 31 | 53.23 |  | 25 | 48.48 |  | 23 | 41.61 |
| 00110**11**0 | 1 | 20 | 49.55 |  | 26 | 50.85 |  | 18 | 48.83 |
| 0101**00**10 | 0 | 22 | 54.73 |  | 23 | 52.17 |  | 23 | 50.78 |
| 0**11**01010 | 1 | 24 | 57.71 |  | 28 | 55.61 |  | 26 | 56.00 |
| 10100**11**0 | 1 | 25 | 49.24 |  | 20 | 53.15 |  | 26 | 59.42 |
| 1100**11**01 | 1 | 29 | 66.66 |  | 19 | 58.11 |  | 23 | 61.70 |
| 1101**00**10 | 0 | 27 | 54.19 |  | 27 | 46.41 |  | 22 | 40.45 |
| 11011**00**1 | 0 | 25 | 29.48 |  | 21 | 30.76 |  | 25 | 30.12 |
| **Streak Length = 3** |  | **148** | **66.74** |  | **156** | **60.02** |  | **141** | **65.73** |
| 01**000**101 | 0 | 30 | 72.63 |  | 30 | 66.27 |  | 27 | 74.67 |
| 010**111**01 | 1 | 25 | 71.12 |  | 26 | 67.62 |  | 19 | 67.53 |
| 0**111**0010 | 1 | 17 | 56.47 |  | 21 | 57.76 |  | 22 | 59.59 |
| 1**000**1010 | 0 | 26 | 70.50 |  | 29 | 67.59 |  | 21 | 66.48 |
| 11**000**110 | 0 | 22 | 54.18 |  | 27 | 48.04 |  | 25 | 52.52 |
| **111**01010 | 1 | 28 | 69.14 |  | 23 | 49.87 |  | 27 | 72.19 |
| **Streak Length = 4** |  | **98** | **69.90** |  | **97** | **68.12** |  | **103** | **64.32** |
| **0000**1101 | 0 | 19 | 63.21 |  | 23 | 66.48 |  | 28 | 56.54 |
| 010**1111**0 | 1 | 28 | 72.50 |  | 29 | 66.62 |  | 28 | 68.93 |
| 1**0000**101 | 0 | 28 | 74.57 |  | 22 | 69.86 |  | 23 | 66.13 |
| **1111**0010 | 1 | 23 | 66.57 |  | 23 | 70.00 |  | 24 | 66.29 |
| **Streak Length = 5** |  | **52** | **79.04** |  | **58** | **72.00** |  | **60** | **75.73** |
| 010**0000**1 | 0 | 26 | 77.23 |  | 34 | 71.06 |  | 26 | 74.65 |
| **11111**010 | 1 | 26 | 80.85 |  | 24 | 73.33 |  | 34 | 76.56 |
| **Streak Length = 6** |  | **58** | **83.78** |  | **49** | **73.27** |  | **55** | **74.71** |
| 1**000000**1 | 0 | 29 | 83.66 |  | 24 | 70.71 |  | 22 | 68.77 |
| **111111**01 | 1 | 29 | 83.90 |  | 25 | 75.72 |  | 33 | 78.67 |

**Figure OS12**

*Study 3B: Proportion of Participants Predicting Repetition of the Longest Streak in Each Filler Sequence, by Streak Length and Condition*


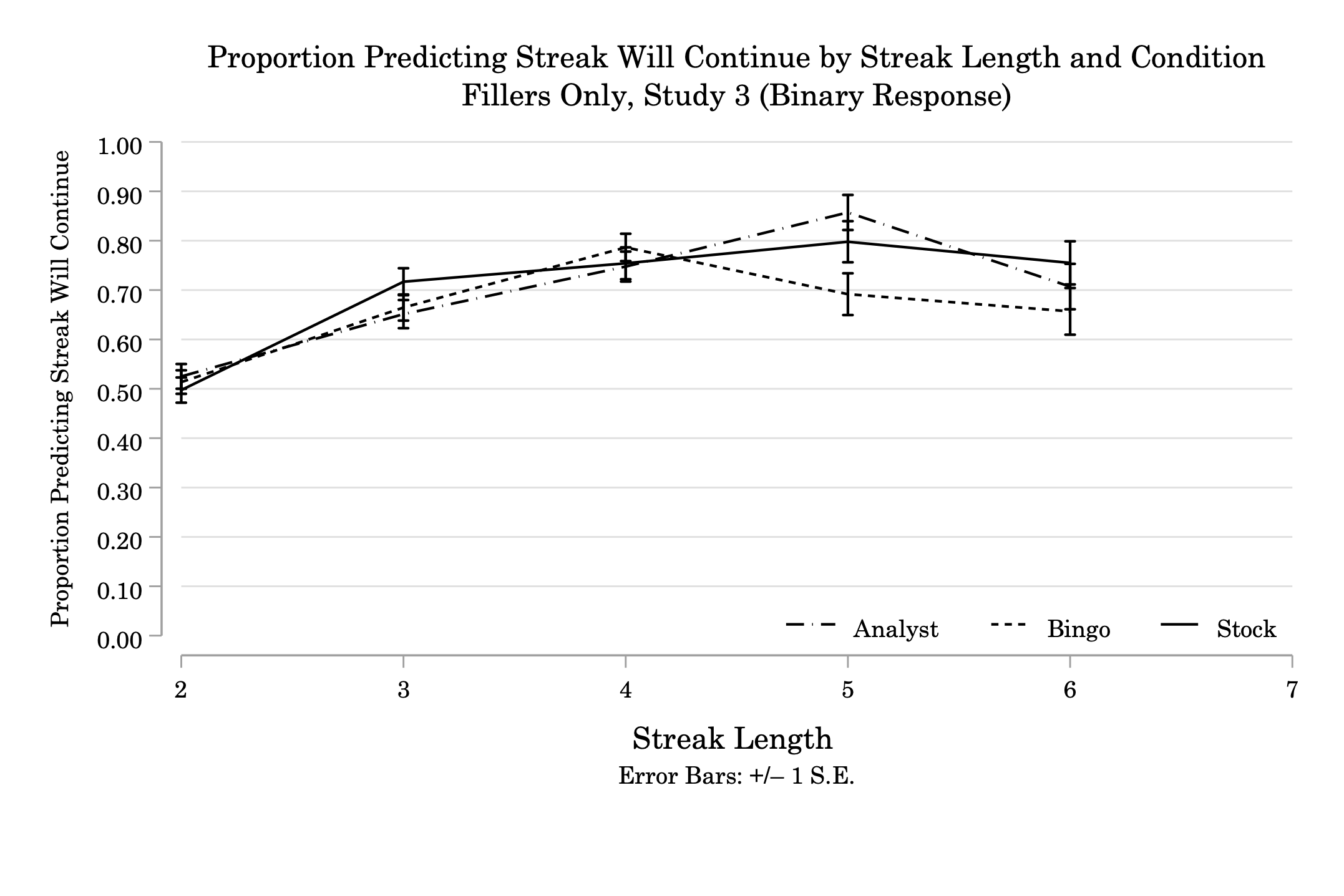


*Note:* N = 300 (Stock25-50-75 = 93; Analyst25-50-75 = 98; Bingo25-50-75 = 109). Proportion of participants predicting repetition of the longest streak in each filler sequence (Error Bars: +/– 1 S.E.). Streak Length is the length of the longest streak present in the Filler sequence, prior to the reversal at the end of that sequence. Filler sequences contained streaks that ranged from 2 to 6 identical signals in length. In all 3 Conditions, the proportion of participants predicting repetition increased across Streak Lengths 2 to 4. In the Analyst25-50-75 and Stock25-50-75 Conditions, the proportion of participants predicting repetition continued to increase between Streak Lengths 4 and 5, before tapering off at Streak Length 6. In the Bingo 25-50-75Condition, the proportion of participants predicting repetition declines across Streak Lengths 4 to 6.

**Table OS8**

*Study 3B: Proportion of Participants Predicting Repetition of Longest Streak in Each Filler Sequence, by Streak Length and Condition*

|  | **Streak Type** | **Analyst** | |  | **Bingo** | |  | **Stock** | |
| --- | --- | --- | --- | --- | --- | --- | --- | --- | --- |
|  |  | **N** | **Prop Repeat** |  | **N** | **Prop Repeat** |  | **N** | **Prop Repeat** |
| **Streak Length = 2** |  | **398** | **0.53** |  | **440** | **0.51** |  | **384** | **0.50** |
| 00**11**0101 | 1 | 52 | 0.50 |  | 60 | 0.48 |  | 43 | 0.47 |
| 00110**11**0 | 1 | 46 | 0.65 |  | 54 | 0.46 |  | 52 | 0.58 |
| 0101**00**10 | 0 | 55 | 0.44 |  | 52 | 0.50 |  | 54 | 0.35 |
| 0**11**01010 | 1 | 56 | 0.73 |  | 50 | 0.58 |  | 44 | 0.77 |
| 10100**11**0 | 1 | 46 | 0.50 |  | 59 | 0.59 |  | 41 | 0.73 |
| 1100**11**01 | 1 | 47 | 0.68 |  | 61 | 0.61 |  | 50 | 0.50 |
| 1101**00**10 | 0 | 51 | 0.47 |  | 57 | 0.47 |  | 45 | 0.38 |
| 11011**00**1 | 0 | 45 | 0.20 |  | 47 | 0.38 |  | 55 | 0.29 |
| **Streak Length = 3** |  | **281** | **0.65** |  | **319** | **0.66** |  | **272** | **0.72** |
| 01**000**101 | 0 | 40 | 0.65 |  | 55 | 0.78 |  | 50 | 0.80 |
| 010**111**01 | 1 | 53 | 0.55 |  | 44 | 0.64 |  | 41 | 0.73 |
| 0**111**0010 | 1 | 51 | 0.65 |  | 55 | 0.53 |  | 43 | 0.63 |
| 1**000**1010 | 0 | 49 | 0.65 |  | 64 | 0.72 |  | 52 | 0.65 |
| 11**000**110 | 1 | 41 | 0.51 |  | 48 | 0.52 |  | 41 | 0.66 |
| **111**01010 | 1 | 47 | 0.89 |  | 53 | 0.77 |  | 45 | 0.82 |
| **Streak Length = 4** |  | **206** | **0.75** |  | **220** | **0.79** |  | **179** | **0.75** |
| **0000**1101 | 0 | 54 | 0.72 |  | 54 | 0.80 |  | 47 | 0.66 |
| 010**1111**0 | 1 | 49 | 0.78 |  | 55 | 0.78 |  | 42 | 0.79 |
| 1**0000**101 | 0 | 59 | 0.71 |  | 46 | 0.76 |  | 44 | 0.80 |
| **1111**0010 | 1 | 44 | 0.80 |  | 65 | 0.80 |  | 46 | 0.78 |
| **Streak Length = 5** |  | **98** | **0.86** |  | **120** | **0.69** |  | **94** | **0.80** |
| 01**00000**1 | 0 | 52 | 0.83 |  | 61 | 0.67 |  | 48 | 0.67 |
| **11111**010 | 1 | 46 | 0.89 |  | 59 | 0.71 |  | 46 | 0.93 |
| **Streak Length = 6** |  | **99** | **0.71** |  | **102** | **0.66** |  | **98** | **0.76** |
| 1**000000**1 | 0 | 49 | 0.71 |  | 49 | 0.65 |  | 43 | 0.72 |
| **111111**01 | 1 | 50 | 0.70 |  | 53 | 0.66 |  | 55 | 0.78 |

*Note:* Focal streaks are highlighted in bold type in the first column of the table.

The only differences we might note between predictions for Filler sequences and predictions for Target sequences is that participants seem to update somewhat less in response to a streak that occurs earlier in the sequence (Fillers) than to streaks that occur at the end of the sequence (Targets).

# Chapter 4: Binary Logistic Regression Analysis

The following analysis uses a binary logistic regression model to estimate the population-average effect of Streak Length on the binary outcome variable Prediction, which takes the value “0” when participants predict that the 9th signal *will not match* the 8th signal in the sequence (the streak will *reverse*), and “1” when participants predict that the 9th signal *will match* the 8th signal (the streak will *repeat*). The analysis relies heavily on guidelines presented by Szmaragd, Clarke, and Steele (2013), and by Sommet and Morselli (2017).

Section 1 provides an explanation of the model. Section 2 presents the results of the analysis for Study 1B, where participants were not told the generator’s rate. Section 3 presents the results of the analysis for Study 2B, where participants were told the generator’s rate was fixed at .50. Section 4 presents the results of the analysis for Study 3B, where participants were told there was an equal chance (33%) that the generator took each of the following rates: .25, .50, or .75.

## Section 4.1: Explanation of the Binary Logistic Repeated Measures Model

If we fit repeated measures data using a standard linear model, the result would be one model equation defined for each participant at each Streak Length. This model assumes that all the residuals across these models are independent of each other, but the residuals in the models defined for a particular participant may in fact be correlated with one another. If we are interested in the population-average effect of Streak Length on Prediction, we can specify a population average model that takes this within-participant autocorrelation into account. Prediction is a binary variable taking the value “0” (if participants predict a streak will *reverse*) or “1” (if participants predict a streak will *repeat*). We estimate the average log-odds that Prediction = 1 (participant predicts streak will *repeat*) for participant *i* at Streak Length *t* as


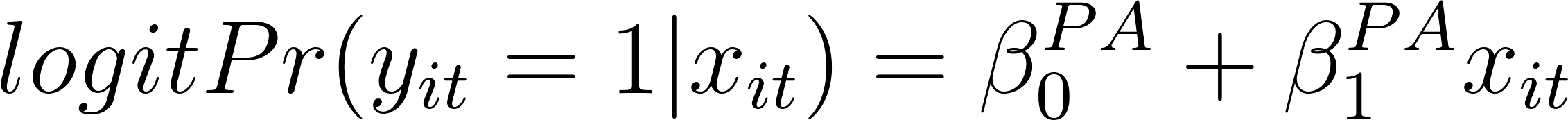


where *logitPr*(*y_it_* = 1|*x_it_*) is the average log-odds that Prediction = 1 among those participants with predictor variables *x_it_*. We can account for within-participant autocorrelation in our estimates of this model by specifying the autocorrelation between the residuals *e_i2_*, …, *e_i7_*, for each participant *i* and Streak Length 2,...,7. This autocorrelation structure is specified through the choice of a working correlation matrix.^^[[6]](#footnote-6)^^

Generalized Estimating Equations (GEE) allow us to estimate the logistic model while allowing for autocorrelation by specifying the structure of a working autocorrelation matrix.

GEE is a two-stage method in which the autocorrelation structure is treated as a nuisance to be adjusted for. Stage 1 of GEE involves estimating the ‘working correlation matrix’, the structure of which the user must specify prior to estimation; to specify this matrix correctly, the user must declare the occasion variable. Stage 2 of GEE uses the estimated working correlation matrix to adjust the estimates of the logistic model parameters and standard errors for autocorrelation (Szmaragd et al. 2013, p. 152).

In the subsequent analysis, we specify an unstructured correlation matrix. No assumption is made about the structure of the correlation matrix ­– the correlation between residuals *e_it_* and *e_is_* is allowed to vary for each pair of Streak Lengths *t*, *s*, where *t* ≠ *s*. In other words, the correlation between the residual terms in the models for participant *i* at Streak Lengths *t* and *s* is allowed to be different than that between all other pairs *t’*, *s’*.^^[[7]](#footnote-7)^^ The log odds of a participant predicting a streak will *repeat* (Prediction = 1) is then estimated as a function of Condition, Streak Length, and the interaction between Condition and Streak Length.^^[[8]](#footnote-8)^^

Throughout this analysis, we report the odds ratio (*OR*) as opposed to the log odds, for interpretability. We will refer to the odds ratio as the odds, following convention.

## Section 4.2: Study 1B

The results of the binary repeated measures logistic regression do not differ substantively from the results of the one-way mixed ANOVA. There was a significant main effect of Condition on Prediction (𝝌^2^(2) = 11.71, *p* = 0.003). Pairwise comparisons revealed the odds a participant in the BingoUnknown Condition predicts a streak will *repeat* were significantly lower than the odds a participant in the AnalystUnknown Condition does the same. However, after applying the Bonferroni correction for multiple comparisons, this difference was no longer significant (*OR*_BINGO/ANALYST_ = 0.65, *p*_UNADJUSTED_ = 0.034, *p*_BONFERRONI_ = 0.101). The odds that a participant in the BingoUnknown Condition predicts a streak will *repeat* were significantly lower than the odds that a participant in the StockUnknown Condition predicts *repeat*, and this difference remained significant after applying the Bonferroni correction (*OR*_BINGO/STOCK_ = 0.65, *p*_UNADJUSTED_ = 0.001, *p*_BONFERRONI_ = 0.003). No significant difference was found between the AnalystUnknown and StockUnknown Conditions (*OR*_STOCK/ANALYST_ = 1.31, *p*_UNADJUSTED_ = 0.222, *p*_BONFERRONI_ = 0.666).

The main effect of Streak Length was significant (𝝌^2^(5) = 153.42, *p* < 0.000). The odds a participant predicts a streak will *repeat* increase as Streak Length increases. Pairwise comparisons revealed only 4 cases where the odds do not increase significantly at longer streak lengths. The odds a participant predicts a streak will *repeat* are not significantly higher for streaks of length 6 than they are for streaks of length 5 (*OR*_STREAK=6/STREAK=5_ = 1.26, *p*_UNADJUSTED_ = 0.083), nor are they significantly higher for streaks of length 7 than they are for streaks of length 6 (*OR*_STREAK=7/STREAK=6_ = 1.15, *p*_UNADJUSTED_ = 0.340). After applying the Bonferroni correction, two additional pairs emerge as nonsignificant. The odds for streaks of length 4 are not significantly higher than for streaks of length 3 (*OR*_STREAK=4/STREAK=3_ = 1.40, *p*_BONFERRONI_ = 0.139), and the odds for streaks of length 7 are not significantly higher than streaks of length 5 (*OR*_STREAK=7/STREAK=5_ = 1.45, *p*_BONFERRONI_ = 0.205). At every other combination of Streak Lengths *t* and *t*-*n*, the odds that a participant predicts a streak will *repeat* are significantly higher (at the *p* < 0.001 level, after applying Bonferroni corrections) for streaks of length *t* than for streaks of length *t-n*. The interaction between Condition and Streak Length was not significant (𝝌^2^(10) = 12.44, *p* = 0.257).

## Section 4.3: Study 2B

The results of the binary repeated measures logistic regression do not differ substantively from the results of the one-way mixed ANOVA. There was a significant main effect of Condition on Prediction (𝝌^2^(2) = 18.07, *p* = 0.002). Pairwise comparisons revealed the odds a participant in the Bingo50 Condition predicts a streak will *repeat* are significantly lower than for a participant in the Analyst50 Condition (*OR*_BINGO/ANALYST_ = 0.55, *p*_UNADJUSTED_ = 0.004, *p*_BONFERRONI_ = 0.011). The odds that a participant in the Bingo50 Condition predicts a streak will *repeat* are also significantly lower than the odds that a participant in the Stock50 Condition predicts *repeat* (*OR*_BINGO/STOCK_ = 0.50, *p*_UNADJUSTED_ = 0.001, *p*_BONFERRONI_ = 0.003). No significant difference was found between the Analyst50 and Stock50 Conditions (*OR*_STOCK/ANALYST_ = 1.10, *p*_UNADJUSTED_ = 0.637, *p*_BONFERRONI_ = 0.100).

The main effect of Streak Length was significant (𝝌^2^(5) = 62.37, *p* < 0.000). The odds a participant predicts a streak will *repeat* increase as Streak Length increases, but only for streaks of 4 or longer. Pairwise comparisons revealed 5 cases where the odds do not increase significantly at longer streak lengths. The odds a participant predicts a streak will *repeat* are not significantly higher for streaks of length 3 than they are for streaks of length 2 (*OR*_STREAK=3/STREAK=2_ = 0.89, *p*_UNADJUSTED_ = 0.455). The odds are not significantly higher for streaks of length 6 than they are for streaks of length 5 (*OR*_STREAK=6/STREAK=5_ = 1.10, *p*_UNADJUSTED_ = 0.434). The odds for streaks of length 7 are not significantly higher than streaks of length 6 (*OR*_STREAK=7/STREAK=6_ = 0.96, *p*_UNADJUSTED_ = 0.337), and the odds for streaks of length 7 are not significantly higher than for streaks of length 5 (*OR*_STREAK=7/STREAK=5_ = 1.23, *p*_UNADJUSTED_ = 0.084). At every other combination of Streak Lengths *t* and *t*-*n*, the odds that a participant predicts a streak will *repeat* are significantly higher (at the *p* < 0.001 level, after applying Bonferroni) for streaks of length *t* than for streaks of length *t-n*.

The interaction between Condition and Streak Length was significant (𝝌^2^(10) = 25.02, *p* = 0.005). For streaks longer than 4 signals, the increase in the odds a participant predicts a streak will *repeat* is significantly lower in the Bingo50 than in the Analyst50 Condition. There is no significant difference between the Analyst50 and Stock50 Conditions.

## Section 4.4: Study 3B

The results of the binary repeated measures logistic regression do not differ substantively from the results of the one-way mixed ANOVA. The main effect of Condition on Prediction was not significant (𝝌^2^(2) = 1.23, *p* = 0.540). Pairwise comparisons revealed no significant difference between the odds a participant in the Bingo25-50-75 Condition predicts a streak will *repeat* versus a participant in the Analyst25-50-75 Condition (*OR*_BINGO/ANALYST_ = 1.09, *p*_UNADJUSTED_ = 0.661), or between the odds a participant in the Stock25-50-75 Condition predicts *repeat* versus a participant in the Analyst25-50-75 Condition (*OR*_STOCK/ANALYST_ = 1.25, *p*_UNADJUSTED_ = 0.270). No significant difference was found between the Bingo25-50-75 and Stock25-50-75 Conditions either (*OR*_STOCK/BINGO_ = 1.15, *p*_UNADJUSTED_ = 0.492).

The main effect of Streak Length was significant (𝝌^2^(5) = 66.07, *p* < 0.000). But, it seems this main effect is driven by the difference between predictions about streaks of length 2 versus every other Streak Length. Pairwise comparisons revealed that the odds a participant predicts streaks of length 3, 4, 5, 6, and 7 *repeat* are all significantly higher than the odds for streaks of length 2 (even after applying the Bonferroni correction for multiple comparisons). However, the odds are not significantly higher for streaks longer than 4 compared to shorter streaks (e.g. 4 versus 3, 5 versus 4, 7 versus 6). After an initial increase in odds between Streak Length 2 and 3, the odds a participant predicts a streak will *repeat* seem to stabilize, with no consistent pattern of increasing.

The interaction between Condition and Streak Length was not significant (𝝌^2^(10) = 15.92, *p* = 0.102). But, an inspection of the marginal linear predictions for the probability a participant predicts a streak will *repeat* show a subtly increasing pattern for the Stock25-50-75 and Analyst25-50-75 Conditions, while predictions in the Bingo25-50-75 Condition seem to stabilize for streaks longer than 3 (Figure OS13).

**Figure OS13**

*Marginal Linear Predictions of Probability Participant Predicts Repeat, by Condition and Streak Length*


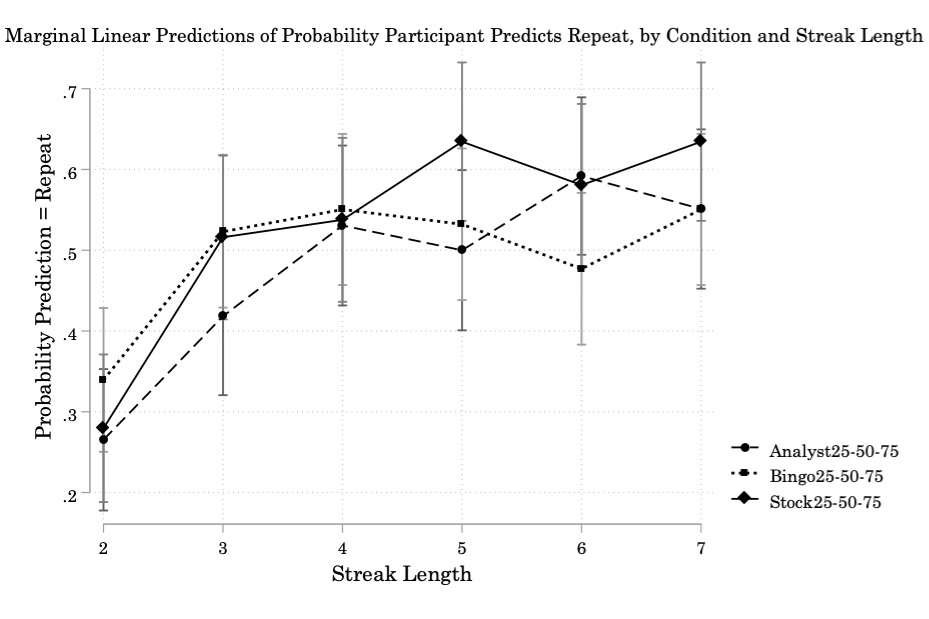


#

# Chapter 5: Strategic Heterogeneity in Reversal versus Repetition Patterns

## Section 5.1: Study 1A

Figure OS14 presents the distribution of fitted slopes for individual participants’ predictions over the target sequences. The distributions are centered above 5 in all three Conditions. On average, each incremental increase in the length of the terminal streak corresponds to an increase of about 5% in the probability participants assigned to repetition of that streak. The dominant strategy in all three Conditions is a positive slope, which we interpret as participants updating their beliefs about the base rate of the generators as Streak Length increased. A minority of participants exhibit negative slopes across their predictions, decreasing their expectations of repetition as Streak Length increased: 14% in the AnalystUnknown Condition, 7% in the StockUnknown Condition, and 20% in the BingoUnknown Condition. This pattern of predictions is consistent with gambler’s fallacy beliefs.

**Figure OS14**

*Study 1A: Distribution of Individual Participants’ Prediction Strategies*


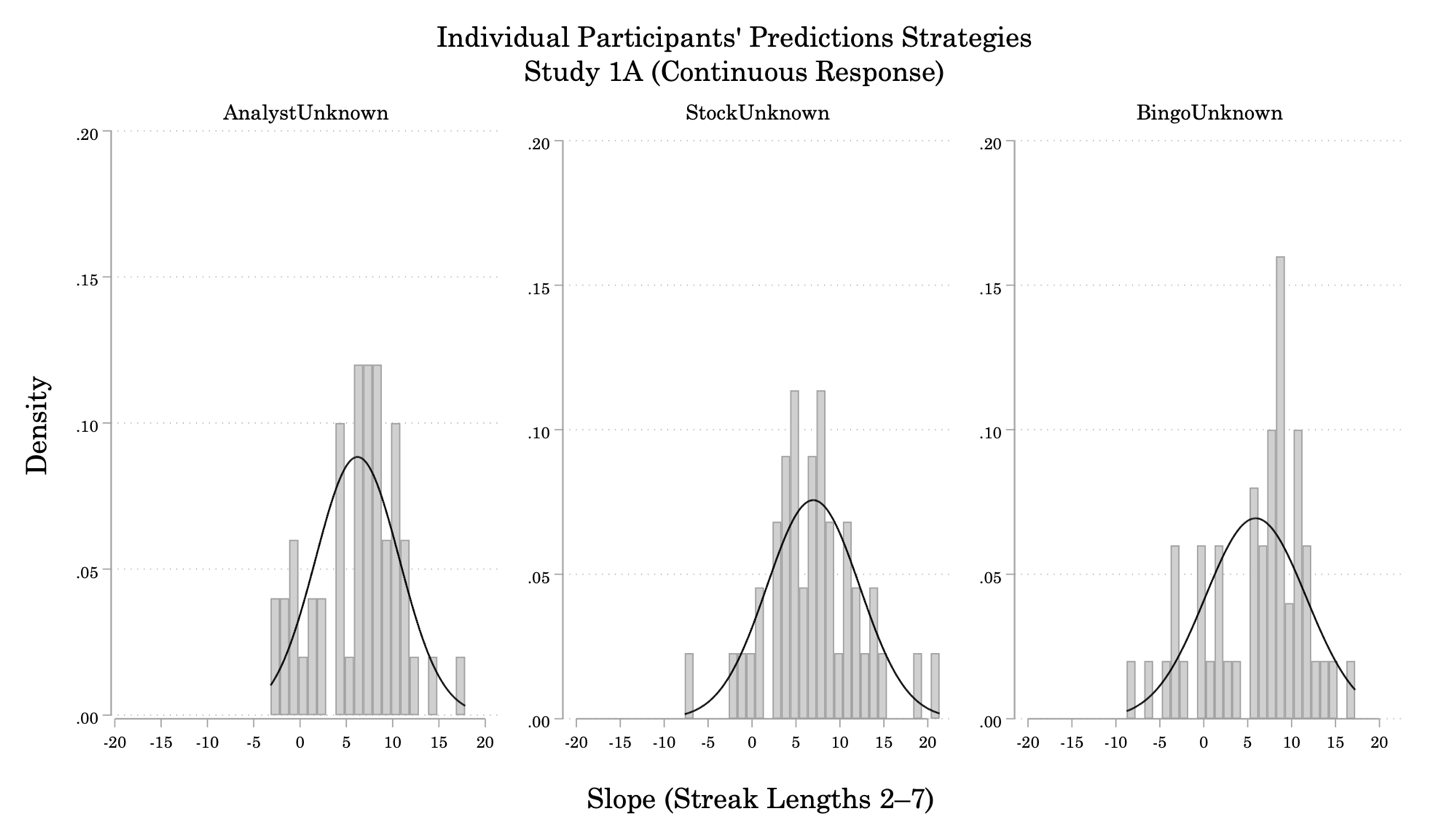


*Note:* Participants’ individual prediction strategies in Study 1A. N = 144 (StockUnknown = 44; AnalystUnknown = 50; BingoUnknown = 50). Histograms of frequencies for fitted slopes of participants’ predictions over target stimuli ending in streaks of length 2–7 (gray bars), overlaid with normal density curves (dark gray lines). The distributions of slopes for participants in the AnalystUnknown (left panel), StockUnknown (center panel), and BingoUnknown (right panel) Conditions are all centered above 5. For each additional signal added to the streak at the end of a target sequence, participants increased the probability they assigned to repetition of that streak by a little over 5%.

## Section 5.2: Study 1B

To get a sense of differences between individual participants’ prediction strategies we took a look at the coefficients obtained from logistic regressions fitted to each participant’s predictions over the target sequences ending in streaks of length 2–7.^[[9]](#footnote-9)^ For ease of exposition, we transformed the log odds coefficients obtained from these logistic regressions into the percent-change in the odds the participant predicts “repeat” for each unit increase in Streak Length.^[[10]](#footnote-10)^ A positive percent-change in the odds indicates that the likelihood the participant predicts repetition of the terminal streak increases as the length of that streak increases. A negative percent-change in the odds indicates the opposite strategy, that the likelihood the participant predicts repetition of the streak decreases as the length of that streak increases. Figure OS15 presents the distribution of individual participants’ strategies – the percent-change in the odds a participant predicts “repeat” for each unit increase in Streak Length.

**Figure OS15**

*Study 1B: Distribution of Individual Participants’ Prediction Strategies*


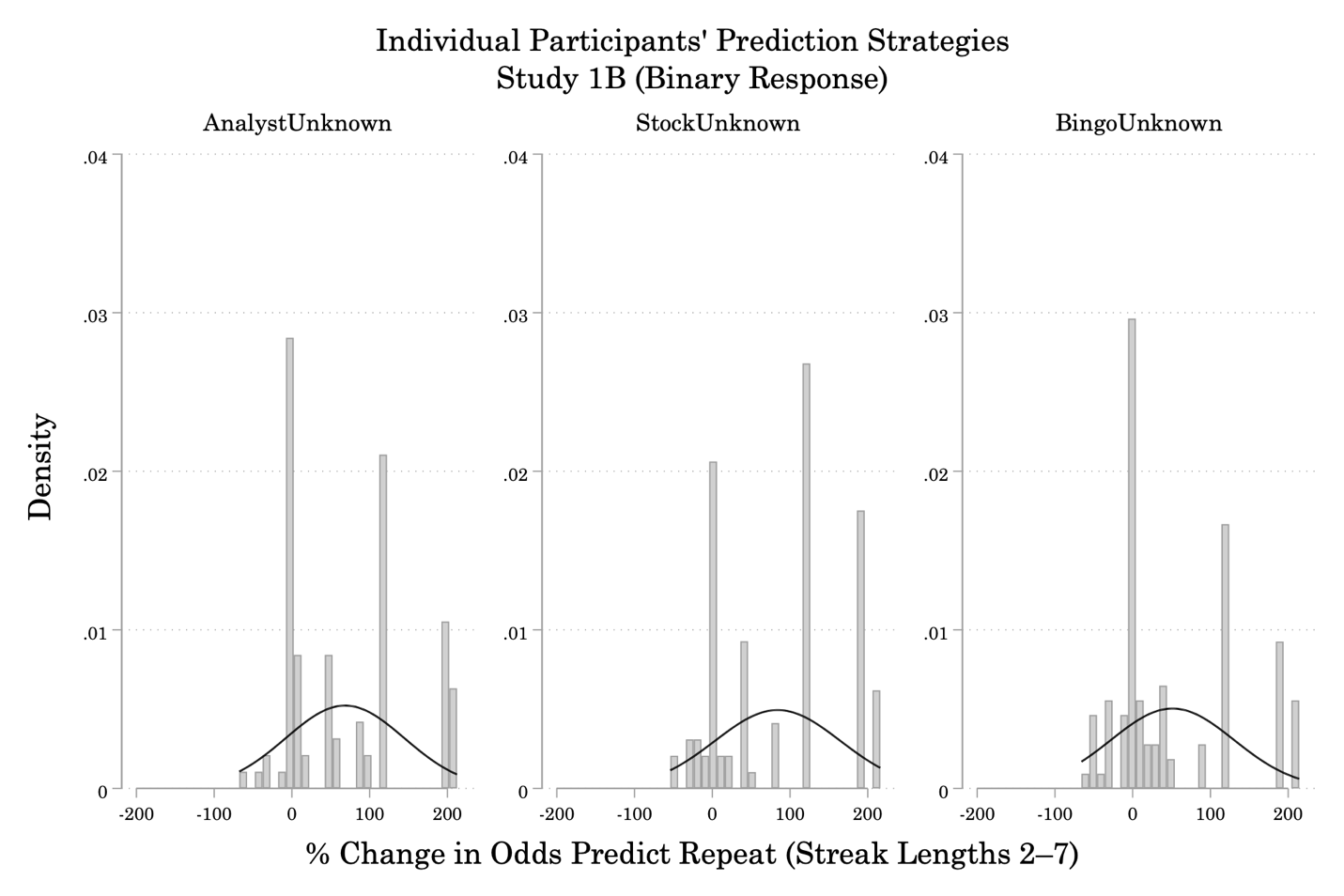


*Note:*  Individual participants’ prediction strategies in Study 1B. N = 300 (StockUnknown = 97; AnalystUnknown = 95; BingoUnknown = 108). Logistic regressions were fitted to individual participants’ predictions over Target stimuli ending in streaks of length 2–7 to find the percent-change in the odds a participant predicts a streak will repeat for each unit increase in the length of that streak. Histograms of frequencies for different values of percent-changes (gray bars), overlaid with normal density curves (dark gray lines). The distributions of percent-changes for participants in the AnalystUnknown (left panel) and StockUnknown (center panel) Conditions are centered above 75%, and the distribution for participants in the BingoUnknown (right panel) Condition is centered above 50%.

As in Study 1A, there was some heterogeneity in individual participants’ prediction strategies. The distributions of percent-changes are centered above 75% in the AnalystUnknown and StockUnknown Conditions. The odds participants in these Conditions predict repetition for a streak of length *x* are about 75% higher than the odds a participant in these Conditions predict repetition for a streak of length *x* – 1. The distribution of values is centered above 50% in the BingoUnknown Condition. A minority of participants exhibit negative percent-changes in the odds they will predict “repeat” as Streak Length increases: 8% in the AnalystUnknown Condition, 10% in the StockUnknown Condition, and 16% in the BingoUnknown Condition.

## Section 5.3: Study 2A

Again, we look at the slopes of linear regressions fitted to each participants’ predictions over the 6 target sequences to get a sense of the distribution of participant strategies (Figure OS16). Individual participants employed similar, albeit more conservative, updating strategies in the Analyst50 and Stock50 Conditions of Study 2A as they did in the AnalystUnknown and StockUnknown Conditions of Study 1A. But, we do see a difference between the prediction strategies employed in the Bingo50 Condition and the BingoUnknown Condition (compare Figures OS16 and OS14). There were more extreme outliers in both the left- and right-tails of the distribution in the Bingo50 Condition in Study 2A than there were in the BingoUnknown Condition of Study 1A. The predictions made by 54% of participants in the Bingo50 Condition in Study 2A exhibited a negative slope, compared to only 20% in the BingoUnknown Condition of Study 1A.

**Figure OS16**

*Study 2A: Distribution of Individual Participants’ Prediction Strategies*


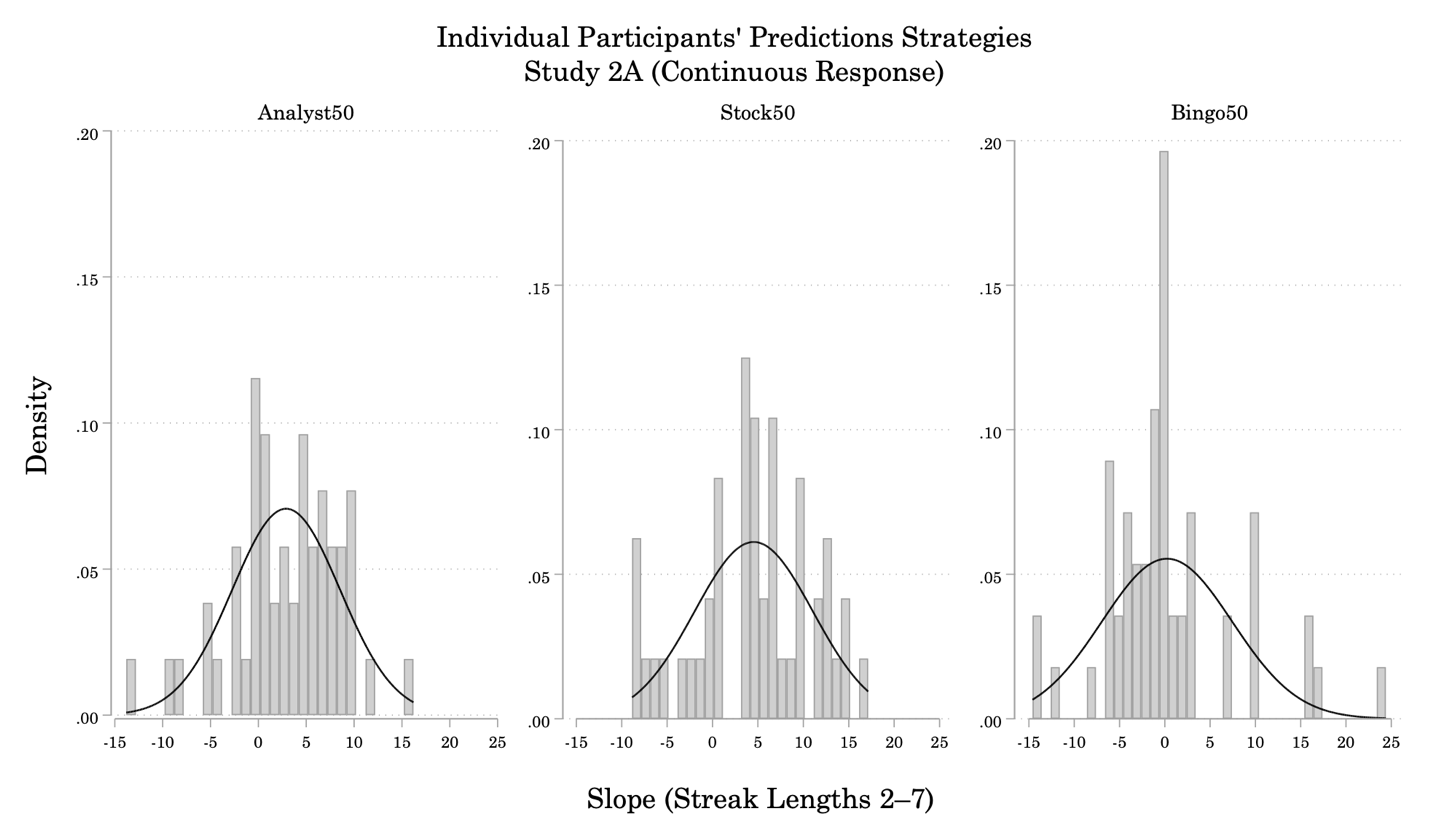


*Note:* Individual participants’ prediction strategies in Study 2A. N = 156 (Stock50= 48; Analyst50 = 52; Bingo50 = 56). Histograms of frequencies for fitted slopes of participants’ predictions over target stimuli ending in streaks of length 2–7 (gray bars), overlaid with normal density curves (dark gray lines). The distributions of slopes for participants in the Analyst50 (left panel) and Stock50 (center panel) Conditions are centered between 2.5 and 5. With each additional signal added to the streak at the end of a target sequence, participants increased the probability they assigned to repetition of that streak by 2.5 to 5%. The distribution of slopes for participants in the Bingo50 Condition (right panel) is centered above zero.

If we focus on the average results, we would conclude that participants given a stationary base rate for a random generator (Bingo50 Condition) show a bias to predict reversals, consistent with gambler’s fallacy reasoning. And, the expectation of reversal appears constant across Streak Lengths. In spite of the explicit instructions about a stationary base rate, participants faced with a market process (Stock50 Condition) or an intentional agent (Analyst50 Condition) generator seem to update their estimates of the base rate as the length of the streak increases to the point that the base rate provided in the instructions becomes implausible. (We interpret the fact that participants updated more conservatively in Study 2A than in Study 1A as an indication that the specific base rate instructions they received in 2A were at least somewhat compelling).

## Section 5.4: Study 2B

We again fitted logistic regressions to each participants’ predictions for the six target sequences ending in a streak.^[[11]](#footnote-11)^ We then transformed each participant’s coefficient from the log odds to the percent-change in the odds of predicting repetition for each unit increase in Streak Length. Figure OS17 presents the distributions of these values in each Condition. The distributions of percent-change values are centered above 50% in the Analyst50 and Stock50 Conditions, and the distribution of values is centered above 15% in the Bingo50 Condition. The odds that a participant in the Bingo50 Condition would predict repetition for a streak of length *x* were about 15% higher than the odds a participant in this Condition would predict repetition for a streak of length *x* – 1. A minority of participants exhibit negative percent-changes in the odds they predict “repeat” as Streak Length increases: 14% in the Analyst50 Condition, 13% in the Stock50 Condition, and 26% in the Bingo50 Condition.

**Figure OS17**

*Study 2B: Distribution of Individual Participants’ Prediction Strategies*


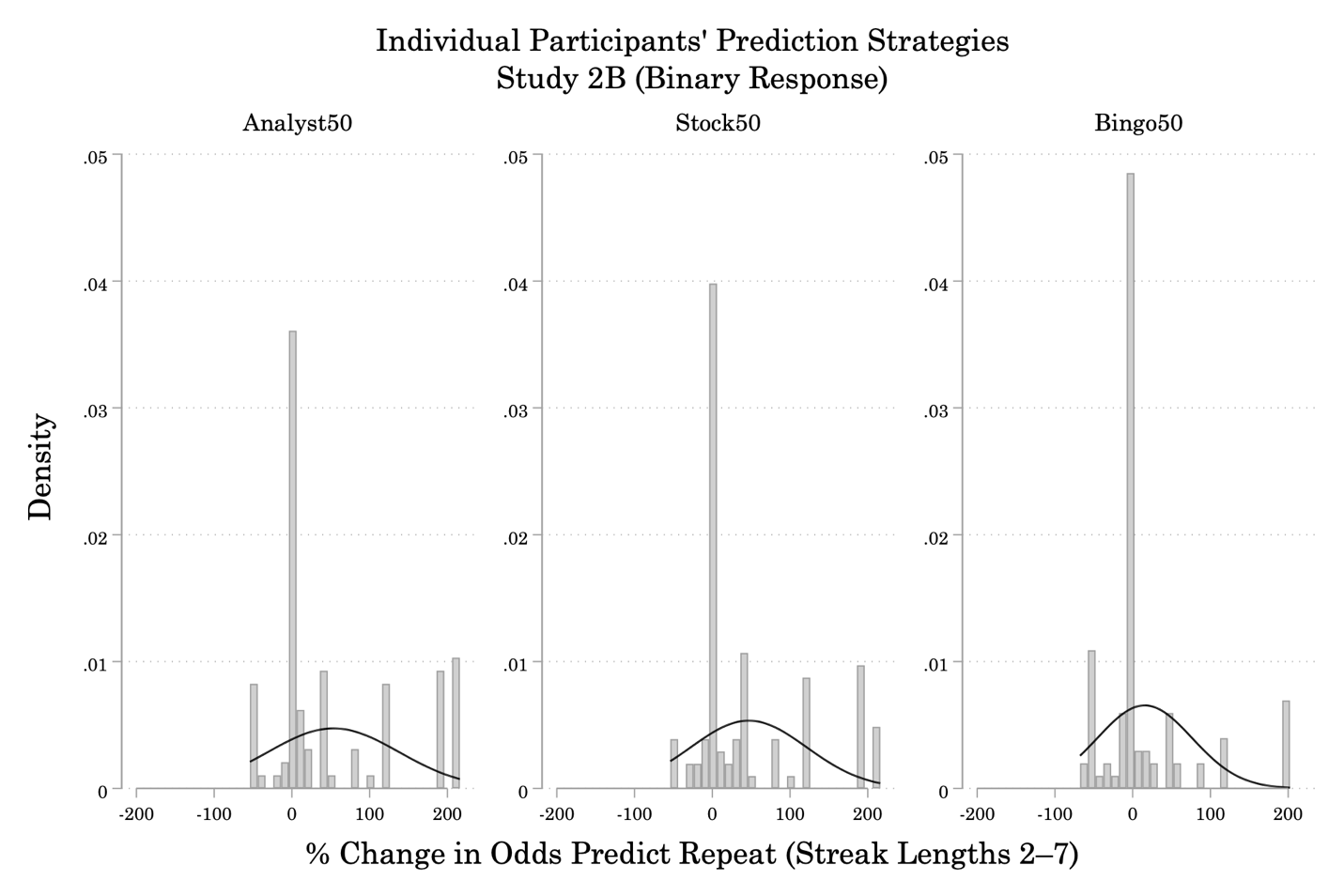


*Note:* Individual participants’ prediction strategies in Study 2B. N = 301 (Stock50 = 103; Analyst50 = 97; Bingo50 = 101). Logistic regressions were fitted to participants’ predictions over target stimuli ending in streaks of length 2–7 to find the percent-change in the odds a participant predicts a streak will repeat for each unit increase in the length of that streak. Histograms of frequencies for different values of percent-changes (gray bars), overlaid with normal density curves (dark gray lines). The distributions of percent-changes for participants in the Analyst50 and Stock50 Conditions are centered above 50%, and the distribution for participants in the Bingo50 Condition is centered above 15%.

##

## Section 5.5: Study 3A

Figure OS18 summarizes participants’ prediction strategies. The distribution of slopes from regressions fitted to each participant’s predictions over the target sequences are centered just above 5 in all three Conditions. For each unit increase in Streak Length, participants increased the probability they assigned to repetition of that streak by a little over 5%. There is more heterogeneity in prediction strategies used by participants in the Stock25-50-75 Condition than by those in the Analyst25-50-75 and Bingo25-50-75 Conditions.^[[12]](#footnote-12)^ There are also more extreme negative “outlier” strategies – participants whose expectations of repetition *decrease* as Streak Length increases – in the Bingo25-50-75 Condition than in the other two Conditions.

**Figure OS18**

*Study 3A: Distribution of Individual Participants’ Prediction Strategies*


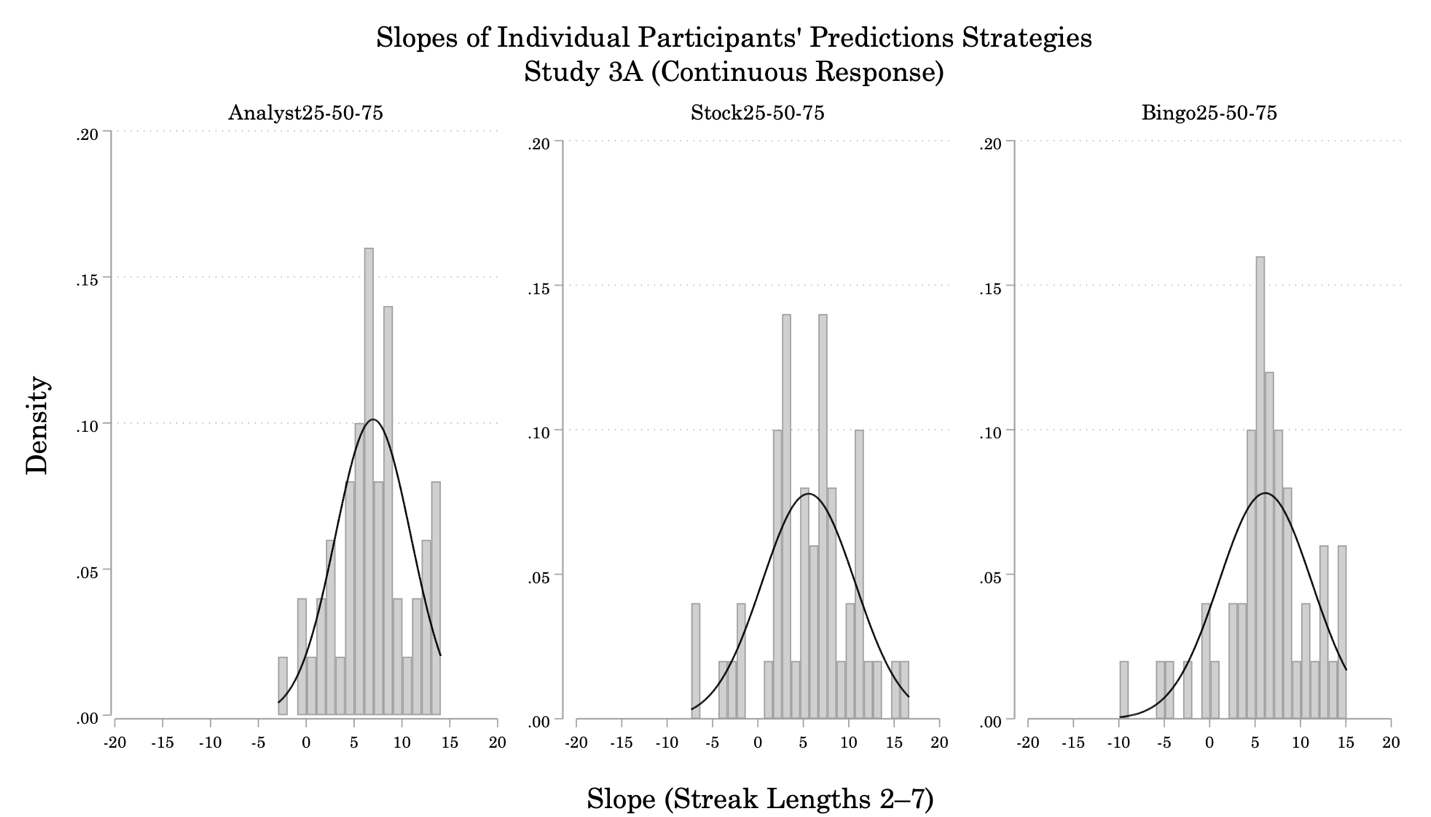


*Note:* Individual participants’ prediction strategies in Study 3A. N = 150 (Stock25-50-75 = 50; Analyst25-50-75 = 50; Bingo25-50-75 = 50). Histograms of frequencies for slopes of linear regressions fitted to each participant’s predictions over target stimuli (gray bars), overlaid with normal density curves (dark gray lines). Distributions of individual prediction slopes are centered just above 5 in all three Conditions. For each unit increase in the length of the terminal streak, participants increased the probability they assigned to the event that streak would repeat by a little over 5%.

## Section 5.6: Study 3B

We again fitted logistic regressions to each participant’s predictions for the six target sequences ending in a streak, and transformed the resulting coefficients from the log odds to the percent-change in the odds of predicting repetition for each unit increase in Streak Length.^[[13]](#footnote-13)^

**Figure OS19**

*Study 3B: Distribution of Individual Participant’s Prediction Strategies*


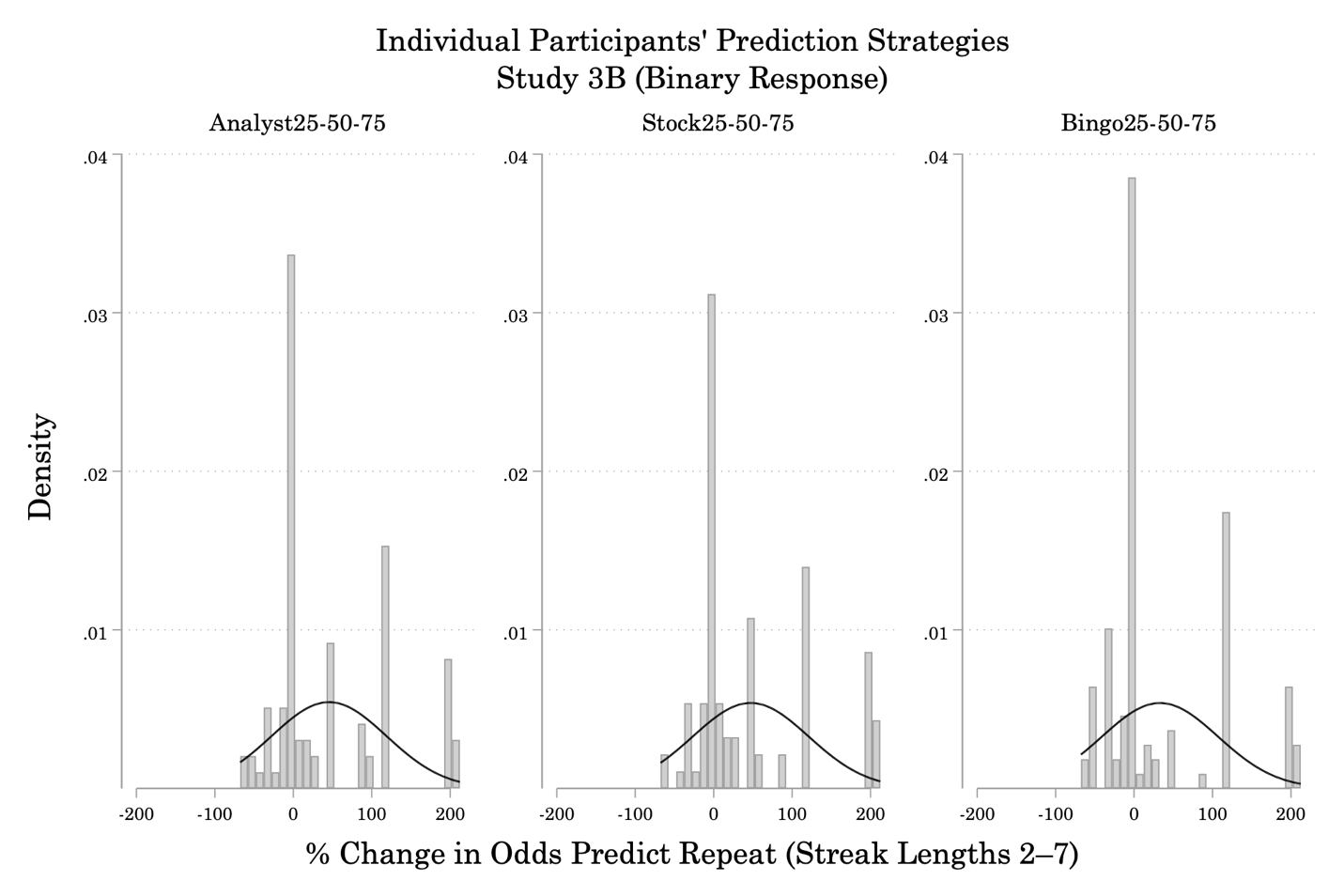


*Note:* Individual participants’ prediction strategies in Study 3B. N = 300 (Stock25-50-75 = 93; Analyst25-50-75 = 98; Bingo25-50-75 = 109). Logistic regressions were fitted to participants’ predictions over target stimuli ending in streaks of length 2–7 to find the percent-change in the odds a participant predicts a streak will repeat for each unit increase in the length of that streak. Histograms of frequencies for different values of percent-changes (gray bars), overlaid with normal density curves (dark gray lines). The distributions of percent-changes for participants in the Analyst25-50-75 and Stock25-50-75 Conditions are centered above 50%, and the distribution for participants in the Bingo25-50-75 Condition is centered above 25%.

Figure OS19 presents the distribution of participant strategies in each Condition. The distributions of percent-change values are centered above 50% in the Analyst50 and Stock50 Conditions, and the distribution of values is centered above 25% in the Bingo50 Condition. The odds that a participant in the Bingo25-50-75 Condition predicts repetition for a streak of length *x* were about 25% higher than the odds a participant in this Condition predicts repetition for a streak of length *x* – 1. A minority of participants exhibit negative percent-changes in the odds they will predict “repeat” as Streak Length increases: 18% in the Analyst25-50-75 Condition, 16% in the Stock25-50-75 Condition, and 28% in the Bingo25-50-75 Condition.

# Chapter 6: Individual Differences

After completing the experimental procedure, we collected additional information from each participant in the present studies, including demographics, responses to general knowledge questions, and participants’ qualitative descriptions of their own prediction strategies. In Section 6.1, we present an analysis of the relationship between individual differences and participants’ prediction strategies in each of the present studies. In Section 6.2, we present a summary of participants’ qualitative descriptions of their prediction strategies.

## Section 6.1: Individual Difference Measures and Participant Strategies

In this section, we consider the relationship between participants’ prediction strategies and individual difference measures. We first describe the individual difference measures collected, as well as statistics that capture participants’ prediction strategies. We then present descriptive statistics and pairwise correlations for each study separately. After completing the experimental procedure, participants were asked to respond to a brief demographics questionnaire, which asked for participants’ age, gender, and highest degree obtained. Three additional questions tested participants' familiarity with probability theory (Probability Questions), and two questions tested participants’ financial literacy (Financial Literacy Questions). The full text of the Probability and Financial Literacy Questions follows below.

***Probability Questions***

1. Suppose you have a fair coin. Each time you flip the coin, there is a 50% chance the coin lands son heads, and a 50% chance the coin lands on tails. What is the probability that the coin lands on heads three times in a row? (Question designed by authors of present article.)
2. The chance of getting a viral infection is 0.0005. Out of 10,000 people, about how many of them are expected to get a viral infection? (Adapted from Peters and colleagues, 2006. 56% of participants were able to answer this question correctly in Peters and colleagues’ study.)
3. In the Acme Publishing Sweepstakes, the chance of winning a car is 1 in 1,000. What percent of tickets in the Acme Publishing Sweepstakes win a car? 1%, 10%, or 0.1%? (Adapted from Peters and colleagues, 2006. 46% of participants were able to answer this question correctly in Peters and colleagues’ study.)

***Financial Literacy Questions***

1. Suppose you owe $1,000 on a loan and the interest rate you are charged is 20% per year compounded annually. If you didn’t pay anything off, at this interest rate, how many years would it take for the amount you owe to double?
    o Less than 2 years
    o At least 2 years but less than 5 years
    o At least 5 years but less than 10 years
    o At least 10 years
   (Adapted from FINRA 2015 survey. 75% of participants answered this question correctly on the FINRA survey.)
2. Fill in the blank. Buying a single company’s stock is usually __________ buying a stock mutual fund.
    o more safe than
    o the same as
    o less safe than
   (Adapted from FINRA 2015 survey. 46% of participants answered this question correctly on the FINRA survey.)

Participants were also asked for a subjective rating of their own knowledge of the stock market, and of the frequency with which they gamble.

1. How well do you feel you understand the stock market, compared to the average person?

o Better than average (coded as 1)

o About average (coded as 0)

o Worse than average (coded as –1)

2. How often do you feel you gamble, compared to the average person?

o More than average (coded as 1)

o About average (coded as 0)

o Less than average (coded as –1)

In addition to the individual difference measures collected at the end of the experimental procedure, we also calculated statistics that capture individual participants’ prediction strategies. For participants in the Continuous Versions of Studies 1–3 (1A, 2A, 3A), we calculated the average probability each participant assigned to repetition of the terminal streak at the end of each Target sequence (Average “Repeat”). We also performed individual participant-level linear regressions to estimate the coefficient (Slope) of Streak Length – the unit increase in the probability of repetition that participants assigned for each unit increase in Streak Length.

For participants in the Binary Versions of Studies 1–3 (1B, 2B, 3B), we summed each participant’s “Repeat” predictions across the Target sequences (Number “Repeat”). We also performed individual participant-level binary logistic regressions to estimate the coefficient (Slope) of Streak Length - the percent increase or decrease in the odds a participant predicts a streak will repeat for each unit increase in Streak Length. For example, a participant following a “hot hand” prediction strategy is more likely to predict repetition as Streak Length increases. The log odds (*β*) coefficient of Streak Length obtained from a binary logistic regression over such a participant’s predictions might be something like 1.07. In this case, the odds (*e^β^*) that this participant predicts a streak of length 3 will repeat would be 2.93 (*e*^1.07^) times greater than the odds that this participant predicts a streak of length 2 will repeat. The odds this participant predicts a streak of length *x* will repeat are thus 193% [100 ✕ (2.93–1)] greater than the odds this participant predicts a streak of length *x*–1 will repeat. (A detailed explanation of the interpretation of these coefficients is provided in Section 6.2) A brief summary of the individual difference measures, as well as the statistics we used to capture participants’ prediction strategies, can be found in Table OS9.

**Table OS9**

*Description of Individual Difference Measures and Statistics Summarizing Participants’ Prediction Strategies*

| AG: Age | Participant’s age, in years |
| --- | --- |
| FE: Female | Participant’s gender identity (1 = Female; 0 = Male/Other) |
| HD: Highest Degree | 1 = No Degree, 2 = High School Diploma, 3 = 2-Year College Degree or Skilled Trade Program, 4 = 4-year College Degree or higher |
| NP: # Accurate Probability | Number of probability questions answered correctly (out of 3) |
| NF: # Accurate Financial | Number of financial literacy questions answered correctly (out of 2) |
| KS: Knows Stocks | “Better than average” = 1; “About average” = 0;  “Worse than average” = ­–1 |
| GO: Gambles Often | “More than average” = 1; “About average” = 0; “Less than average” = –1 |
| AR: Average “Repeat” | **Studies 1A, 2A, 3A:** Mean probability assigned to repetition of terminal streak across Target sequences. |
| NR: Number “Repeat” | **Studies 1B, 2B, 3B:** Sum of “Repeat” (= 1) predictions across Target sequences. |
| SL: Slope | **Studies 1A, 2A, 3A:** Slope of individual participants’ linear regression lines, including predictions over streaks of length 1 (fillers), and of lengths 2, 3, 4, 5, 6, and 7 (targets). |
| SL: Slope | **Studies 1B, 2B, 3B:** The percent increase or decrease in the odds a participant predicts a streak will repeat for each unit increase in Streak Length. |

### Section 6.1.1: Study 1A Results

In Study 1A, none of the individual difference measures were significantly correlated with either of the statistics capturing participants’ prediction strategies (AR: Average Repeat, SL: Slope). Table OS10 presents the summary statistics for individual difference measures and participants’ prediction strategies. Table OS11 presents the Pearson Product Moment Correlation Matrix for these variables.

**Table OS10**

*Study 1A: Summary Statistics for Individual Difference Measures and Prediction Strategies*

|  | AnalystUnknown | StockUnknown | BingoUnknown |
| --- | --- | --- | --- |
| Mean Age | 34.74 (11.06) | 37.43 (13.15) | 36.06 (9.14) |
| % Female | 0.42 | 0.36 | 0.42 |
| Mean Highest Degree | 3.36 (0.84) | 3.07 (0.99) | 2.98 (0.91) |
| Mean # Accurate Probability | 1.68 (0.95) | 1.66 (1.07) | 1.68 (1.01) |
| Mean # Accurate Financial | 1.42 (0.64) | 1.46 (0.62) | 1.54 (0.64) |
| Mean “Know Stocks” | –0.02 (0.69) | –0.09 (0.63) | –0.26 (0.66) |
| Mean “Gambles Often” | –0.46 (0.67) | –0.61 (0.53) | –0.50 (0.54) |
| Mean Average Repeat | 66% (16%) | 70% (14%) | 60% (19%) |
| Mean Slope | 6.20 (4.45) | 6.97 (5.22) | 5.92 (5.70) |
| N | 50 | 44 | 50 |
| *Note: Numbers in parentheses are standard deviations* | | | |

**Table OS11**

*Study 1A: Pearson Product Moment Correlation Matrix for Variables of Interest*

|  | AG | FE | HD | NP | NF | KS | GO | AR | SL |
| --- | --- | --- | --- | --- | --- | --- | --- | --- | --- |
| AG: Age |  | 0.23 | 0.03 | 0.02 | 0.08 | –0.00 | –0.25 | –0.01 | 0.09 |
| FE: Female |  |  | –0.03 | **–0.30**** | –0.12 | **–0.31**** | 0.05 | –0.14 | –0.00 |
| HD: Highest Degree |  |  |  | 0.18 | 0.10 | 0.11 | 0.11 | –0.02 | 0.04 |
| NP: # Acc. Probability |  |  |  |  | **0.30**** | 0.07 | –0.19 | 0.14 | 0.12 |
| NF: # Acc. Financial |  |  |  |  |  | –0.07 | –0.12 | 0.04 | 0.11 |
| KS: Know Stocks |  |  |  |  |  |  | 0.24 | 0.04 | –0.23 |
| GO: Gambles Often |  |  |  |  |  |  |  | –0.16 | –0.10 |
| AR: Average Repeat |  |  |  |  |  |  |  |  | **0.31**** |
| SL: Slope |  |  |  |  |  |  |  |  |  |

*Note:* The Pearson product moment correlation evaluates the linear relationship between two continuous variables.  It assumes two variables change together at a constant rate.  This measure is not appropriate for comparing a continuous measure (Slope) with non-continuous measures (e.g. Highest Degree).  The alternative Spearman rank-order correlation is also inappropriate because we are comparing a continuous variable (Slope) with ordinal and categorical variables (e.g. Female).  The above table is only provided to give the reader a sense of the global patterns we observed in the data. * *p* < 0.05, ** *p* < 0.01, *** *p* < 0.001, with Bonferroni correction for multiple comparisons.

### Section 6.1.2: Study 1B Results

In Study 1B, there was a small positive correlation between subjective ratings of stock market knowledge and the number of times participants predicted streaks would repeat (Table OS13). None of the other individual difference measures were significantly correlated with either of the statistics capturing participants’ prediction strategies (NR: Number Repeat, SL: Slope).

**Table OS12**

*Study 1B: Summary Statistics for Individual Difference Measures and Prediction Strategies*

|  | AnalystUnknown | StockUnknown | BingoUnknown |
| --- | --- | --- | --- |
| Mean Age | 34.05 (10.60) | 36.37 (13.93) | 35.80 (10.65) |
| % Female | 0.42 | 0.54 | 0.47 |
| Mean Highest Degree | 3.43 (0.81) | 3.23 (0.95) | 3.38 (0.89) |
| Mean # Accurate Probability | 1.92 (0.95) | 2.08 (0.92) | 1.97 (1.21) |
| Mean # Accurate Financial | 1.46 (0.67) | 1.41 (0.67) | 1.43 (0.64) |
| Mean “Know Stocks” | –0.02 (0.58) | –0.08 (0.62) | –0.15 (0.68) |
| Mean “Gambles Often” | –0.45 (0.66) | –0.46 (0.68) | –0.50 (0.70) |
| Mean Number Repeat | 3.72 (1.84) | 4.00 (1.68) | 3.15 (2.02) |
| Mean Slope (% Δ Odds) | 68% (76%) | 84% (81%) | 51% (79%) |
| N | 95 | 97 | 108 |

*Note:* Numbers in parentheses are standard deviations. Slope: [100 ✕ (*e^β^*–1)] The percent change in the odds that a participant predicts a streak of length *x* will repeat versus a streak of length *x*–1. Separation was observed while running participant-level logistic regressions on 127/300 participants’ predictions over target sequences.  Firth’s procedure was applied to all of the participant-level logistic regressions to resolve the separation issue, producing less biased coefficients (see Heinze & Schemper, 2002).

**Table OS13**

*Study 1B: Pearson Product Moment Correlation Matrix for Variables of Interest*

|  | AG | FE | HD | NP | NF | KS | GO | NR | SL |
| --- | --- | --- | --- | --- | --- | --- | --- | --- | --- |
| AG: Age |  | **0.23**** | 0.11 | –0.10 | 0.09 | 0.01 | –0.18 | –0.04 | 0.01 |
| FE: Female |  |  | 0.12 | –0.15 | –0.04 | **–0.34***** | **–0.28***** | –0.13 | 0.02 |
| HD: Highest Degree |  |  |  | 0.09 | 0.07 | 0.14 | –0.05 | 0.10 | 0.07 |
| NP: # Acc. Probability |  |  |  |  | **0.33***** | 0.17 | 0.01 | 0.14 | 0.03 |
| NF: # Acc. Financial |  |  |  |  |  | 0.14 | –0.12 | 0.14 | 0.12 |
| KS: Know Stocks |  |  |  |  |  |  | **0.22**** | **0.21**** | 0.00 |
| GO: Gambles Often |  |  |  |  |  |  |  | 0.02 | –0.11 |
| NR: Number Repeat |  |  |  |  |  |  |  |  | 0.04 |
| SL: Slope |  |  |  |  |  |  |  |  |  |
| * *p* < 0.05, ** *p* < 0.01, *** *p* < 0.001, with Bonferroni correction for multiple comparisons. | | | | | | | | | |

### Section 6.1.3: Study 2A Results

In Study 2A, none of the individual difference measures were significantly correlated with either of the statistics capturing participants’ prediction strategies (Table OS15).

**Table OS14**

*Study 2A:* *Summary Statistics for Individual Difference Measures and Prediction Strategies*

|  | Analyst50 | Stock50 | Bingo50 |
| --- | --- | --- | --- |
| Mean Age | 34.06 (9.29) | 36.52 (9.46) | 36.16 (11.69) |
| % Female | 0.44 | 0.46 | 0.43 |
| Mean Highest Degree | 3.04 (0.84) | 3.19 (0.94) | 3.23 (0.85) |
| Mean # Accurate Probability | 1.98 (0.96) | 2.02 (0.98) | 1.82 (1.16) |
| Mean # Accurate Financial | 1.42 (0.61) | 1.56 (0.62) | 1.30 (0.74) |
| Mean “Know Stocks” | ﹣0.19 (0.60) | ﹣0.13 (0.57) | 0.09 (0.69) |
| Mean “Gambles Often” | ﹣0.52 (0.73) | ﹣0.54 (0.68) | ﹣0.41 (0.68) |
| Mean Average Repeat | 55% (20%) | 58% (20%) | 44% (18%) |
| Mean Slope | 2.90 (5.65) | 4.54 (6.52) | 0.24 (7.20) |
| N | 52 | 48 | 56 |
| *Note*: Numbers in parentheses are standard deviations | | | |

**Table OS15**

*Study 2A: Pearson Product Moment Correlation Matrix for Variables of Interest*

|  | AG | FE | HD | NP | NF | KS | GO | AR | SL |
| --- | --- | --- | --- | --- | --- | --- | --- | --- | --- |
| AG: Age |  | 0.17 | 0.02 | –0.09 | 0.07 | –0.06 | –0.17 | –0.03 | –0.02 |
| FE: Female |  |  | 0.02 | **–0.34***** | **–0.30**** | –0.21 | –0.04 | –0.03 | 0.00 |
| HD: Highest Degree |  |  |  | 0.11 | –0.07 | 0.19 | 0.02 | –0.03 | –0.04 |
| NP: # Acc. Probability |  |  |  |  | **0.49***** | 0.17 | 0.01 | –0.04 | –0.09 |
| NF: # Acc. Financial |  |  |  |  |  | 0.09 | –0.11 | –0.12 | –0.12 |
| KS: Know Stocks |  |  |  |  |  |  | **0.43***** | 0.00 | –0.10 |
| GO: Gambles Often |  |  |  |  |  |  |  | 0.04 | 0.07 |
| AR: Average Repeat |  |  |  |  |  |  |  |  | **0.58***** |
| SL: Slope |  |  |  |  |  |  |  |  |  |
| * *p* < 0.05, ** *p* < 0.01, *** *p* < 0.001, with Bonferroni correction for multiple comparisons. | | | | | | | | | |

### Section 6.1.4: Study 2B Results

In Study 2B, none of the individual difference measures were significantly correlated with either of the statistics capturing participants’ prediction strategies (Table OS17).

**Table OS16**

*Study 2B: Summary Statistics for Individual Difference Measures and Prediction Strategies*

|  | Analyst50 | Stock50 | Bingo50 |
| --- | --- | --- | --- |
| Mean Age | 37.93 (13.33) | 33.77 (11.83) | 35.17 (12.14) |
| % Female | 0.51 | 0.44 | 0.47 |
| Mean Highest Degree | 3.45 (0.83) | 3.27 (0.95) | 3.18 (0.97) |
| Mean # Accurate Probability | 2.07 (0.92) | 1.85 (0.83) | 1.94 (0.91) |
| Mean # Accurate Financial | 1.54 (0.61) | 1.47 (0.62) | 1.32 (0.71) |
| Mean “Know Stocks” | –0.15 (0.58) | –0.23 (0.60) | –0.17 (0.63) |
| Mean “Gambles Often” | –0.60 (0.62) | –0.44 (0.71) | –0.55 (0.66) |
| Mean Number Repeat | 2.35 (1.93) | 2.48 (2.10) | 1.54 (1.67) |
| Mean Slope (% Δ Odds) | 53% (84%) | 47% (75%) | 16% (61%) |
| N | 97 | 103 | 101 |
| *Note:* Numbers in parentheses are standard deviations | | | |

**Table OS17**

*Study 2B: Pearson Product Moment Correlation Matrix for Variables of Interest*

|  | | AG | FE | HD | NP | NF | KS | GO | NR | SL |
| --- | --- | --- | --- | --- | --- | --- | --- | --- | --- | --- |
| AG: Age | |  | 0.08 | 0.16 | 0.02 | 0.03 | –0.02 | –0.12 | –0.10 | 0.03 |
| FE: Female | |  |  | –0.02 | **–0.24***** | –0.15 | –0.30 | –0.16 | 0.04 | 0.04 |
| HD: Highest Degree | |  |  |  | 0.06 | 0.09 | **0.23**** | 0.04 | 0.06 | –0.03 |
| NP: # Acc. Probability | |  |  |  |  | **0.40***** | 0.14 | –0.03 | –0.09 | 0.03 |
| NF: # Acc. Financial | |  |  |  |  |  | 0.11 | –0.12 | –0.03 | 0.03 |
| KS: Know Stocks | |  |  |  |  |  |  | **0.19*** | 0.04 | 0.06 |
| GO: Gambles Often | |  |  |  |  |  |  |  | –0.01 | 0.02 |
| NR: Number Repeat | |  |  |  |  |  |  |  |  | **0.32***** |
| SL: Slope | |  |  |  |  |  |  |  |  |  |
| * *p* < 0.05, ** *p* < 0.01, *** *p* < 0.001, with Bonferroni correction for multiple comparisons. | | | | | | | | | |  |

### Section 6.1.5: Study 3A Results

In Study 3A, none of the individual difference measures were significantly correlated with either of the statistics capturing participants’ prediction strategies (Table OS19).

**Table OS18**

*Study 3A: Summary Statistics for Individual Difference Measures and Prediction Strategies*

|  | Analyst25-50-75 | Stock25-50-75 | Bingo25-50-75 |
| --- | --- | --- | --- |
| Mean Age | 31.90 (7.68) | 35.94 (12.35) | 34.42 (9.37) |
| % Female | 0.50 | 0.48 | 0.50 |
| Mean Highest Degree | 3.30 (0.84) | 3.26 (0.90) | 3.32 (0.82) |
| Mean # Accurate Probability | 1.98 (1.04) | 1.86 (0.88) | 1.90 (0.86) |
| Mean # Accurate Financial | 1.48 (0.65) | 1.52 (0.58) | 1.38 (0.67) |
| Mean “Know Stocks” | –0.12 (0.69) | –0.04 (0.60) | –0.24 (0.59) |
| Mean “Gambles Often” | –0.54 (0.68) | –0.46 (0.61) | –0.38 (0.75) |
| Mean Average Repeat | 72% (12%) | 65% (19%) | 65% (15%) |
| Mean Slope | 6.99 (3.94) | 5.58 (5.12) | 6.13 (5.11) |
| N | 50 | 50 | 50 |
| *Note:* Numbers in parentheses are standard deviations. | | | |

**Table OS19**

*Study 3A: Pearson Product Moment Correlation Matrix for Variables of Interest*

|  | AG | FE | HD | NP | NF | KS | GO | AR | SL |
| --- | --- | --- | --- | --- | --- | --- | --- | --- | --- |
| AG: Age |  | 0.03 | –0.04 | 0.01 | 0.18 | 0.12 | –0.05 | –0.06 | –0.02 |
| FE: Female |  |  | 0.18 | –0.10 | –0.19 | –0.19 | –0.16 | –0.02 | –0.05 |
| HD: Highest Degree |  |  |  | 0.04 | –0.02 | 0.22 | 0.03 | –0.07 | 0.03 |
| NP: # Acc. Probability |  |  |  |  | **0.32**** | 0.18 | –0.05 | 0.05 | –0.11 |
| NF: # Acc. Financial |  |  |  |  |  | 0.10 | –0.14 | 0.12 | –0.08 |
| KS: Know Stocks |  |  |  |  |  |  | **0.32**** | –0.01 | –0.04 |
| GO: Gambles Often |  |  |  |  |  |  |  | –0.13 | –0.11 |
| AR: Average Repeat |  |  |  |  |  |  |  |  | 0.24 |
| SL: Slope |  |  |  |  |  |  |  |  |  |
| * *p* < 0.05, ** *p* < 0.01, *** *p* < 0.001, with Bonferroni correction for multiple comparisons. | | | | | | | | | |

### Section 6.1.6: Study 3B Results

In Study 3B, none of the individual difference measures were significantly correlated with either of the statistics capturing participants’ prediction strategies (Table OS21).

**Table OS20**

*Study 3B: Summary Statistics for Individual Difference Measures and Prediction Strategies*

|  | Analyst50 | Stock50 | Bingo50 |
| --- | --- | --- | --- |
| Mean Age | 36.09 (12.11) | 36.85 (11.83) | 37.46 (12.53) |
| % Female | 0.44 | 0.54 | 0.60 |
| Mean Highest Degree | 3.19 (0.92) | 3.23 (0.88) | 3.23 (0.96) |
| Mean # Accurate Probability | 1.90 (0.94) | 2.02 (0.93) | 1.94 (1.00) |
| Mean # Accurate Financial | 1.44 (0.66) | 1.41 (0.65) | 1.36 (0.62) |
| Mean “Know Stocks” | –0.17 (0.66) | –0.15 (0.64) | –0.23 (0.73) |
| Mean “Gambles Often” | –0.58 (0.61) | –0.73 (0.51) | –0.68 (0.58) |
| Mean Number Repeat | 2.86 (2.00) | 3.18 (2.00) | 2.97 (2.12) |
| Mean Slope (% Δ Odds) | 46% (73%) | 47% (74%) | 33% (74%) |
| N | 98 | 93 | 109 |
| *Note*: Numbers in parentheses are standard deviations. | | | |

**Table OS21**

*Study 3B: Pearson Product Moment Correlation Matrix for Variables of Interest*

|  | | AG | FE | HD | | NP | NF | KS | GO | NR | SL |
| --- | --- | --- | --- | --- | --- | --- | --- | --- | --- | --- | --- |
| AG: Age | |  | 0.14 | 0.08 | | –0.16 | –0.06 | 0.04 | –0.05 | –0.07 | 0.05 |
| FE: Female | |  |  | –0.02 | | **–0.24**** | –0.15 | **–0.37***** | –0.20* | –0.10 | 0.03 |
| HD: Highest Degree | |  |  |  | | 0.14 | 0.05 | 0.16 | 0.00 | –0.11 | –0.02 |
| NP: # Acc. Probability | |  |  |  | |  | **0.32***** | **0.28***** | 0.09 | 0.05 | 0.03 |
| NF: # Acc. Financial | |  |  |  | |  |  | 0.18 | 0.04 | 0.05 | 0.04 |
| KS: Know Stocks | |  |  |  | |  |  |  | **0.20*** | 0.04 | –0.03 |
| GO: Gambles Often | |  |  |  | |  |  |  |  | 0.06 | –0.07 |
| NR: Number Repeat | |  |  |  | |  |  |  |  |  | **0.25***** |
| SL: Slope |  | |  | |  |  |  |  |  |  |  |
| * *p* < 0.05, ** *p* < 0.01, *** *p* < 0.001, with Bonferroni correction for multiple comparisons. | | | | | | | | | | | |

## Section 6.2: Interpretation of Binary Logistic Regression Coefficients

The independent variable coefficients obtained from a logistic regression can be interpreted as the “change in the log odds that (*Y* = 1) for each unit increase in [independent_variable],” where *Y* is the response variable. In the current application, *Y* takes the value 1 when the participant predicts a streak will **repeat**, and 0 otherwise. Exponentiating the log odds coefficients produces the odds ratios, which can be interpreted as the “change in the odds that (*Y* = 1) for each unit increase in [independent_variable].” The odds ratios are difficult to interpret when there is a negative relationship between the odds ratio and a unit increase in the independent variable. Transforming the odds ratios into the percent-change in the odds makes it easier to interpret both positive and negative relationships between the odds ratio and the independent variable. To obtain the percent change in the odds for a unit increase in the independent variable, we first subtract 1 from the odds ratio, and then we multiply the result by 100.

Example: For Participant A, the log odds that this participant predicts “repeat” are –0.78 higher for each unit increase in Streak Length. The odds that this participant predicts a streak will repeat are exp(–0.78) = 0.46 times higher for each unit increase in Streak Length. The percent-change in the odds this participant predicts “repeat” is [100 ✕ (0.46–1)] = –54% for each unit increase in Streak Length. This means that the odds this particular participant predicts “repeat” *decrease* as Streak Length increases.

The following presents a toy example for 2 contrived cases. Participant 1 is *more* likely to predict “repeat” as Streak Length increases (Table OS22). Participant 1 has a “growing” logistic function that *increases* from 0 to 1.

**Table OS22**

*Toy Example: Imaginary Participant 1's Responses to Stimuli of Varying Streak Length*

| Participant ID | Streak Length | Prediction  (0 = Reverse; 1 = Repeat) |
| --- | --- | --- |
| 1 | 2 | 0 |
| 1 | 3 | 1  1 |
| 1 | 4 | 1 |
| 1 | 5 | 1 |
| 1 | 6 | 1 |
| 1 | 7 | 1 |

When we regress Prediction on Streak Length for Participant 1, we obtain a log odds coefficient of 0.78. Exponentiating this coefficient, we obtain an odds ratio of 2.17. After subtracting 1 from the odds ratio, and then multiplying the result by 100, we obtain a percent-change of 117% in the odds for a 1 unit increase in Streak Length. The substantive interpretation of the percent change in the odds of predicting repetition for Participant 1 is as follows:

“The odds that this participant predicts a streak will repeat increase by 117% for each unit increase in Streak length.” – or – “The odds that this participant predicts a streak of length *x* will repeat are about 117% greater than the odds this participant predicts a streak of length *x*–1 will repeat.”

Participant 2 is *less* likely to predict “repeat” as Streak Length increases (Table OS23). Participant 2 has a “decaying” logistic function that *decreases* from 1 to 0.

**Table OS23**

*Toy Example: Imaginary Participant 2's Responses to Stimuli of Varying Streak Length*

| Participant ID | Streak Length | Prediction  (0 = Reverse; 1 = Repeat) |
| --- | --- | --- |
| 2 | 2 | 1 |
| 2 | 3 | 1 |
| 2 | 4 | 1 |
| 2 | 5 | 0 |
| 2 | 6 | 0 |
| 2 | 7 | 0 |

When we regress Prediction on Streak Length for Participant 2, we obtain a log odds coefficient of –1.13. Exponentiating this coefficient, we obtain an odds ratio of 0.32. After subtracting 1 from the odds ratio, and then multiplying the result by 100, we obtain a percent-change of –68% in the odds for a 1 unit increase in Streak Length. The substantive interpretation of the percent change in the odds of predicting repetition for Participant 2 is as follows:

“The odds that this participant predicts a streak will repeat decrease by –68% for each unit increase in Streak Length.” - or - “The odds that this participant predicts a streak of length *x* will repeat are about –68% less than the odds this participant predicts a streak of length *x–*1 will repeat.”

# References

FINRA Foundation. (2015). The National Financial Capability Study (NFCS): A project of the FINIRA Investor Education Foundation (FINRA Foundation). Retrieved from: http://www.usfinancialcapability.org/downloads.php

Heinze, G. and Schemper, M. (2002). A solution to the problem of separation in logistic regression. *Statistics in Medicine, 21*(16), 2409–2419.

Peters, E., Västfjäll, D., Slovic, P., Mertz, C.K., Mazzocco, K., and Dickert, S. (2006). Numeracy and Decision Making. *Psychological Science, 17(5)*, 407-413.

Sommet, Nicolas, and David Morselli. (2017). Keep Calm and Learn Multilevel Logistic Modeling: A Simplified Three-Step Procedure Using Stata, R, Mplus, and SPSS. *International Review of Social Psychology, 30*(1), 203–218.

Szmaragd, Camille, Paul Clarke, and Fiona Steele. (2013). Subject specific and population average models for binary longitudinal data: a tutorial. *Longitudinal and life course studies, 42*(2), 147-165.

1. The slider selector button was hidden, so participants had to click on the slider range to make it appear. This precaution was taken to prevent participants from becoming anchored to the selector button’s point of origin. [↑](#footnote-ref-1)
2. Participants were always asked for the probability that the next outcome would be Red/Up, regardless of the pattern of outcomes in the present sequence. [↑](#footnote-ref-2)
3. Red/Up always appeared as the top radial, and Blue/Down always appeared as the bottom radial. [↑](#footnote-ref-3)
4. Two filler sequences had an alternation rate of 1.00, and contained no streaks. These two filler sequences were excluded from this analysis. [↑](#footnote-ref-4)
5. Several of the filler sequences with a maximum Streak Length of 2 contained more than one streak of length 2. In these cases, we considered the streak occurring closest to the end of the sequence as the focal streak in the analysis. Focal streaks are highlighted in bold type in the first column of each summary table. [↑](#footnote-ref-5)
6. Note that in the standard logistic model, there does not appear to be a residual specified. These residuals are “hidden,” in the sense that we implicitly assume there is some continuously distributed *y*_it_* that we cannot directly observe, instead we only observe whether the value of *y*_it_* is positive (1) or negative (0). We assume that this hidden outcome variable follows a linear model that depends on the same predictors as in the logistic model *and* a hidden residual *e*_it_* that is logistically distributed. [↑](#footnote-ref-6)
7. Another option would be to use exchangeable or autocorrelation types of correlation matrix structures. In the former case, the correlation between any pair of residual terms is assumed equal to any other pair. In the latter, the correlation between each pair of residual terms *e_it_*, *e_it-n_* is decreasing in *n*. We initially chose the autocorrelation structure, because it seems reasonable to assume the correlation between the log-odds a participant predicts a streak of length 7 will *repeat* is likely to be highly correlated with the log-odds a participant predicts a streak of length 6 will *repeat*, but less likely to be highly correlated with the participant’s prediction for streaks of length 5 and smaller. There was no difference between the estimates of the model with autocorrelation of error terms and unstructured correlation of error terms. Following Szmaragd et al. (2013) we chose the unstructured model as it is preferred over the alternatives whenever there are few enough observations that this is computationally feasible. [↑](#footnote-ref-7)
8. I treat Streak Length as a nominal variable, because within the GEE family of models Stata doesn't have a specific facility for ordinal variables, and the documentation says to just treat ordinals as nominals. [↑](#footnote-ref-8)
9. Separation was observed while running participant-level logistic regressions on 127/300 participants’ predictions over target sequences.  Firth’s procedure was applied to all of the participant-level logistic regressions to resolve the separation issue, producing less biased coefficients (for an explanation of this procedure, see Heinze & Schemper, 2002). [↑](#footnote-ref-9)
10. We first exponentiate the coefficient to obtain the odds ratio, then we subtract 1 from the odds ratio and multiply by 100 to get the percent-change in the odds: [(*e^β^* – 1) × 100]. Example: The log odds that Participant A predicts “repeat” increase by 0.78 for each unit increase in Streak Length.  The odds that this participant predicts a streak will repeat are exp(0.78) = 2.18 times higher for each unit increase in Streak Length.  The percent-change in the odds this participant predicts “repeat” is [(2.18–1) ✕ 100] = 118% for each unit increase in Streak Length. [↑](#footnote-ref-10)
11. Separation was observed while running participant-level logistic regressions on 83/301 participants’ predictions over target sequences.  Firth’s procedure was applied to all of the participant-level logistic regressions to resolve the separation issue. [↑](#footnote-ref-11)
12. We speculate this pattern results from the prevalence of contradictory beliefs about the stock market. [↑](#footnote-ref-12)
13. Separation was observed while running participant-level logistic regressions on 92/300 participants’ predictions over target sequences.  Firth’s procedure was applied to all of the participant-level logistic regressions to resolve the separation issue. [↑](#footnote-ref-13)
